# Supplementary material for: Cohort profile: the Mendelian randomisation in pregnancy (MR-PREG) collaboration – improving evidence for prevention and treatment of adverse pregnancy and perinatal outcomes
Source: BMJ Open. 2026 Mar 24;16(3):e103753. doi: 10.1136/bmjopen-2025-103753 (PMC13158659; doi:10.1136/bmjopen-2025-103753)
Supplement: Supplementary data [file bmjopen-16-3-s002.pdf]

The Mendelian Randomization in Pregnancy (MR-PREG) collaboration: Improving evidence on prevention, prediction and treatment of adverse pregnancy and perinatal outcomes

Supplementary figures

Avon Longitudinal Study of Parents and Children

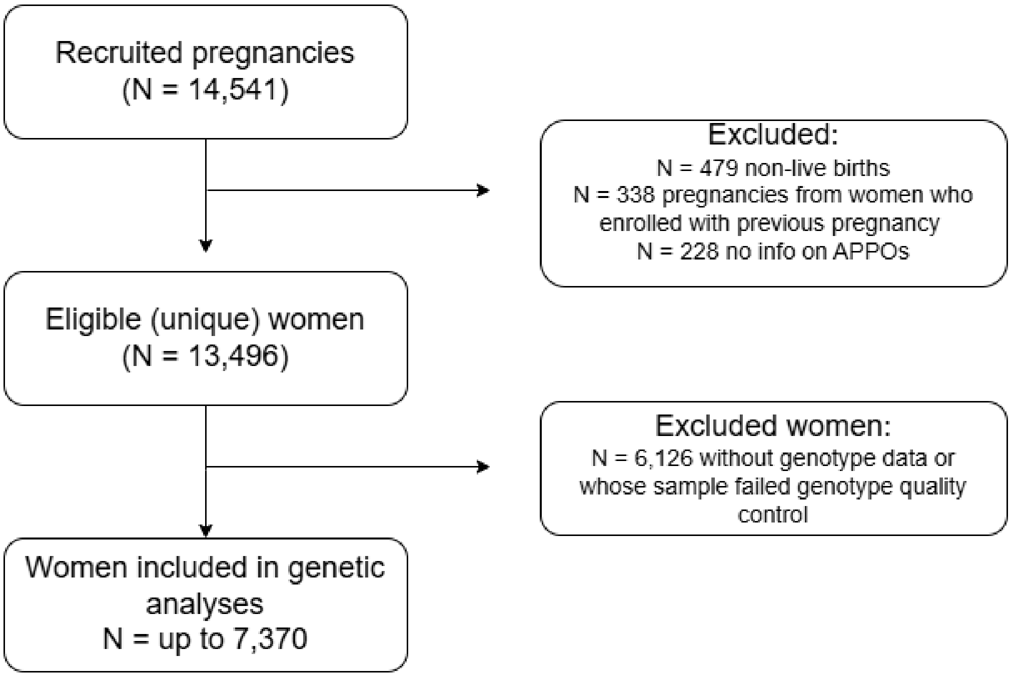

Genetic data is available for a subsample of mothers

Born in Bradford

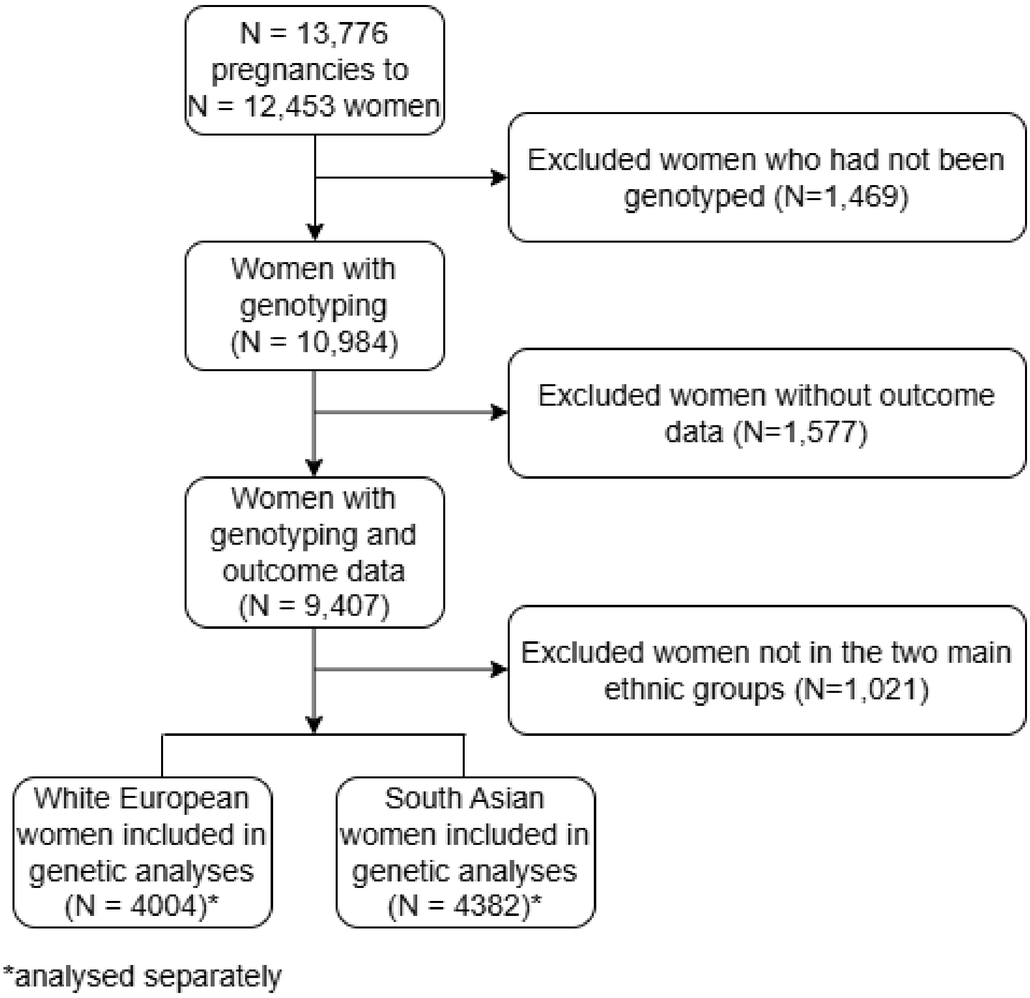

Norwegian Mother Father and Child Birth Cohort

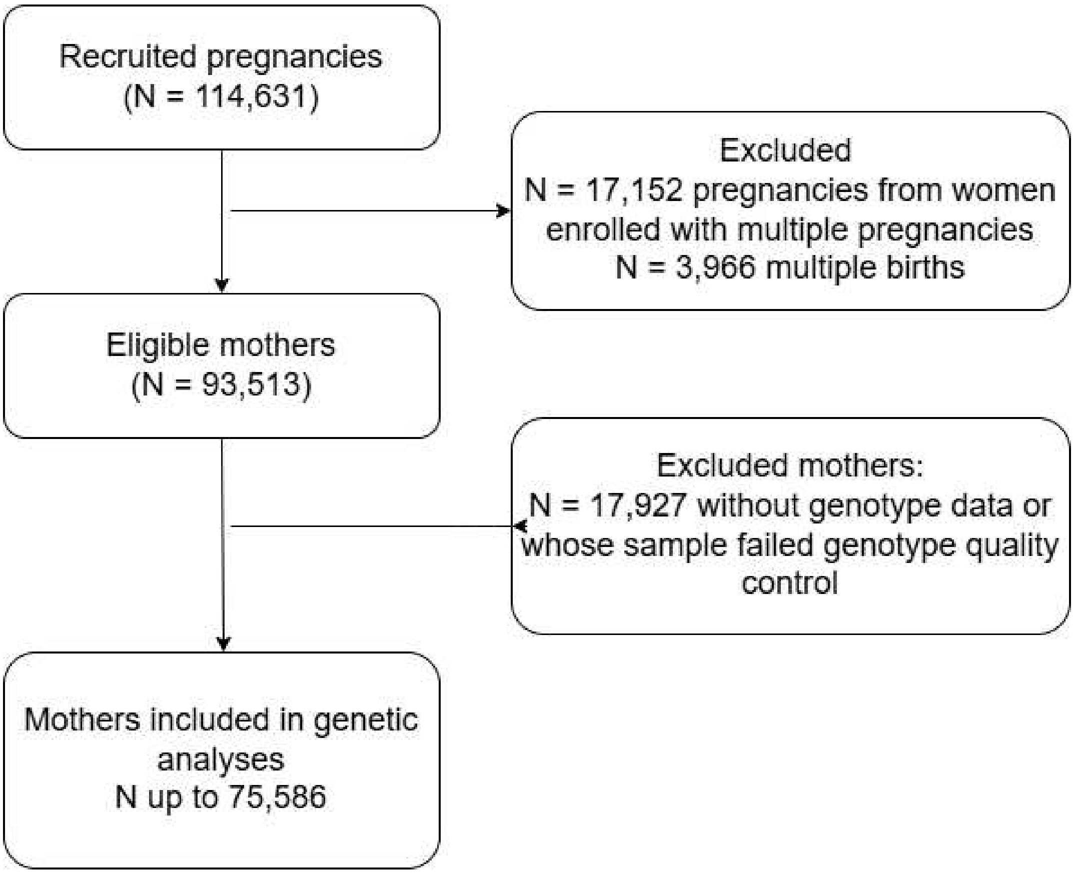

UK Biobank

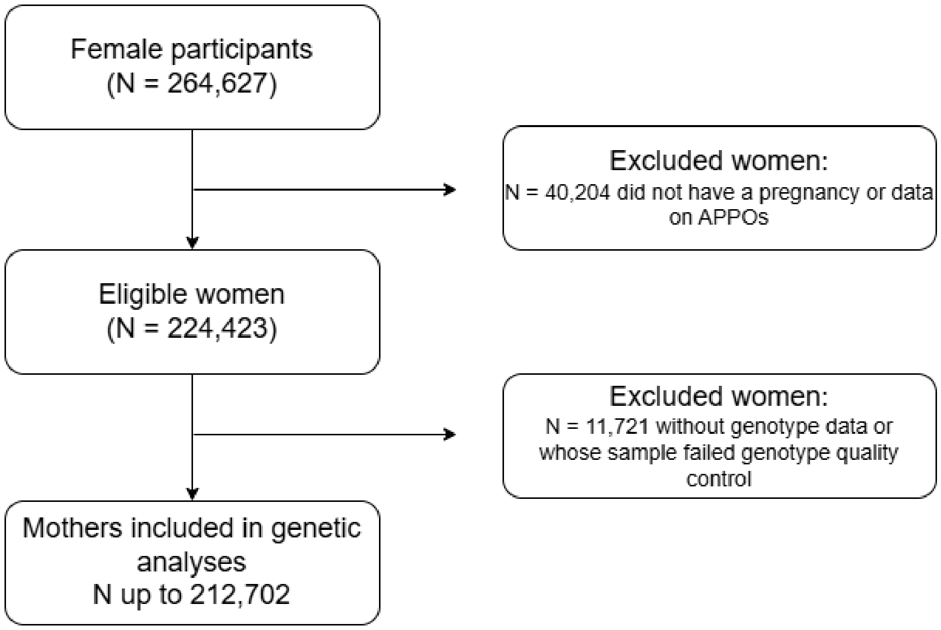

Note: some phenotypes have much smaller sample size because the source of information was maternity records (N up to 8,494), and these were not available for all women
