# Supplementary material for: Cohort profile: the Mendelian randomisation in pregnancy (MR-PREG) collaboration – improving evidence for prevention and treatment of adverse pregnancy and perinatal outcomes
Source: BMJ Open. 2026 Mar 24;16(3):e103753. doi: 10.1136/bmjopen-2025-103753 (PMC13158659; doi:10.1136/bmjopen-2025-103753)
Supplement: Author's manuscript [file bmjopen-2025-103753.draft_revisions.pdf]

BMJ Open is committed to open peer review. As part of this commitment we make the peer review history of every article we publish publicly available.

When an article is published we post the peer reviewers' comments and the authors' responses online. We also post the versions of the paper that were used during peer review. These are the versions that the peer review comments apply to.

The versions of the paper that follow are the versions that were submitted during the peer review process. They are not the versions of record or the final published versions. They should not be cited or distributed as the published version of this manuscript.

BMJ Open is an open access journal and the full, final, typeset and author-corrected version of record of the manuscript is available on our site with no access controls, subscription charges or pay-per-view fees (http://bmjopen.bmj.com).

If you have any questions on BMJ Open's open peer review process please email
info.bmjopen@bmj.com

Cohort Profile: The Mendelian Randomization in Pregnancy (MR-PREG) collaboration - Improving evidence for prevention and treatment of adverse pregnancy and perinatal outcomes

Journal:	*BMJ Open*
Manuscript ID	bmjopen-2025-103753
Article Type:	Cohort profile
Date Submitted by the Author:	15-Apr-2025

Complete List of Authors:	McBride, Nancy; MRC Integrative Epidemiology Unit at the University of Bristol<br>Clayton, Gemma; MRC Integrative Epidemiology Unit at the University of Bristol<br>Soares, Ana Luiza; University of Bristol, Population Health Sciences<br>Yang, Qian ; MRC Integrative Epidemiology Unit at the University of Bristol; Shanghai Jiao Tong University School of Medicine, Department of Endocrine and Metabolic Diseases; Shanghai Jiao Tong University School of Medicine<br>Bond, Tom A; MRC Integrative Epidemiology Unit at the University of Bristol; University of Queensland<br>Taylor, Amy; MRC Integrative Epidemiology Unit at the University of Bristol; University College London<br>Chatzigeorgiou, Charikleia; MRC Integrative Epidemiology Unit at the University of Bristol<br>Aiton, Elisabeth; MRC Integrative Epidemiology Unit at the University of Bristol<br>West, Jane; University of Sheffield, Population Health; Bradford Teaching Hospitals<br>Magnus, Maria; Norwegian Institute of Public Health<br>Lawlor, Deborah; MRC Integrative Epidemiology Unit at the University of Bristol<br>Borges, Maria Carolina ; MRC Integrative Epidemiology Unit at the University of Bristol

Keywords:	Pregnancy, EPIDEMIOLOGY, Mendelian Randomization Analysis, GENETICS

SCHOLARONE™
Manuscripts

BMJ

I, the Submitting Author has the right to grant and does grant on behalf of all authors of the Work (as defined in the below author licence), an exclusive licence and/or a non-exclusive licence for contributions from authors who are: i) UK Crown employees; ii) where BMJ has agreed a CC-BY licence shall apply, and/or iii) in accordance with the terms applicable for US Federal Government officers or employees acting as part of their official duties; on a worldwide, perpetual, irrevocable, royalty-free basis to BMJ Publishing Group Ltd ("BMJ") its licensees and where the relevant Journal is co-owned by BMJ to the co-owners of the Journal, to publish the Work in this journal and any other BMJ products and to exploit all rights, as set out in our licence.

The Submitting Author accepts and understands that any supply made under these terms is made by BMJ to the Submitting Author unless you are acting as an employee on behalf of your employer or a postgraduate student of an affiliated institution which is paying any applicable article publishing charge ("APC") for Open Access articles. Where the Submitting Author wishes to make the Work available on an Open Access basis (and intends to pay the relevant APC), the terms of reuse of such Open Access shall be governed by a Creative Commons licence – details of these licences and which Creative Commons licence will apply to this Work are set out in our licence referred to above.

Other than as permitted in any relevant BMJ Author's Self Archiving Policies, I confirm this Work has not been accepted for publication elsewhere, is not being considered for publication elsewhere and does not duplicate material already published. I confirm all authors consent to publication of this Work and authorise the granting of this licence.

For peer review only

Cohort Profile: The Mendelian Randomization in Pregnancy (MR-PREG) collaboration - Improving evidence for prevention and treatment of adverse pregnancy and perinatal outcomes

McBride, Nancy [1,2]* [nancy.mcbride@bristol.ac.uk]

Clayton, Gemma L[1,2]* [gemma.clayton@bristol.ac.uk, corresponding author]

Goncalves Soares, Ana[1,2]* [analuiza.soares@bristol.ac.uk]

Yang, Qian[1,2,3,4] [qian.yang@bristol.ac.uk]

Bond, Tom A[1,2,5] [tom.bond@bristol.ac.uk]

Taylor, Amy[1,2,6] [amy.taylor@bristol.ac.uk]

Chatzigeorgiou, Charikleia[1,2] [fb23004@bristol.ac.uk]

Aiton, Elisabeth[1,2] [e.aiton@bristol.ac.uk]

West, Jane[7,8] [jane.west@bthft.nhs.uk]

Magnus, Maria C[9] [mariachristine.magnus@fhi.no]

Lawlor, Deborah A[1,2]** [d.a.lawlor@bristol.ac.uk]

Borges, Maria Carolina[1,2]** [m.c.borges@bristol.ac.uk]

*Joint first author

**Joint last author

Corresponding author: Gemma L Clayton, gemma.clayton@bristol.ac.uk, MRC Integrative Epidemiology Unit, University of Bristol, Oakfield House, Oakfield Grove, Bristol, BS8 2BN, United Kingdom, Tel: +44 (0)117 331 0098

Keywords: Pregnancy, epidemiology, mendelian randomisation, triangulation, causal inference

Word count: 4386

For peer review only

Affiliations

[1]MRC Integrative Epidemiology Unit at the University of Bristol

[2]Population Health Science, Bristol Medical School, University of Bristol

[3]Department of Endocrine and Metabolic Diseases, Shanghai Institute of Endocrine and Metabolic Diseases, Ruijin Hospital, Shanghai Jiao Tong University School of Medicine, Shanghai, China

[4]Shanghai National Clinical Research Center for Metabolic Diseases, Key Laboratory for Endocrine and Metabolic Diseases of the National Health Commission of the PR China, Shanghai Key Laboratory for Endocrine Tumor, Lifecycle Health Management Center, Ruijin Hospital, Shanghai Jiao Tong University School of Medicine, Shanghai, China

[5]Frazer Institute, University of Queensland, Woolloongabba, Australia

[6]Division of Surgery and Interventional Science, University College London, London

[7]Population Health, School of Medicine and Population Health, University of Sheffield

[8]Bradford Institute for Health Research, Bradford Teaching Hospitals, Bradford, UK; Department of Health Sciences, University of York, York, UK

[9]Centre for Fertility and Health, Norwegian Institute of Public Health, Oslo, Norway.

For peer review only

Abstract

Purpose

Adverse pregnancy and perinatal outcomes (APPOs), including pre-term birth, pre-eclampsia, and gestational diabetes, can result in maternal and neonatal morbidity and mortality, parental anxiety, and increased health care costs. Better understanding of causes of APPOs is essential to inform lifestyle and pharmaceutical interventions for their prevention and management. Given the difficulty of undertaking randomised control trials in pregnant women, triangulating evidence from across different methods with different sources of bias could improve our understanding of the causes of APPOs. The purpose of the Mendelian Randomization in Pregnancy (MR-PREG) collaboration is to support triangulation of evidence from genetic (e.g., Mendelian randomization [MR]) and non-genetic (e.g., partner negative controls) methods to explore causal effects of maternal exposures on a comprehensive set of APPOs

Participants

The MR-PREG collaboration includes individual participant data from three birth cohorts (two from the UK and one from Norway) and UK Biobank, and summary data from FinnGen and publicly available genome wide association studies (GWAS). We have harmonised data across studies so that currently includes exposures on up to 34 APPOs in up to 678,001 women.

Findings to date

The main aims of MR-PREG are to improve the evidence base for 1) prevention, by advancing our understanding of maternal modifiable causes of APPOs, 2) better understanding of the effect of pre-existing conditions on APPOs, and 3) treatment, by advancing our knowledge of the efficacy and safety of existing medications that women may require for pre-existing conditions, and identifying and testing the efficacy and safety of novel medications, and those that might be repurposed to effectively and safely treat APPOs. To date, our published research mainly addresses aims 1 and 3; some examples include triangulation of evidence from MR, conventional multivariable regression and a paternal negative control, showing that higher maternal body mass index increases the risk of many APPOs, and identification of maternal circulating metabolites and proteins that may influence birthweight.

For peer review only

Future Plans

Our future priorities include increasing diversity in the MR-PREG collaboration by expanding participant representation from non-European ancestries. We are also integrating molecular data, such as circulating protein levels and placental transcriptomics, to better understand the molecular mechanisms underlying APPOs. Additionally, we are using exome and whole-genome sequencing to identify novel causal genes for APPOs and advance our knowledge on candidate targets for APPOs.

Strengths and Limitations

- We have curated data for 34 APPOs and harmonized data across multiple studies to support large-scale investigations of causes of APPOs.
- The scope and type of the data supports triangulation of evidence from a range of genetic and non-genetic methods, with different unrelated sources of bias, to identify causes of APPOs.
- Over the coming year we will enhance the data to enable identification of molecular mechanism underlying APPOs.
- MR-PREG has limited power to detect causal effects on rarer APPOs, such as congenital anomalies and low Apgar scores at 5 minutes, particularly when using genetic methods. Future work will include larger samples and rare genetic variant data.
- Participants are mostly of European ancestry; future efforts will be focussed on diversifying the ancestry representation in the data.

For peer review only

Introduction

A substantial proportion of pregnant women experience adverse pregnancy or perinatal outcomes (APPOs), such as miscarriage, stillbirth, gestational diabetes mellitus (GDM), gestational hypertension (GH), pre-eclampsia (PE), pre-term birth (PTB), or having a small (SGA) or large for gestational age (LGA) baby. Globally, one in six recognised pregnancies results in miscarriage [1], one in six women develops GDM [2], one in ten women has hypertension during pregnancy [3], and one in ten babies is born preterm [4]. Some APPOs are causes of severe morbidity and mortality for mothers and their babies [5]. For illustration, hypertensive disorders of pregnancy (HDP), including GH and PE, account for approximately 14% of all maternal deaths worldwide [6]. In addition, APPOs are associated with long-term adverse physical and mental health outcomes, and high costs to families, healthcare systems and society [7 8]. In the UK, the short-term costs of miscarriage alone are estimated to be around £471 million per year due to costs to health services, families and loss of productivity [1].

The marked variation in the prevalence of some APPOs across regions and over time, although influenced by different screening and diagnostic practices, indicates that they are preventable [2]. Better understanding the underlying causes of APPOs is crucial for guiding effective interventions to prevent them. This knowledge would also enable antenatal care services to provide women and couples with accurate advice on the most plausible risk factors for reducing APPOs, potentially reducing the often conflicting advice given to women about exposures that could impact their and their babies' health during pregnancy. Furthermore, APPOs are often related, meaning that preventing one may help reduce the burden of others. For example, both GDM and HDP can influence fetal growth, leading to the delivery of LGA and SGA babies, respectively [9 10]. Therefore, preventing GDM and HDP is a way of avoiding fetal over/under growth and the related complications.

There is also an urgent need to better understand the effects of pharmaceutical treatments during pregnancy, both for preventing and managing APPOs and for treating pre-existing conditions that are increasingly common among women of reproductive age, such as autoimmune, mental health, thyroid, reproductive, and cardiometabolic disorders [11-14]. An increasing number of women start pregnancy on medication, and the use of medication among pregnant women has also been rising over the past few decades [15]. Despite that, pregnant women are rarely included in pre-licensure randomized controlled trials (RCTs) due

For peer review only

to the concerns about potential teratogenic effects of drugs and the potential impact of drug-dosing on the physiological changes of pregnancy [16 17]. As a result, evidence on the benefits and risks of drugs for mother and baby is poor, investment in new drug development for APPOs is scarce, and very few medications are explicitly licensed for use during pregnancy. This leaves mothers and their babies exposed to unknown risks of drugs, compels pregnant women and their doctors to undertreat medical conditions, and forces them to make difficult decisions about which medications to prescribe/maintain and at which dose. It also means that APPOs, such as GDM and GH, are managed less well than equivalent conditions outside of pregnancy (i.e., type 2 diabetes and hypertension) [16 17].

RCTs are the gold standard method for testing the impact of lifestyle and pharmaceutical interventions to prevent or treat APPOs. For several years, national bodies have highlighted the need for clinical trials of medications in pregnant women, largely to no avail [16 18]. Acknowledging this, the UK 2021 Report of the Commission on Human Medicines Expert Working Group on Optimising Data On Medicines Used During Pregnancy recommended i) the use of routine healthcare data in research, and ii) the use of observational research to investigate safety of medicines used in pregnancy and during breastfeeding should be commissioned and a system of ongoing surveillance should be established and funded [18].

In the absence of well-powered, well-conducted RCTs, we need to make the best use of observational data to improve the current evidence base on causes of APPOs and medication efficacy and safety during pregnancy. More than 20 years ago, the use of genetic variants to infer causal effects of exposures − a method known as Mendelian randomization (MR) − was first proposed [19]. MR leverages the random allocation of genetic variants at conception to investigate the effects of modifiable risk factors on health outcomes. This method mitigates confounding by socioeconomic, behavioural, or health-related factors that frequently affect traditional observational study analyses (**Figure 1**) [19-21]. Since then, its application has expanded significantly, including for assessing the effects of a few maternal risk factors on a small range of APPOs [22 23 24].

For peer review only

Figure 1 Summary of the Mendelian randomisation assumptions to estimate the presence of an effect of maternal risk factors on adverse pregnancy and perinatal outcomes

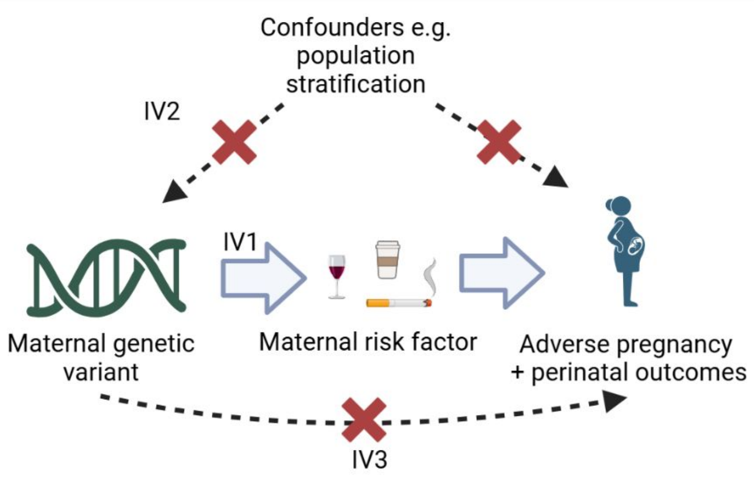

<u>Assumptions</u>

- The maternal genetic variant(s) are statistically robustly associated with the maternal risk factor during pregnancy **[relevance assumption, IV1]**
- There is no confounding of the maternal genetic variant(s) and adverse pregnancy and perinatal outcomes (APPOs) (i.e., population level confounders such as population structure, assortative mating, and intergenerational effects) [25] **[independence assumption, IV2]**
- The maternal genetic variant is not associated with APPOs other than through its association with the maternal risk factor **[exclusion restriction criteria, IV3]**

peer review only

In addition, MR is increasingly used for assessing the effects of medications outside of pregnancy (aka drug target MR). The validity of drug target MR is supported by proof-of-concept studies comparing MR findings to RCT results for established medications, such as antihypertensives and statins [26]. Drug targets validated by genetic evidence have higher rates of success and MR is increasingly used to prioritise (or de-prioritise) new drug targets to be tested in RCTs [27-29].

As with all methods, MR is limited by violation of its assumptions. Triangulation of evidence acknowledges, and exploits the fact that all methods have sources of bias [30]. It involves

integrating multiple lines of evidence using one or more different approaches (e.g., different analytical methods, different data-sources, or different study designs) with different and unrelated key sources of bias. If results are consistent, despite the different sources of bias, this increases the credibility of that being the correct causal effect (whether it suggests a protective, detrimental or no effect), as it would be unlikely for different biases to produce different results. Where there is disagreement across the different methods, prior specification of key sources of bias, the direction of these biases, and statistical efficiency for each can help inform whether the inconsistency is explained by the different biases or power for each approach, which can inform what further sensitivity analyses and/or other approaches are needed to obtain a valid causal estimate.

The Mendelian Randomization in Pregnancy (MR-PREG) collaboration was established to improve causal understanding of the effects of maternal lifestyle and health factors on APPOs, as well as efficacy and safety of medication use in pregnancy. In this paper, we provide the aims of the MR-PREG collaboration, describe the characteristics of the contributing studies, our methods for data generation and exploring causal effects, as well as summarising findings to date and discussing ongoing and future work.

Aim 1: Using triangulation of evidence to improve knowledge on the impact of maternal lifestyle factors on APPOs

The MR-PREG collaboration uses triangulation of genetic methods (e.g., MR, co-localisation, rare variant, and joint rare and common variant analyses) and non-genetic methods (e.g., conventional multivariable regression, and negative paternal controls) to explore the effect of a range of maternal modifiable lifestyle factors, such as smoking, alcohol intake, coffee consumption, adiposity, sleeping habits, and physical activity, on the risk of multiple APPOs (**Figure 2**) (**Supplementary Table 1**) [30].

Aim 2: Better understanding the effect of maternal predisposition to conditions, such as autoimmune, cardiovascular, hormonal, musculoskeletal, and mental health conditions, on APPOs

An increasing number of women begin pregnancy with one or more pre-existing conditions that may elevate the risk of multiple APPOs [12]. A systematic understanding of the overall impact of these conditions on APPO risk is important for guiding clinical decision-making in

For peer review only

the management and treatment of such conditions during pregnancy. Within the MR-PREG collaboration, we employ MR to investigate how genetic predisposition to common conditions in women of reproductive age - such as autoimmune, cardiovascular, hormonal, musculoskeletal, and mental health conditions - affects APPO risk (**Figure 2**).

Aim 3: Leveraging genomics to explore drug efficacy and safety of medications in pregnancy

The MR-PREG collaboration aims to improve the evidence on the benefits and risks of medications during pregnancy using drug target MR. We investigate potential effects of medications for mothers and their babies by investigating the efficacy and safety in pregnancy of medications used to treat pre-existing conditions, discovering new candidate drug targets to prevent/treat APPOs, and identifying opportunities to repurpose existing drugs, designed to treat conditions unrelated to pregnancy, to prevent/treat APPOs (**Figure 2**).

Figure 2 MR-PREG collaboration aims

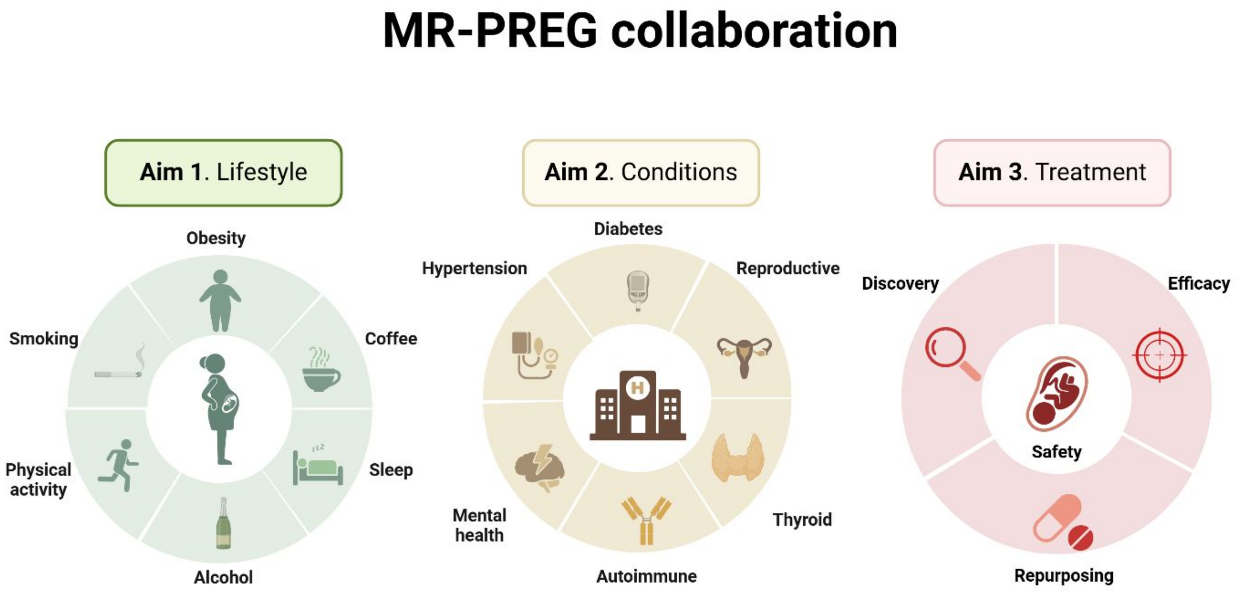

For peer review only - http://bmjopen.bmj.com/site/about/guidelines.xhtml

Cohort description

Adverse pregnancy and perinatal outcomes (APPOs)

A key focus of the MR-PREG collaboration is on assessing the causal effect of exposures on multiple outcomes simultaneously, so that we have a more complete picture of the potential beneficial, detrimental, or null effect of an exposure on APPOs. This 'outcome-wide epidemiology' approach [31] is informative so that women, their partners and healthcare providers have a more balanced view of the potential adverse and beneficial effects of risk factors than what might be obtained from studies that focus on a single or small number of outcomes.

We have included 34 binary/categorical outcomes that can occur in pregnancy, delivery or the first year after birth, and 2 underlying continuous traits in MR-PREG. **Table 1** shows the APPOs and underlying traits available in MR-PREG together with the number of women who have phenotypic and genetic data on each of these and the number of cases for the outcomes. Cohort-specific APPOs definitions and exclusions are provided in supplementary material (**Supplementary Tables 2A** and **2B)**, and as well as sample size for binary/categorical outcomes (**Supplementary Table 3A** and **3B** **f**or maternal phenotype and genetic data, respectively, and **Supplementary Table 3C** for offspring genetic data), and mean and standard deviations for continuous traits (**Supplementary Table 3D**). **Supplementary Tables 4A** and **4B** describe any deviations from the collaboration definition for each study.

For peer review only

Table 1. Adverse pregnancy and perinatal outcomes (APPOs) and underlying traits, total available sample and respective number of cases

Type	Outcomes/Traits	Total	Cases N (%)
Pregnancy	Hypertensive disorders of pregnancy	541,768	32,549 (6.0%)
	Gestational hypertension	527,932	20,777 (3.9%)
	Pre-eclampsia	678,001	19,566 (2.9%)
	Gestational diabetes	934,566	26,346 (2.8%)
	Depression	147,601	22,785 (15.4%)
	Anaemia	86,167	4,105 (4.8%)
	Pregnancy loss	298,868	94,152 (31.5%)
	Miscarriage	486,217	89,086 (18.3%)
	Sporadic miscarriage	241,159	55,888 (23.2%)
	Recurrent miscarriage	321,756	6,233 (1.9%)
	Stillbirth	207,670	6,331 (3.0%)
	Hyperemesis	478,466	4,235 (0.9%)
	Severity of nausea and vomiting	82,978	
	Nausea only		7,166 (8.6%)
	Nausea and vomiting		30,133 (36.3%)
	On medication		31,210 (37.6%)
Delivery	Induction of labour	95,239	14,495 (15.2%)
	Premature rupture of membranes	310,621	21,839 (7.0%)
	Caesarean section	233,969	33,909 (14.5%)
	Emergency C-section	90,438	9,165 (10.1%)
	Elective C-section	87,283	6,004 (6.9%)
	Very pre-term birth	85,274	1,214 (1.4%)
	Pre-term birth	518,849	29,987 (5.8%)
	Post-term birth	425,673	27,841 (6.5%)
	Low birth weight	275,214	18,617 (6.8%)
	High birth weight	266,835	6,679 (2.5%)
	Small for gestational age	89,033	6,882 (7.7%)
	Large for gestational age	96,305	10,468 (10.9%)
	Low Apgar score at 1 minute	86,668	4,950 (5.7%)
	Low Apgar score at 5 minutes	78,739	876 (1.1%)
Postnatal	NICU admission	77,285	6,996 (9.1%)
	Congenital anomalies	74,701	3,569 (4.8%)
	Congenital heart disease	74,701	612 (0.8%)
	Breastfeeding initiation	84,668	66,408 (78.4%)
	Breastfeeding established	81,258	58,035 (71.4%)
	Breastfeeding sustained	65,189	44,401 (68.1%)
	Breastfeeding duration	82,429	
	1 to 3		6,372 (7.7%)
	4 to 6		49,123 (59.6%)
	>6		3,419 (4.1%)
Underlying traits	Birth weight*	289,846	-
	Gestational age*	226,810	-

* continuous traits

For peer review only

Data sources

Currently, the MR-PREG collaboration includes data from four core studies, which comprise three prospective birth cohorts [the Avon Longitudinal Study of Parents and Children (ALSPAC), Born in Bradford (BiB), and the Norwegian Mother, Father and Child Cohort Study (MoBa)], and a biobank [UK Biobank (UKB)]. Data from additional sources are also used, such as from publicly available biobanks (FinnGen) and genome-wide association studies (GWAS) meta-analyses, described below.

Core studies

A brief description of each of the core studies is presented below and the participant characteristics are summarised in **Table 2**. **Supplementary Figures 1-4** show the flow of participants from recruitment into their cohorts to inclusion in our analyses. The APPOs each study contributed to MR-PREG are summarised in **Supplementary Tables 2A and 2B**. Details on genotyping in each study are presented in supplementary material.

For peer review only

For peer review only - http://bmjopen.bmj.com/site/about/guidelines.xhtml

Table 2. Maternal participant characteristics in the participating core studies#

Characteristics	ALSPAC	BiB	MoBa	UK Biobank
Total N	13,895	9,407	93,512	224,423
Age, mean (SD)	28.0 (5.0)	27.4 (5.6)	30.1 (4.7)	25.3 (4.6)*
Missing	369 (2.7%)	1,169 (12.4%)	458 (0.5%)	45,402 (20.2%)
Ethnicity (White), N (%)	11,600 (97.4%)	3,430 (36.4%)	88,989 (95.1%)	211,677 (94.6%)
Missing	1,982 (14.3%)	1,025 (10.1%)	2,021 (2.1%)	706 (0.3%)
Education (university level), N (%)	1,544 (12.9%)	1,432 (11.8%)	50,047 (53.5%)	65,334 (29.5%)**
Missing	1,890 (13.6%)	1,186 (12.6%)	11,064 (11.8%)	2,714 (1.2%)
Index of multiple deprivation, (N%)				
1st quintile (most deprived)	1,628 (13.9%)	5,232 (55.6%)	NA	44,857 (20.0%)§
5th quintile (least deprived)	3,045 (26.0%)	158 (1.7%)	NA	44,805 (20.0%)
Missing	2,197 (15.8%)	1,171 (12.4%)		384 (0.2%)
Parity (nulliparous), N(%)	5,619 (44. 7%)	3,768 (40.0%)	42,135 (45.1%)	179,385 (83.6%)
Missing	1,333 (9.6%)	345 (3.6%)	399 (0.4%)	9,762 (4.3%)
Smoking during pregnancy (ever), N (%)	3,301 (25.9%)	1,408 (14.9%)	5,939 (6.4%)	NA
Missing	1,124 (8.1%)	1,185 (12.6%)	16,824 (18.0%)	
Alcohol intake during pregnancy, N (%)	8,036 (64.0%)	1,707 (18.1%)	2,472 (2.6%)	NA
Missing	1,340 (9.6%)	2,114 (22.9%)	27,648 (29.6%)	
Body mass index, mean (SD)	22.9 (3.8)	26.1 (5.7)	24.1 (4.3)	27.1 (5.1)**
Missing	2,711 (19.5%)	1,562 (16.6%)	11,561 (12.3%)	888 (0.4%)

Participants included if they had data on at least one APPO
* age at first birth; ** information assessed at recruitment and not at pregnancy; § this corresponds to deprivation index and quintiles were constructed internally (not based on national comparisons)
Abbreviations: ALSPAC, The Avon Longitudinal Study of Parents and Children; BiB, Born in Bradford; MoBa, The Norwegian Mother, Father and Child cohort study; SD, standard deviation; NA, information not available in this study. This includes all available data at the outcome level.

For peer review only - http://bmjopen.bmj.com/site/about/guidelines.xhtml

ALSPAC: The Avon Longitudinal Study of Parents and Children

The Avon Longitudinal Study of Parents and Children (ALSPAC) is a prospective birth cohort that started recruiting pregnant women resident in the former county of Avon (centred around the city of Bristol), with delivery dates between April 1991 and December 1992. A total of 14,541 women (ALSPAC-G0) were enrolled during pregnancy (14,676 fetuses) and gave birth to 14,062 live children (ALSPAC-G1) [32 33]. Women responded to four questionnaires during pregnancy (average 8, 12, 18, and 32 weeks gestation) and two postpartum (average 8 weeks and 8 months). Biological samples were collected during pregnancy (blood and urine) and birth (cord blood and placenta). Maternal anthropometrics was based on self-report collected at 12 weeks gestation and children had anthropometrics measured at birth. Obstetric records were also linked to the participants. Genetic data is available for mothers, partners and children (details on genotyping array and imputation are presented in supplementary material). Children of the children (ALSPAC-G2) are also being assessed and followed-up, but this data has not yet been included in MR-PREG. The study website contains details of all the data available through a fully searchable data dictionary and variable search tool: http://www.bristol.ac.uk/alspac/researchers/our-data/. Ethical approval for the study was obtained from the ALSPAC Ethics and Law Committee and the Local Research Ethics Committees. Full details of the ALSPAC consent procedures are available on the study website (http://www.bristol.ac.uk/alspac/researchers/research-ethics/http://www.bristol.ac.uk/alspac/researchers/research-ethics/).

BiB: Born in Bradford

Born in Bradford (BiB) is a prospective birth cohort that recruited women with expected delivery dates between March 2007 and December 2010. Most women were recruited at their oral glucose tolerance test (OGTT) at approximately 26–28 weeks' gestation, which was offered to all women booked for delivery at Bradford Royal Infirmary, except those with known diabetes, during the recruitment period. In BiB, most of the obstetric population consists of women of White British or Pakistani origin (together accounting for 81%, with the remaining women being of other ancestries). A total of 12,453 women (13,776 pregnancies) were enrolled during pregnancy who gave birth to 13,858 live children. Women had anthropometrics measured at recruitment and responded to questionnaires during pregnancy and postpartum to provide information about breastfeeding > 6 months. Biological

For peer review only - http://bmjopen.bmj.com/site/about/guidelines.xhtml

samples were collected during pregnancy (blood and urine) and birth (cord blood) and women consented to routine primary and secondary care data linkage. Full details of the study methodology were reported previously [34]. Genetic data is available for mothers and children (details on genotyping array and imputation presented in supplementary material). Ethical approval for the study was granted by the Bradford National Health Service Research Ethics Committee (ref 06/Q1202/48). The study website provides further cohort details and an overview of available data (https://borninbradford.nhs.uk/).

MoBa: The Norwegian Mother, Father and Child Cohort Study

The Norwegian Mother, Father and Child Cohort Study (MoBa) is a prospective birth cohort that recruited pregnant women from all over Norway from 1999-2008. The cohort includes approximately 95,200 mothers, 75,200 fathers, and 114,500 children. Mothers and their partners responded to questionnaires during pregnancy (15, 22, and 30 weeks gestation) and postpartum (6 months). Blood samples were obtained from both parents during pregnancy and from mothers and children (cord blood) at birth. Maternal anthropometrics were collected from a questionnaire at 15 weeks gestation. Data was linked to the Medical Birth Registry (MBRN), which is a national health registry containing information about all births in Norway. The current study is based on version 12 of the quality-assured data files released for research in 2019. Genetic data is available for mothers, children and partners (details on genotyping array and imputation presented in supplementary material). The establishment of MoBa and initial data collection was based on a license from the Norwegian Data Protection Agency and approval from The Regional Committees for Medical and Health Research Ethics. The MoBa cohort is currently regulated by the Norwegian Health Registry Act. Ethical approval for our study was obtained from The Regional Committees for Medical and Health Research Ethics (ref 2018/1256).

UKB: UK Biobank

UK Biobank (UKB) is an adult cohort that retrospectively collected relevant data on APPOs. All people in the UK National Health Service (NHS) registry aged between 40-69 years and living within an approximately 25-mile radius from one of the 22 study centres were invited to participate in UKB between 2006-2010 [35 36]. A total of 500,000 adults (5.5% of the ~9.2 million invited) were recruited into the study (54.4% females). Information was assessed at baseline

For peer review only - http://bmjopen.bmj.com/site/about/guidelines.xhtml

via a self-completed questionnaire, physical measures (including anthropometrics), and collection of non-fasting blood, urine, and saliva. Participants have been followed up by linkage to electronic health records and a subset of participants responded to online questionnaires. Hospital Episode Statistics (HES) from 1997 for England, 1998 for Wales and 1981 for Scotland are available [37]. HES data also contain maternity-related admissions for England and Wales. It is important to note that not every participant has a hospital inpatient record, as not all have been admitted to hospital within the period covered. Genetic data from UKB participants is available (details on genotyping array and imputation presented in supplementary material). Ethical approval for UKB was obtained from the Northwest Multi-Centre Research Ethics Committee (MREC), and MR-PREG collaboration studies are linked to UKB application number 23938. The UKB showcase website contains details of the data available: https://biobank.ndph.ox.ac.uk/showcase/.

Additional data sources

To increase statistical power for MR analyses, the MR-PREG collaboration also uses genetic association data for APPOs from publicly available datasets – i.e., FinnGen and several publicly available GWAS meta-analyses – as described below.

FinnGen

FinnGen is the nationwide network of Finnish biobanks, which are linked to national electronic registries that provide information on prescriptions and diseases (ICD9-10 codes). FinnGen includes data from 500,348 individuals (282,064 females and 218,284 males) [12th data release (R12)]. Besides clinical endpoints, which also include data on some APPOs, genetic data is also available. The APPOs and number of cases and controls for which FinnGen contributed are detailed in **Supplementary Table 3B**. The Coordinating Ethics Committee of the Helsinki and Uusimaa Hospital District has approved the FinnGen consortium (Nr HUS/990/2017). More information about FinnGen can be found on the website https://www.finngen.fi/en. The metadata from FinnGen used by the MR-PREG collaboration is publicly available at https://www.finngen.fi/en/access_results.

GWAS meta-analyses

For peer review only - http://bmjopen.bmj.com/site/about/guidelines.xhtml

At the time of writing, the MR-PREG collaboration has harmonised and quality-controlled data from GWAS meta-analyses for GDM [5,485 cases and 347,856 controls from the GENetics of Diabetes in Pregnancy Consortium (GenDIP) consortium [38]]; PE [9,515 cases and 157,719 controls from the International Pregnancy Genetics (InterPregGen) consortium [39]]; gestational duration-related traits [18,797 PTB cases and 260,246 controls, 15,972 post-term birth cases and 115,307 controls, and 195,555 individuals with gestational age at delivery from the Early Growth Genetics (EGG) consortium [40]]; post-natal depression [17,339 cases and 53,426 controls from the Psychiatric Genomics consortium (PGC) [41]]. Details are provided in **Supplementary Table 5**, including the number of participants, outcome definition and source of data (e.g., electronic health records, maternal report, research data collection), and availability of maternal and/or fetal genetic effects.

Genetic association data for adverse pregnancy and perinatal outcomes

For studies with access to individual-level data (ALSPAC, BiB, MoBa, and UKB), we conducted GWAS analyses to generate genetic association data for APPOs, enabling two-sample MR analyses across the aims of the MR-PREG collaboration. The procedures used for conducting GWAS and quality control in each study are described in detail in the **Supplementary text**. We excluded genetic variants with low imputation accuracy (INFO score < 0.4) and/or low minor allele frequency (MAF < 0.01). In addition, we excluded studies that overlap with public GWAS metanalyses or that contributed with less than 50 cases for a given APPO and applied genomic control to each study. We then pooled study-specific genetic association data on APPOs using inverse variance weighted fixed-effects meta-analyses implemented in METAL (v. 2020-05-05) [42] and estimated the Cochrane's Q statistics to explore between-study heterogeneity. Separate meta-analyses were conducted for maternal and offspring genetic effects. We used GWASInspector to evaluate the quality of GWAS summary data from each GWAS and for the meta-analyses [43].

Conditional estimates

The MR-PREG collaboration is mostly interested in the causal effects of maternal exposures on APPOs. Due to the correlation between maternal and offspring genetics, accounting for offspring genetic effects is crucial to reliably interpret MR findings testing maternal exposure

For peer review only - http://bmjopen.bmj.com/site/about/guidelines.xhtml

effects (**Box 1, Figure 3a** and **Figure 3b**). Genetically instrumented maternal exposure effects (not biased due to exclusion restriction violation via fetal effects) and genetically instrumented fetal effects (not confounded by maternal genotype) can be estimated. To account for mutually adjusted maternal and fetal genetic effects we used a weighted linear model (WLM) implemented using DONUTS (Decomposing nature and nurture using GWAS summary statistics) software [44] to estimate mutually adjusted maternal and fetal genetic effects at each SNP. The WLM calculates conditional genetic effects (i.e., the mutually adjusted coefficients for maternal, offspring and paternal genotype, fitted jointly in the same model) as linear combinations of the marginal genetic effects (i.e., the coefficients for maternal, offspring and paternal genotype, estimated separately in potentially overlapping samples). In MoBa, because we also had genotype data on a large sample of fathers, we did the same to account for paternal genetic effects. Details can be found in **Supplementary text.**

For peer review only - http://bmjopen.bmj.com/site/about/guidelines.xhtml

Box 1 Approaches to check the Mendelian randomisation assumptions (**Figure 1**), specific to maternal risk factors and adverse pregnancy and perinatal outcomes (APPOs). These are further sensitivity methods specific to this scenario, beyond standard MR sensitivity methods such as weighted median and MR-Egger etc.

Relevance

Assumption: The maternal genetic instrument is robustly associated with the maternal exposure during pregnancy

- Many instruments for an exposure are based on non-pregnant phenotypes (e.g., BMI, smoking, alcohol use, or sleep measured outside of pregnancy) rather than pregnancy-specific measures or measures taken during pregnancy
- Genetic variants are lifelong so may not easily capture exposures in specific time frames like pregnancy which bring about widespread physiological and hormonal changes

Recommendations

- We recommend ensuring a strong association between the maternal genetic variant and the pregnancy-specific exposure

Independence

Assumption: There is no confounding of the maternal genetic variant(s) and the outcome (i.e., population level confounders)

- Conventional confounding by socioeconomic position and its related lifestyle and health characteristics cannot confound MR analyses as these cannot cause variation in genetic variants
- Population stratification, assortative mating, intergenerational effects can introduce confounding in genetic studies

Recommendations

- We account for population stratification in each study by adjusting for ancestral principal components or using mixed models
- Family based designs can avoid bias by population stratification, assortative mating, intergenerational effects

Exclusion Restriction

Assumption: The maternal genetic variant is not associated with the outcome other than through its association with the maternal exposure

- One plausible violation of this assumption could be horizontal pleiotropy i.e. where the genetic instrument influences the outcome not through the exposure of interest
- Estimates can also be biased if genetic variants which influence the maternal exposure also influence the outcome when inherited by the offspring (**Figure 3a**)

Recommendations

- Ideally researchers should consider accounting for fetal genotype in MR of maternal exposures
- Adjusting only for offspring genotype may cause collider bias or reflect assortative mating (**Figure 3b**)
- We recommend further adjusting for paternal genotype in sensitivity analyses for later offspring outcomes, though sample sizes may be smaller

For peer review only - http://bmjopen.bmj.com/site/about/guidelines.xhtml

Figure 3a Illustration of how the exclusion restriction assumption may be violated

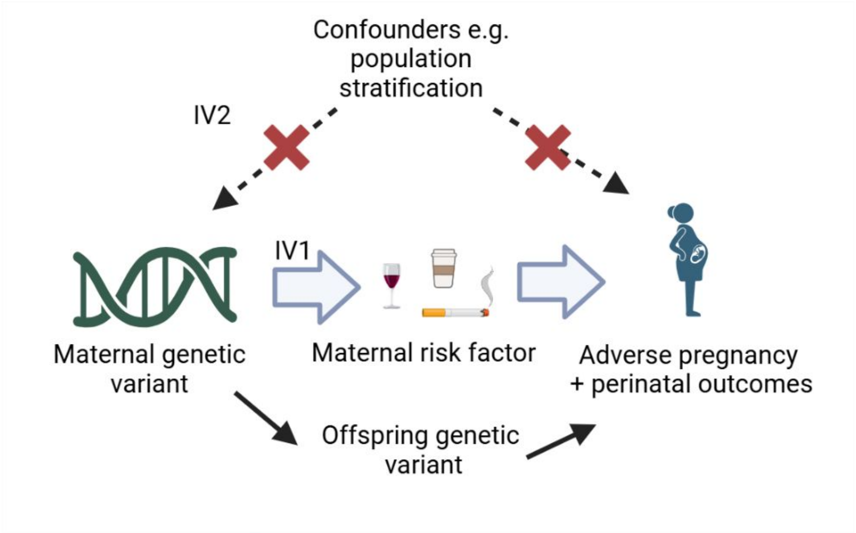

Figure 3b Illustration of how collider bias might be induced in MR analysis

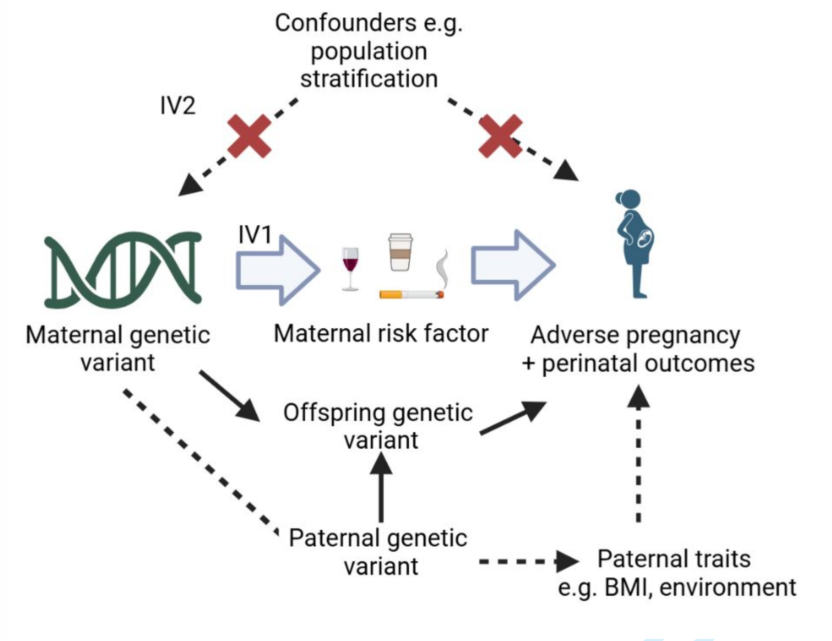

Adjusting for offspring genetic variants only (which are a collider variable) (**Figure 3b**) means that a false association between maternal and paternal genetic variants can be created (shown as a dashed line). Such associations can also arise via assortative mating. This will only cause bias if the outcome is influenced by paternal genetic variants, independently of maternal and offspring genetic variants. However, by additionally adjusting for paternal genetic variants, the pathway highlighted in black dashed arrows can be closed.

For peer review only - http://bmjopen.bmj.com/site/about/guidelines.xhtml

Findings to date

To date there have been 8 peer-reviewed publications [23 45-52] and 1 preprint [53] resulting from the MR-PREG collaboration. We briefly describe a selection of these here to give readers an indication of the different ways in which MR-PREG can be used to explore effects of exposures on APPOs.

Most publications to date align with **Aim 1**, enhancing our understanding of how maternal lifestyle factors influence the risk of APPOs, thereby informing interventions aimed at their prevention. For example, by triangulating evidence from MR, conventional multivariable regression and paternal negative control, we have shown that higher maternal BMI increases the risk of GH, PE, GDM, pre-labour membrane rupture, induction of labour, Caesarean section, LGA, high birthweight, low Apgar score, and admission to a neonatal intensive care unit, and reduced risk of breastfeeding. We also found evidence of higher maternal BMI reducing the odds of SGA and having no effect on perinatal depression [49]. Triangulating evidence from MR and conventional multivariable regression, we found evidence that insomnia may increase the risk of perinatal depression but does not appear to influence most other APPOs that we were able to explore [51]. In a study focussed on fetal growth trajectories, assessed by repeat ultrasound scan measures in two cohorts, we triangulated evidence from MR, conventional multivariable regression and paternal negative control study and found evidence of a consistent linear dose-response association of maternal smoking with fetal growth from early in the second trimester onwards. No major growth deficit was found in women who quit smoking early in pregnancy [54].

In relation to **Aim 3**, we have published preliminary work identifying novel molecular targets (i.e., proteins and metabolites) causally related to APPOs. In one study, we used MR to investigate the effect of 1,139 maternal and fetal circulating proteins on offspring birthweight. We found evidence that higher maternal levels of PCSK1 potentially increase birthweight whilst higher maternal levels of LGALS4 potentially decrease birthweight. Conversely, higher fetal levels of PCSK1 potentially decrease birthweight and of LGALS4 potentially increase birthweight. Higher fetal LEPR increased birthweight. Results support maternal and fetal protein effects on birthweight, implicating roles for glucose metabolism, energy balance, and vascular function [53]. We have also identified maternal circulating

For peer review only - http://bmjopen.bmj.com/site/about/guidelines.xhtml

metabolites beyond glucose that may influence birthweight, such as amino acids like glutamine [46]. In further studies, we have used conventional multivariable regression and MR to explore the potential effect of > 1,000 maternal circulating mass spectrometry metabolites on the risk of offspring congenital heart disease. We found that pregnancy amino acid metabolism, androgenic steroid lipids, and levels of succinylcarnitine could be important contributing factors for congenital heart disease (CHD) [50].

Strengths and Limitations

The collaboration has created curated data for 34 APPOs and harmonised data across several studies to support large-scale investigations of causes of APPOs. In addition, the generation of genetic association data enables well-powered genetic studies, including MR studies, to improve causal knowledge of many key targets for lifestyle and pharmaceutical interventions aimed at preventing APPOs. Data on equivalent exposures in partners and on a wide range of plausible confounders, enables triangulation across genetic and non-genetic approaches, and also appropriate sensitivity analyses. In relation to those sensitivity analyses the collaboration has also derived conditional estimates so that maternal genetic variants can be used as an instrument to test the effect of maternal exposures without biases related to offspring genetic effects.

Despite the size and scale of the data, we are still underpowered to detect causal effects on rare APPOs, such as congenital anomalies and low Apgar scores. Furthermore, participants are of predominantly European ancestry, except for BiB. Enhancing the ancestry diversity in the data contributing to the collaboration is a key priority as outlined below in 'Collaborations and future plans'. Finally, despite our best efforts to derive accurate and standardised APPO definitions, there is inevitably a considerable degree of misclassification, especially where some studies derived their data only from self-reported information or only from hospital electronic health records.

Collaborations and future plans

We are currently focussing on systematically assessing the effects of predisposition to autoimmune, mental health, thyroid, reproductive, and cardiometabolic disorders on APPOs

For peer review only

(**Aim 2**), discovering new candidate drug targets for APPOs (**Aim 3**), and investigating the efficacy, safety and potential of repurposing existing drugs during pregnancy (**Aim 3**).

A future priority is to increase diversity in the genetic background of MR-PREG collaboration participants, given the current effort is predominantly focussed on participants of European ancestry. This will enable us to improve the internal validity of the MR-PREG studies (e.g., selecting better genetic instruments through using trans-ancestral data to improve statistical fine mapping) but also external validity (e.g., testing transportability of effects across ancestries). Additionally, we are incorporating more molecular data into MR-PREG studies to better understand the molecular mechanisms underlying APPOs, for example, placental transcriptomics and proteomics.

Finally, to enhance our work identifying molecular targets for the prevention and treatment of APPOs (**Aim 3**), we are utilizing exome and whole-genome sequencing data to pinpoint causal genes linked to these adverse outcomes.

We are advancing collaborations with additional large-scale studies including genetic and APPO data, with a focus on rarer outcomes such as congenital anomalies and participants of non-European ancestry to increase statistical power for key safety outcomes and ancestry diversity. Genetic association data for APPOs generated by the collaboration can only be used for research that is covered by data agreements with current contributing studies.

Data availability

The ALSPAC access policy that describes the proposal process in detail including any costs associated with conducting research at ALSPAC, which may be updated from time to time and is available at: https://www.bristol.ac.uk/medialibrary/sites/alspac/documents/researchers/dataaccess/ALSPAC_Access_Policy.pd

Data is available upon request from Born in Bradford: https://borninbradford.nhs.uk/research/how-to-access-data/

Data from MoBa are available from the Norwegian Institute of Public Health after application to the MoBa Scientific Management Group (see its

For peer review only - http://bmjopen.bmj.com/site/about/guidelines.xhtml

website https://www.fhi.no/en/op/data-access-from-health-registries-health-studies-and-biobanks/data-access/applying-for-access-to-data/ for details).

Researchers can apply for access to the UK Biobank data via the Access Management System (AMS) (https://www.ukbiobank.ac.uk/enable-your-research/apply-for-access).

Author contributions

The MR PREG collaboration was conceptualised by DAL and MCB. GC, NM, AGS, MCB, QY, TB, CC, EA, AT undertook data curation and generated GWAS pipelines for the individual cohorts. QY and MCB ran the meta-analysis. TB and MCB generated the WLM models. GC, NM, MCB, AGS and DAL, wrote, and all authors edited and approved the manuscript.

Acknowledgements

The authors would like to thank Professor Dave Evans, Dr Marwa Al-Arab, Dr Alba Fernandez-Sanles, Dr Alice Carter, Dr Helena Urquijo, Jevvy Huang, and Peiyuan Huang for their assistance with statistical analysis, GWAS, and technical support.

All cohort specific acknowledgements are detailed in the **Supplementary text**.

Funding declaration

All cohort specific funding information is detailed in the **Supplementary text**.

This publication is the work of the authors, who will serve as guarantors for the contents of this paper.

DAL, MCB, GC, AGS, TB, QY, AT, HC, EA, PH, and NM work in a unit supported by the University of Bristol and UK Medical Research Council (MC_UU_00032/5) and the British Heart Foundation (AA/18/1/34219). AGS is supported by the European Union's Horizon 2020 research and innovation programme (grant agreement No 874739, LongITools). AGS, GC and DAL are supported by STAGE that has received funding from the European Union's Horizon Europe Research and Innovation Programme under grant agreement nº 101137146. UK participants in Horizon Europe Project STAGE are supported by UKRI grant numbers 10112787 (Beta Technology), 10099041 (University of Bristol) and 10109957 (Imperial College London). EA is supported by a Wellcome Trust PhD studentship (228276/Z/23/Z). MCM is supported by the Research Council of Norway through its Centres of Excellence funding scheme (project No

For peer review only - http://bmjopen.bmj.com/site/about/guidelines.xhtml

262700) and the European Research Council under the European Union's Horizon 2020 research and innovation program (ERC Starting Grant, INFERTILITY grant agreement No 947684). JW was funded for BiB data collection by a UK Medical Research Council (MRC) Special Training Fellowship in Health of the Public and Health Services Research (MRC GO601712) and is now supported by supported by the UK Prevention Research Partnership (MR/S037527/1), an initiative funded by UK Research and Innovation Councils, the Department of Health and Social Care (England) and the UK devolved administrations, and leading health research charities.

Conflict of interest

All authors declare a no conflict of interest

For peer review only

For peer review only - http://bmjopen.bmj.com/site/about/guidelines.xhtml

References

1. Quenby S, Gallos ID, Dhillon-Smith RK, et al. Miscarriage matters: the epidemiological, physical, psychological, and economic costs of early pregnancy loss. *The Lancet* 2021;397(10285):1658-67. doi: 10.1016/S0140-6736(21)00682-6
2. Wang H, Li N, Chivese T, et al. IDF Diabetes Atlas: Estimation of Global and Regional Gestational Diabetes Mellitus Prevalence for 2021 by International Association of Diabetes in Pregnancy Study Group's Criteria. *Diabetes Res Clin Pract* 2022;183:109050. doi: 10.1016/j.diabres.2021.109050 [published Online First: 20211206]
3. Abalos E, Cuesta C, Grosso AL, et al. Global and regional estimates of preeclampsia and eclampsia: a systematic review. *Eur J Obstet Gynecol Reprod Biol* 2013;170(1):1-7. doi: 10.1016/j.ejogrb.2013.05.005 [published Online First: 20130607]
4. Ohuma EO, Moller AB, Bradley E, et al. National, regional, and global estimates of preterm birth in 2020, with trends from 2010: a systematic analysis. *Lancet* 2023;402(10409):1261-71. doi: 10.1016/s0140-6736(23)00878-4
5. Souza JP, Day LT, Rezende-Gomes AC, et al. A global analysis of the determinants of maternal health and transitions in maternal mortality. *The Lancet Global Health* 2024;12(2):e306-e16. doi: 10.1016/S2214-109X(23)00468-0
6. Geller SE, Koch AR, Garland CE, et al. A global view of severe maternal morbidity: moving beyond maternal mortality. *Reproductive Health* 2018;15(1):98. doi: 10.1186/s12978-018-0527-2
7. Catov JM, Margerison-Zilko C. Pregnancy as a window to future health: short-term costs and consequences. *Am J Obstet Gynecol* 2016;215(4):406-7. doi: 10.1016/j.ajog.2016.06.060
8. Rich-Edwards JW, Fraser A, Lawlor DA, et al. Pregnancy Characteristics and Women's Future Cardiovascular Health: An Underused Opportunity to Improve Women's Health? *Epidemiologic Reviews* 2014;36(1):57-70. doi: 10.1093/epirev/mxt006
9. Brito Nunes C, Borges MC, Freathy RM, et al. Understanding the Genetic Landscape of Gestational Diabetes: Insights into the Causes and Consequences of Elevated Glucose Levels in Pregnancy. *Metabolites* 2024;14(9) doi: 10.3390/metabo14090508 [published Online First: 20240920]
10. Wu P, Green M, Myers JE. Hypertensive disorders of pregnancy. *BMJ* 2023;381:e071653. doi: 10.1136/bmj-2022-071653
11. Angum F, Khan T, Kaler J, et al. The Prevalence of Autoimmune Disorders in Women: A Narrative Review. *Cureus* 2020;12(5):e8094. doi: 10.7759/cureus.8094 [published Online First: 20200513]
12. Singh M, Wambua S, Lee SI, et al. Autoimmune diseases and adverse pregnancy outcomes: an umbrella review. *BMC Medicine* 2024;22(1):94. doi: 10.1186/s12916-024-03309-y
13. Molenaar NM, Bais B, Lambregtse-van den Berg MP, et al. The international prevalence of antidepressant use before, during, and after pregnancy: A systematic review and meta-analysis of timing, type of prescriptions and geographical variability. *J Affect Disord* 2020;264:82-89. doi: 10.1016/j.jad.2019.12.014 [published Online First: 20191209]
14. Conrad N, Molenberghs G, Verbeke G, et al. Trends in cardiovascular disease incidence among 22 million people in the UK over 20 years: population based study. *Bmj*

For peer review only - http://bmjopen.bmj.com/site/about/guidelines.xhtml

2024;385:e078523. doi: 10.1136/bmj-2023-078523 [published Online First: 20240626]
15. Subramanian A, Azcoaga-Lorenzo A, Anand A, et al. Polypharmacy during pregnancy and associated risk factors: a retrospective analysis of 577 medication exposures among 1.5 million pregnancies in the UK, 2000-2019. *BMC Medicine* 2023;21(1):21. doi: 10.1186/s12916-022-02722-5
16. Sciences AoM. Understanding pregnancy: Accelerating the development of new therapies for pregnancy specific conditions. In: Foundation BHPatC, ed., 2023.
17. National Academies Sciences E, Medicine Consensus Study Report;. Advancing Clinical Research with Pregnant and Lactating Populations: Overcoming Real and Perceived Liability Risks, 2024.
18. UK Commission on Human Medicines. Report of the Commission on Human Medicines Expert Working Group on Optimising Data on Medicines used During Pregnancy, 2021.
19. Smith GD, Ebrahim S. 'Mendelian randomization': can genetic epidemiology contribute to understanding environmental determinants of disease? *Int J Epidemiol* 2003;32(1):1-22. doi: 10.1093/ije/dyg070
20. Lawlor DA, Harbord RM, Sterne JA, et al. Mendelian randomization: using genes as instruments for making causal inferences in epidemiology. *Stat Med* 2008;27(8):1133-63. doi: 10.1002/sim.3034
21. Zheng J, Baird D, Borges MC, et al. Recent Developments in Mendelian Randomization Studies. *Curr Epidemiol Rep* 2017;4(4):330-45. doi: 10.1007/s40471-017-0128-6 [published Online First: 20171122]
22. Yan M, Chen Z, Tang J, et al. Association between gestational diabetes mellitus and offspring health: a two-sample mendelian randomization study. *BMC Pregnancy and Childbirth* 2025;25(1):321. doi: 10.1186/s12884-025-07423-4
23. Taylor K, Wootton RE, Yang Q, et al. The effect of maternal BMI, smoking and alcohol on congenital heart diseases: a Mendelian randomisation study. *BMC Medicine* 2023;21(1):35. doi: 10.1186/s12916-023-02731-y
24. Tyrrell J, Richmond RC, Palmer TM, et al. Genetic Evidence for Causal Relationships Between Maternal Obesity-Related Traits and Birth Weight. (1538-3598 (Electronic))
25. Morris TT, Davies NM, Hemani G, et al. Population phenomena inflate genetic associations of complex social traits. *Science Advances* 2020;6(16):eaay0328. doi: 10.1126/sciadv.aay0328
26. Schmidt AF, Finan C, Gordillo-Marañón M, et al. Genetic drug target validation using Mendelian randomisation. *Nature Communications* 2020;11(1):3255. doi: 10.1038/s41467-020-16969-0
27. Holmes MV, Richardson TG, Ference BA, et al. Integrating genomics with biomarkers and therapeutic targets to invigorate cardiovascular drug development. *Nat Rev Cardiol* 2021;18(6):435-53. doi: 10.1038/s41569-020-00493-1 [published Online First: 20210311]
28. Zheng J, Haberland V, Baird D, et al. Phenome-wide Mendelian randomization mapping the influence of the plasma proteome on complex diseases. *Nature Genetics* 2020;52(10):1122-31. doi: 10.1038/s41588-020-0682-6
29. Atia A, Aboeldahab H, Wageeh A, et al. Safety and Efficacy of Proprotein Convertase Subtilisin-Kexin Type 9 Inhibitors After Acute Coronary Syndrome: A Meta-analysis of Randomized Controlled Trials. *American Journal of Cardiovascular Drugs* 2024;24(1):83-102. doi: 10.1007/s40256-023-00621-5

For peer review only - http://bmjopen.bmj.com/site/about/guidelines.xhtml

30. Lawlor DA, Tilling K, Davey Smith G. Triangulation in aetiological epidemiology. *Int J Epidemiol* 2016;45(6):1866-86. doi: 10.1093/ije/dyw314
31. VanderWeele TJ. Outcome-wide Epidemiology. *Epidemiology* 2017;28(3):399-402. doi: 10.1097/ede.0000000000000641
32. Boyd A, Golding J, Macleod J, et al. Cohort Profile: the 'children of the 90s' – the index offspring of the Avon Longitudinal Study of Parents and Children. *Int J Epidemiol* 2013;42 doi: 10.1093/ije/dys064
33. Fraser A, Macdonald-Wallis C, Tilling K, et al. Cohort profile: The Avon Longitudinal Study of Parents and Children: ALSPAC mothers cohort. *Int J Epidemiol* 2013;42 doi: 10.1093/ije/dys066
34. Wright J, Small N, Raynor P, et al. Cohort Profile: the Born in Bradford multi-ethnic family cohort study. *Int J Epidemiol* 2013;42(4):978-91. doi: 10.1093/ije/dys112 [published Online First: 20121012]
35. Littlejohns TJ, Sudlow C, Allen NE, et al. UK Biobank: opportunities for cardiovascular research. *Eur Heart J* 2019;40(14):1158-66. doi: 10.1093/eurheartj/ehx254
36. Sudlow C, Gallacher J, Allen N, et al. UK biobank: an open access resource for identifying the causes of a wide range of complex diseases of middle and old age. *PLoS Med* 2015;12(3):e1001779. doi: 10.1371/journal.pmed.1001779 [published Online First: 20150331]
37. UK Biobank. UK Biobank hospital inpatient data, 2023.
38. Pervjakova N, Moen GA-O, Borges MC, et al. Multi-ancestry genome-wide association study of gestational diabetes mellitus highlights genetic links with type 2 diabetes. (1460-2083 (Electronic))
39. Steinthorsdottir V, McGinnis R, Williams NO, et al. Genetic predisposition to hypertension is associated with preeclampsia in European and Central Asian women. *Nat Commun* 2020;11(1):5976. doi: 10.1038/s41467-020-19733-6 [published Online First: 20201125]
40. Solé-Navais P, Flatley C, Steinthorsdottir V, et al. Genetic effects on the timing of parturition and links to fetal birth weight. *Nat Genet* 2023;55(4):559-67. doi: 10.1038/s41588-023-01343-9 [published Online First: 20230403]
41. Guintivano J, Byrne EM, Kiewa J, et al. Meta-Analyses of Genome-Wide Association Studies for Postpartum Depression. *Am J Psychiatry* 2023;180(12):884-95. doi: 10.1176/appi.ajp.20230053 [published Online First: 20231018]
42. Willer CJ, Li Y, Abecasis GR. METAL: fast and efficient meta-analysis of genomewide association scans. *Bioinformatics* 2010;26(17):2190-1. doi: 10.1093/bioinformatics/btq340 [published Online First: 20100708]
43. Ani A, van der Most PJ, Snieder H, et al. GWASinspector: comprehensive quality control of genome-wide association study results. *Bioinformatics* 2021;37(1):129-30. doi: 10.1093/bioinformatics/btaa1084
44. Wu Y, Zhong X, Lin Y, et al. Estimating genetic nurture with summary statistics of multigenerational genome-wide association studies. *Proceedings of the National Academy of Sciences* 2021;118(25):e2023184118. doi: doi:10.1073/pnas.2023184118
45. Yang Q, Magnus MC, Kilpi F, et al. Evaluating causal associations of chronotype with pregnancy and perinatal outcomes and its interactions with insomnia and sleep duration: a Mendelian randomization study. *medRxiv* 2023 doi: 10.1101/2023.06.02.23290898 [published Online First: 20230605]

For peer review only - http://bmjopen.bmj.com/site/about/guidelines.xhtml

46. Zhao J, Stewart ID, Baird D, et al. Causal effects of maternal circulating amino acids on offspring birthweight: a Mendelian randomisation study. (2352-3964 (Electronic))
47. Barry CA-O, Lawlor DA, Shapland CA-O, et al. Using Mendelian Randomisation to Prioritise Candidate Maternal Metabolic Traits Influencing Offspring Birthweight. LID - 10.3390/metabo12060537 [doi] LID - 537. (2218-1989 (Print))
48. Yang Q, Magnus MC, Kilpi F, et al. Investigating causal relations between sleep duration and risks of adverse pregnancy and perinatal outcomes: linear and nonlinear Mendelian randomization analyses. *BMC Medicine* 2022;20(1):295. doi: 10.1186/s12916-022-02494-y
49. Borges MC, Clayton GL, Freathy RM, et al. Integrating multiple lines of evidence to assess the effects of maternal BMI on pregnancy and perinatal outcomes. *BMC Medicine* 2024;22(1):32. doi: 10.1186/s12916-023-03167-0
50. Taylor K, McBride N, Zhao J, et al. The Relationship of Maternal Gestational Mass Spectrometry-Derived Metabolites with Offspring Congenital Heart Disease: Results from Multivariable and Mendelian Randomization Analyses. *J Cardiovasc Dev Dis* 2022;9(8) doi: 10.3390/jcdd9080237 [published Online First: 20220727]
51. Yang Q, Borges MC, Sanderson E, et al. Associations between insomnia and pregnancy and perinatal outcomes: Evidence from mendelian randomization and multivariable regression analyses. *PLOS Medicine* 2022;19(9):e1004090. doi: 10.1371/journal.pmed.1004090
52. Yang Q, Sanderson E, Tilling K, et al. Exploring and mitigating potential bias when genetic instrumental variables are associated with multiple non-exposure traits in Mendelian randomization. *Eur J Epidemiol* 2022;37(7):683-700. doi: 10.1007/s10654-022-00874-5 [published Online First: 20220527]
53. McBride N, Fernández-Sanlés A, Al Arab M, et al. Effects of the maternal and fetal proteome on birth weight: a Mendelian randomization analysis. *medRxiv* 2025 doi: 10.1101/2023.10.20.23297135 [published Online First: 20250120]
54. Brand JS, Gaillard R, West J, et al. Associations of maternal quitting, reducing, and continuing smoking during pregnancy with longitudinal fetal growth: Findings from Mendelian randomization and parental negative control studies. *PLOS Medicine* 2019;16(11):e1002972. doi: 10.1371/journal.pmed.1002972

For peer review only - http://bmjopen.bmj.com/site/about/guidelines.xhtml

The Mendelian Randomization in Pregnancy (MR-PREG) collaboration: Improving evidence for prevention and treatment of adverse pregnancy and perinatal outcomes

Contents

Genotyping, imputation and quality control

Genome-wide association analyses

Weighted linear model (WLM)

Ethical approval

Acknowledgments

Funding

For peer review only

For peer review only - http://bmjopen.bmj.com/site/about/guidelines.xhtml

Genotyping, imputation and quality control

Specific genotyping, imputation and quality control (QC) procedures were applied within each cohort and are described below.

Avon Longitudinal Study of Parents and Children (ALSPAC)

Avon Longitudinal Study of Parents and Children (ALSPAC) children (1) were genotyped using the Illumina HumanHap550 quad chip genotyping platforms by 23andMe subcontracting the Wellcome Trust Sanger Institute, Cambridge, UK and the Laboratory Corporation of America, Burlington, NC, US. The resulting raw genome-wide data were subjected to standard QC methods. Individuals were excluded based on gender mismatches; minimal or excessive heterozygosity; disproportionate levels of individual missingness (>3%) and insufficient sample replication (IBD < 0.8). Population stratification was assessed by multidimensional scaling analysis and compared with Hapmap II (release 22) European descent (CEU), Han Chinese, Japanese and Yoruba reference populations; all individuals with non-European ancestry were removed. SNPs with a minor allele frequency of < 1%, a call rate of < 95% or evidence for violations of Hardy-Weinberg equilibrium (P < 5E-7) were removed. Cryptic relatedness was measured as proportion of identity by descent (IBD > 0.1). Related subjects that passed all other QC thresholds were retained during subsequent phasing and imputation. 9,115 subjects and 500,527 SNPs passed these QC filters.

ALSPAC mothers (2) were genotyped using the Illumina human660W-quad array at Centre National de Génotypage (CNG) and genotypes were called with Illumina GenomeStudio. PLINK (v1.07) was used to carry out QC measures on an initial set of 10,015 subjects and 557,124 directly genotyped SNPs. SNPs were removed if they displayed more than 5% missingness or a Hardy-Weinberg equilibrium P value of less than 1.0e-06. Additionally SNPs with a minor allele frequency of less than 1% were removed. Samples were excluded if they displayed more than 5% missingness, had indeterminate X chromosome heterozygosity or extreme autosomal heterozygosity. Samples showing evidence of population stratification were identified by multidimensional scaling of genome-wide identity by state pairwise distances using the four HapMap populations as a reference, and then excluded. Cryptic relatedness was assessed using a IBD estimate of more than 0.125 which is expected to correspond to roughly 12.5% alleles shared IBD or a relatedness at the first cousin level. Related subjects that passed all other QC thresholds were retained during subsequent phasing and imputation. 9,048 subjects and 526,688 SNPs passed these QC filters.

SNP genotypes in common between the sample of mothers and sample of children were combined (N = 477,482). SNPs with genotype missingness above 1% (N = 11,396 SNPs) or individuals with potential ID mismatches (N = 321 individuals) were removed. This resulted in a dataset of 17,842 subjects containing 6,305 duos and 465,740 SNPs (112 were removed during liftover and 234 were out of HWE after combination).

Haplotypes were estimated using ShapeIT (v2.r644) which utilises relatedness during phasing. The phased haplotypes were then imputed to the Haplotype Reference Consortium (HRCr1.1, 2016) panel of approximately 31,000 phased whole genomes. The HRC panel was phased using ShapeIt v2, and the imputation was performed using the Michigan imputation server. This resulted in 8,237 eligible children and 8,196 eligible mothers with available genotype data after exclusion of related subjects using cryptic

For peer review only - http://bmjopen.bmj.com/site/about/guidelines.xhtml

relatedness measures described previously. Reference genome build version 37 (GRCh37/hg19) was used for genomic positions for ALSPAC genotype data.

Born in Bradford (BiB)

Born in Bradford (BiB) (3) mothers and offspring were genotyped at Bristol Bioresource Laboratories, Bristol, UK using four different Illumina arrays: HumanCoreExome12v1.0, HumanCoreExome12v1.1, HumanCoreExome24v1.0 and Infinium Global Screening Array-24 v1.0 (GSA). Genotypes were called with Illumina GenomeStudio. For each of the four subsets of participants genotyped on each array, any individual or SNP with >3% missing genotype calls was dropped and the four subsets were merged, giving a total merged sample of 20,000 individuals. It was not appropriate to apply filters for deviation from Hardy-Weinberg Equilibrium and excess homozygosity to this merged dataset, given the population structure and consanguinity known to be present. 707 individuals were then dropped due to i) mismatch between genetically- and phenotypically-defined sex (110 individuals), ii) suspected sample duplication (127 individuals), and iii) discrepancy between expected and genetically inferred relationship for first degree relatives (470 individuals). Principal component analysis (PCA) was carried out and involved assigning participants to two groups that were genetically similar to the European or South Asian HapMap samples. Ancestry data were also self-reported or obtained from primary care medical records, and categorised using the UK Office of National Statistics criteria: (1) White European ('White British' or 'White European'), (2) South Asian ('Pakistani', 'Indian' or 'Bangladeshi'), (3) Caribbean or African ('Afro-Caribbean' or 'African') or (4) others. Individuals whose PCA-based ancestry agreed with their self-reported/medical record-derived ancestry were assigned to two groups, for which we use the shorthand "White European" (WE) and "South Asian" (SA). The SA group consisted primarily of individuals with Pakistani self-reported/medical record ancestry, and does not include Bangladeshis but does include some Indians.

Genotype imputation was performed for 7606 WE and 8692 SA participants, with each ancestry and array (CoreExome/GSA) imputed separately. Prior to imputation, variants with call rate <95%, HWE P-value <1e-6 or MAF <1% were removed for each of the four ancestry/array groups separately. A/T and C/G SNPs were dropped, as were those that failed checks for strand, reference/alternative alleles, position, and SNP duplication, indels, non-autosomal SNPs, SNPs with no match in the HRC panel (position or SNP ID), and SNPs with non-matching alleles. Genotype data were imputed to the HRC r1.1 reference panel. Reference genome build version 37 (GRCh37/hg19) was used for genomic positions for BiB genotype data.

The Norwegian Mother Father and Child Cohort Study (MoBa)

In The Norwegian Mother Father and Child Cohort Study (MoBa) (4), genotyping of the samples was performed in seven different batches on different Illumina platforms over a period of four years at deCODE genetics, Reykjavik, Iceland (HumanCoreExome-12 v.1.1, HumanCoreExome-24 v.1.0, Global Screening Array v.1.0, HumanOmniExpress-24-v1.0, InfiniumOmniExpress-24v1.2, and GSA24-v1.0). Reference genome build version 37 (GRCh37/hg19) was used for genomic positions for MoBa genotype data. Genotypes were called in Illumina GenomeStudio v.2011.1 for the HARVEST substudy (11,490 triads) and v.2.0.3 for the remaining batches. Cluster positions were identified from samples with call rate ≥ 0.98 and GenCall score ≥ 0.15.

For peer review only - http://bmjopen.bmj.com/site/about/guidelines.xhtml

Pre-imputation QC was performed for each subpopulation on the SNP, individual, and family level. Subpopulations were defined using principal component (PC) analyses using 1000 Genomes phase 1 data to identify the European, Asian, and African core subpopulations (after removing SNPs with MAF < 1%, call rate < 95%, and HWE p-value < 0.001). The primary software used for the QC were PLINK 1.9 and KING 2.2.5. For the SNP-level QC, SNPs were removed if MAF < 0.5%, call rate < 95%, HWE p-value < 0.000001, discordant in duplicate pairs, associated with genotype plate and genotype batch at p-value 0.001. For individual-level QC, individuals were removed if heterozygosity outliers Fhet ± 0.2, erroneous sex assignment, known relatedness, cryptic relatedness, identity-by-decent (PI_HAT threshold of 0.15), and PC outliers both with and without 1000 Genomes. For family-level QC, families with more than 5% Mendel errors and SNPs with more than 1% of Mendel errors were removed, while other minor Mendel errors were zeroed out. Batches that were genotyped using the same array were merged (keeping only SNPs present in all batches) and the pre-imputation QC was performed on the merged batches.

Phasing and imputation were performed using the publicly available Haplotype Reference Consortium data. Phasing was performed using SHAPEIT2 with the duoHMM algorithm to incorporate the pedigree information into the haplotype estimates. IMPUTE 4 was then used to perform imputation. Dosage data was converted to best-guess (hard call) genotype data using certainty threshold 0.7, and variants with imputation quality score (INFO) <0.8 were dropped.

Post-imputation QC was then performed as for the steps outlined for pre-imputation QC, with the following changes: i) MAF filter 1%, ii) call rate <95% for SNPs, and iii) P-value for batch effects filter <5e-8. To ensure that across batch relatedness (both known and unknown) was accounted for in all analyses the three imputation batches were then merged, and the post-imputation QC steps were repeated, with the exception that a filter was applied for imputation batch effects rather than genotype batch effects.

UK Biobank

A full description of the genetic QC performed by the MRC-Integrative Epidemiology Unit on UK Biobank data is available elsewhere (5). The full data release contains the cohort of successfully genotyped samples (n=488,377). 49,979 individuals were genotyped using the UK BiLEVE array and 438,398 using the UK Biobank axiom array. Pre-imputation QC, phasing and imputation are described elsewhere (6). In brief, prior to phasing, multiallelic SNPs or those with MAF ≤1% were removed. Phasing of genotype data was performed using a modified version of the SHAPEIT2 algorithm (7). Genotype imputation to a reference set combining the UK10K haplotype and HRC reference panels (8) was performed using IMPUTE2 algorithms (9). The analyses presented here were restricted to autosomal variants using a graded filtering with varying imputation quality for different allele frequency ranges. Therefore, rarer genetic variants are required to have a higher imputation INFO score (Info>0.3 for MAF >3%; Info>0.6 for MAF 1-3%; Info>0.8 for MAF 0.5-1%; Info>0.9 for MAF 0.1-0.5%) with MAF and Info scores having been recalculated on an in-house derived 'European' subset.

In post-imputation QC, individuals with sex-mismatch (derived by comparing genetic sex and reported sex) or individuals with sex chromosome aneuploidy were excluded from the analysis (n=814). The sample was restricted to individuals of 'European' ancestry as defined by an in-house k-means cluster analysis performed using the first 4 principal components provided by UK Biobank in the statistical software environment R. The current analysis includes the largest cluster from this analysis (n=464,708). Estimated

For peer review only - http://bmjopen.bmj.com/site/about/guidelines.xhtml

kinship coefficients using the KING toolset (10) identified 107,162 pairs of related individuals. An in-house algorithm was then applied to this list and preferentially removed the individuals related to the greatest number of other individuals until no related pairs remain. These individuals were excluded (n=79,448). Additionally, 2 individuals were removed due to them relating to a very large number (>200) of individuals. Reference genome build version 37 (GRCh37/hg19) was used for genomic positions for UKB genotype data.

FinnGen

A full description of the FinnGen study and the QC procedures applied to the genetic data is available elsewhere (11, 12). We used release 12 of the publicly available FinnGen summary statistics. FinnGen individuals were genotyped with Illumina and Affymetrix chip arrays (Illumina Inc., San Diego, and Thermo Fisher Scientific, Santa Clara, CA, USA). Genotype calls were made with GenCall and zCall algorithms for Illumina and AxiomGT1 algorithm for Affymetrix data. Chip genotyping data produced with previous chip platforms and reference genome builds were lifted over to build version 38 (GRCh38/hg38). In sample-wise QC steps, individuals with ambiguous gender, high genotype missingness (>5%), excess heterozygosity (+-4SD) and non-Finnish ancestry were excluded. In variant-wise QC steps, variants with high missingness (>2%), low HWE P-value (<1e-6) and low minor allele count (MAC<3) were excluded. Before imputation, chip-genotyped samples were pre-phased with Eagle 2.3.5 using the default parameters, except the number of conditioning haplotypes, which was set to 20,000. Chip genotype data were imputed using the population-specific SISu v4.2 imputation reference panel of 8,554 whole genomes with Beagle 4.1 (version 27Jan18.7e1). Post-imputation QC involved checking the expected conformity of the imputation INFO-value distribution, MAF differences between the target dataset and the imputation reference panel and checking chromosomal continuity of the imputed genotype calls. Merged imputed genotype data is composed of 141 data sets and includes samples from multiple cohorts, yielding a total sample size of 520,210 participants with genotype data for 21,311,644 variants.

The FinnGen data for 180,042 pruned SNPs was merged with the 1k genome project (1kgp) data, and two rounds of PCA were carried out. After kinship analysis, the sample was separated into three groups: 296,829 inliers (unrelated participants with Finnish ancestry), 203,908 outliers (non-duplicate samples with Finnish ancestry but who are also related to the inliers), and 19,473 rejected participants who had non-Finnish ancestry, or who were twins/duplicates with relations to other (non-inlier) samples. A final round of PCA for the inliers was calculated, after which outliers were projected onto the same PC space, yielding PC covariates for a total of 500,737 participants. After dropping 355 participants due to missing minimum phenotype data, and dropping 34 participants due to sex check failure, 500,348 participants were available for genome-wide association study (GWAS) analyses, of which 282,064 were female. Reference genome build version 38 (GRCh38/hg38) was used for genomic positions for FinnGen.

Genome-wide association analyses

Genome-wide association study (GWAS) analyses were run separately for mothers, fathers and offspring (when sufficient data were available) within each cohort, using software and approaches which were tailored to optimally account for the population stratification and relatedness present in each sample.

For peer review only - http://bmjopen.bmj.com/site/about/guidelines.xhtml

ALSPAC

We performed GWAS analysis in ALSPAC mothers and offspring using PLINK 2.0 alpha (build 22 Dec 2024) (13, 14). The analytical samples were restricted to unrelated mothers (IBD ≤ 0.125) and offspring (IBD ≤ 0.1). Association analyses were performed with imputed autosomal genotype data, for adverse pregnancy and perinatal outcomes (APPOs) via the "--glm" command using Firth logistic regression for binary outcomes and linear regression for continuous outcomes. Firth regression enables less biased effect size estimation in the presence of case-control imbalance for binary outcomes. We assumed an additive model and adjusted for the top 10 principal components of ancestry (PCs). PCs were generated using Plink –pca option after removing related individuals based on a set of independent SNPs excluding any long-range LD regions (variant count window size = 100, variant count to shift the window = 5, variance inflation factor (VIF) threshold = 1.01).

BiB

We performed GWAS analysis in BiB mothers and offspring using REGENIE version 3.6 (15) to account for the complex population structure (population stratification and relatedness) that is present in BiB, via a whole-genome regression approach. REGENIE implements whole-genome regression in two steps: in step 1, array genotypes are used to generate 22 leave-one-chromosome-out (LOCO) predictions for each outcome. In step 2, during which the imputed genotype associations with each outcome are estimated, these LOCO predictions are then used as covariates. We performed GWAS separately for the two largest ancestry groups present in BiB as described above (SA and WE). We did not use REGENIE's automatic phenotype imputation feature for missing APPOs and instead ran REGENIE for each outcome separately. For step 1 we used the called genotype data obtained by merging the samples genotyped on different arrays (as described above), after which the PLINK 2.0 filters "--maf 0.01", "--hwe 0.000001" and "--geno 0.03" were applied, yielding sets of 99,131 and 135,801 variants for the SA and WE samples respectively. For step 1 we set genotype block size ("--bsize") to 1000. For step 2, we ran association analyses with imputed autosomal genotype data, having first merged the samples genotyped on the CoreExome and GSA arrays and subsetted variants to those with imputation accuracy R^2 ≥0.3 when imputed from both arrays. We set genotype block size to 200, applied a minimum minor allele count (MAC) filter of 20, and used linear regression or Firth logistic regression (via REGENIE options "--firth --approx --pThresh 0.999999") for continuous and binary outcomes respectively. We computed the standard error based on the Firth regression effect size and likelihood ratio test *P*-value as per the "--firth-se" option. Models were adjusted for genotyping array and 40 PCs: 20 internal PCs (calculated using only BiB data via the flashpca package version 2.0 (16) with the authors' recommended settings (16, 17)) and 20 external PCs (BiB participants were projected onto a UK Biobank PC space via the pcapred R package (18).

MoBa

We performed GWAS analysis in MoBa mothers, fathers and offspring using whole genome regression implemented in REGENIE version 3.1.2 (15). We did not use REGENIE's automatic phenotype imputation feature for missing APPOs and instead ran REGENIE for each outcome separately. We performed step 1 using 455,827 imputed genetic variants which were directly genotyped in ≥1 imputation batch and had imputation quality INFO score >0.99785, having first removed 35,810 variants in the major histocompatibility complex (MHC) region on chromosome 6 (variants with MAF <1% had already been removed in pre-phase QC). We set genotype block size ("--bsize") to 1000. For step 2, we ran association analyses with 6,981,748 imputed (hard called) autosomal genotype data, adjusting for genotyping batch

For peer review only - http://bmjopen.bmj.com/site/about/guidelines.xhtml

and 20 PCs. We set genotype block size to 200 and used linear regression or Firth logistic regression (via REGENIE options "--firth --approx --pThresh 0.999999") for continuous and binary outcomes respectively. We computed the standard error based on the Firth regression effect size and likelihood ratio test *P*-value as per the "--firth-se" option.

UK BioBank

We performed GWAS analysis in UK Biobank mothers only, using whole genome regression implemented in REGENIE version v3.6. (15). We did not use REGENIE's automatic phenotype imputation feature for missing APPOs and instead ran REGENIE for each outcome separately. We performed step 1 using 549,043 directly genotyped variants with MAF ≥0.01, variant call rate ≥0.9; Hardy-Weinberg equilibrium P >1.0 x 10^{-15} and MAC ≥100, and set genotype block size ("--bsize") to 1000. For step 2, we ran association analyses with imputed genotype data for the APPOs, adjusting for genotyping array and 40 PCs. We set genotype block size to 200 and used linear regression or Firth logistic regression (via REGENIE options "--firth --approx --pThresh 0.999999") for continuous and binary outcomes respectively. We computed the standard error based on the Firth regression effect size and likelihood ratio test *P*-value as per the "--firth-se" option.

FinnGen

GWAS analyses were caried out in FinnGen mothers only, using whole -genome regression implemented in REGENIE version 2.2.4 (15). For REGENIE step 1, 215,152 pruned (1.5Mb window and r2 threshold of 0.2) variants were used after applying the following filters: i) imputation INFO score >0.95 in all batches, ii) >97 % non-missing genotype, and iii) MAF >1 %, and a genotype block size of 1000 was used. For REGENIE step 2, association analyses with imputed genotype data for APPOs were conducted for each variant with a minimum allele count of 5 among each phenotype's cases and controls. The approximate Firth test was used for variants with an initial *P*-value of less than 0.01 and the standard error was computed based on the Firth regression effect size and likelihood ratio test *P*-value (REGENIE options --firth --approx --pThresh 0.01 --firth-se).

Post-GWAS quality control

Prior to meta-analysis we excluded variants with low MAF (<0.01) or low INFO score (<0.4) from the summary statistics from individual cohorts and maternal/paternal/offspring samples. We applied a standardised QC pipeline to GWAS summary statistics from individual cohorts and from the meta-analysis using the GWASInspector R package (19). This QC process included comparing the allele frequencies (AF) in the GWAS summary statistics to those in suitable reference panels. In the FinnGen summary statistics a small subset of variants had the opposite AF to the HRC reference panel (i.e. $AF_{FinnGen} = 1 - AF_{HRC}$). We therefore dropped 4064 variants which had a FinnGen versus HRC AF difference >0.2 from the meta-analyses.

Weighted linear model (WLM)

We carried out marginal (unadjusted) GWAS as described above, in which outcomes were regressed separately on maternal and offspring genotype, without mutual adjustment of the effects of maternal and offspring genotypes. After meta-analysis of the marginal GWAS effects we applied a weighted linear

For peer review only - http://bmjopen.bmj.com/site/about/guidelines.xhtml

model (WLM) (21-23) implemented in the DONUTS R package (24) to estimate conditional (adjusted) maternal ($\hat{\beta}_m$) and offspring ($\hat{\beta}_o$) genetic effects (the mutually adjusted coefficients for maternal and offspring genotype, fitted jointly in the same model), as linear combinations of the estimated marginal offspring ($\hat{b}_o$) and maternal ($\hat{b}_m$) genetic effects:

$$\hat{\beta}_o = \frac{4}{3}\hat{b}_o - \frac{2}{3}\hat{b}_m$$

$$\hat{\beta}_m = \frac{4}{3}\hat{b}_m - \frac{2}{3}\hat{b}_o$$

$$\mathrm{SE}(\hat{\beta}_o) = \sqrt{\frac{16}{9}\mathrm{var}(\hat{b}_o) + \frac{4}{9}\mathrm{var}(\hat{b}_m) - \frac{16}{9} \times \widehat{\mathrm{int}}_{o,m} \times \mathrm{SE}(\hat{b}_m)\mathrm{SE}(\hat{b}_o)}$$

$$\mathrm{SE}(\hat{\beta}_m) = \sqrt{\frac{16}{9}\mathrm{var}(\hat{b}_m) + \frac{4}{9}\mathrm{var}(\hat{b}_o) - \frac{16}{9} \times \widehat{\mathrm{int}}_{o,m} \times \mathrm{SE}(\hat{b}_m)\mathrm{SE}(\hat{b}_o)}$$

where $\widehat{\mathrm{int}}_{o,m}$ is the intercept term from bivariate LD score regression (25) of the maternal and offspring marginal summary statistics. These intercept terms are necessary to account for sample overlap between the marginal GWAS samples, and were estimated using Hapmap 3 SNPs with MAF >1%, excluding SNPs in the MHC region and with association Chi square statistics >80. We refer to the above model as the duos WLM, and it assumes that conditional paternal effects are zero.

Paternal genotype data were available in MoBa. We therefore also conducted marginal paternal GWAS, enabling us to relax the assumption that conditional paternal effects are zero by estimating the conditional maternal, offspring and paternal ($\hat{\beta}_p$) genetic effects via a trios WLM:

$$\hat{\beta}_o = 2\hat{b}_o - \hat{b}_m - \hat{b}_p$$

$$\hat{\beta}_m = \frac{3}{2}\hat{b}_m - \hat{b}_o + \frac{1}{2}\hat{b}_p$$

$$\hat{\beta}_p = \frac{3}{2}\hat{b}_p - \hat{b}_o + \frac{1}{2}\hat{b}_m$$

with their standard errors:

$$\mathrm{SE}(\hat{\beta}_o) = \sqrt{4\mathrm{var}(\hat{b}_o) + \mathrm{var}(\hat{b}_m) + \mathrm{var}(\hat{b}_p) + 2 \times \mathrm{int}_{m,p} \times \mathrm{SE}(\hat{b}_m)\mathrm{SE}(\hat{b}_p) - 4 \times \mathrm{int}_{o,m} \times \mathrm{SE}(\hat{b}_o)\mathrm{SE}(\hat{b}_m) - 4 \times \mathrm{int}_{o,p} \times \mathrm{SE}(\hat{b}_o)\mathrm{SE}(\hat{b}_p)}$$

$$\mathrm{SE}(\hat{\beta}_m) = \sqrt{\frac{9}{4}\mathrm{var}(\hat{b}_m) + \mathrm{var}(\hat{b}_o) + \frac{1}{4}\mathrm{var}(\hat{b}_p) - \mathrm{int}_{o,p} \times \mathrm{SE}(\hat{b}_o)\mathrm{SE}(\hat{b}_p) - 3 \times \mathrm{int}_{o,m} \times \mathrm{SE}(\hat{b}_o)\mathrm{SE}(\hat{b}_m) + \frac{3}{2} \times \mathrm{int}_{m,p} \times \mathrm{SE}(\hat{b}_m)\mathrm{SE}(\hat{b}_p)}$$

For peer review only - http://bmjopen.bmj.com/site/about/guidelines.xhtml

$$\mathrm{SE}(\hat{\beta}_p) = \sqrt{\frac{9}{4}\mathrm{var}\left(\hat{b}_p\right) + \mathrm{var}(\hat{b}_o) + \frac{1}{4}\mathrm{var}\left(\hat{b}_m\right) - \mathrm{int}_{o,m} \times \mathrm{SE}\left(\hat{b}_o\right)\mathrm{SE}\left(\hat{b}_m\right) - 3 \times \mathrm{int}_{o,p} \times \mathrm{SE}\left(\hat{b}_o\right)\mathrm{SE}\left(\hat{b}_p\right) + \frac{3}{2} \times \mathrm{int}_{m,p} \times \mathrm{SE}\left(\hat{b}_m\right)\mathrm{SE}\left(\hat{b}_p\right)},$$

where $\mathrm{int}_{o,m}$, $\mathrm{int}_{o,p}$, and $\mathrm{int}_{m,p}$ are the intercepts from bivariate LD score regression of the maternal and offspring, paternal and offspring and maternal and paternal marginal summary statistics respectively, and $\hat{b}_p$ is the estimated marginal paternal genetic effect.

The DONUTS software package extends the duos and trios WLMs to account for assortative mating via a parameter α. We set α to zero (i.e. we assumed mating was random) because previous sensitivity analyses in MoBa suggested this gave the most accurate estimates for conditional effects on birth weight [Bond et al medrXiv].

Ethical approval

ALSPAC

Ethical approval for the study was obtained from the ALSPAC Ethics and Law Committee and the Local Research Ethics Committees. Consent for biological samples has been collected in accordance with the Human Tissue Act (2004).

Ethical approval was obtained from the ALSPAC Ethics and Law Committee and the Local Research Ethics Committees. Consent for biological samples has been collected in accordance with the Human Tissue Act (2004). Informed consent for the use of data collected via questionnaires and clinics was obtained from participants following the recommendations of the ALSPAC Ethics and Law Committee at the time (details and reference numbers of all ethics approvals can be found at http://www.bristol.ac.uk/media-library/sites/alspac/documents/governance/Research%20Ethics%20Committee%20approval%20references.pdf).

BiB

Ethical approval for the study was granted by the Bradford National Health Service Research Ethics Committee (ref 06/Q1202/48), and all participants gave written informed consent. The ALL IN sub-study had ethical approval from the London School of Hygiene & Tropical Medicine ethics committee (ref: 5320) and the Bradford Research Ethics committee (ref: 08/H1302/21). Parents (usually the mother) gave informed, written consent to take part in the study.

MoBa

The current study is based on version 12 of the quality-assured data files released for research in 2019. The establishment of MoBa and initial data collection was based on a license from the Norwegian Data Protection Agency and approval from The Regional Committees for Medical and Health Research Ethics. The MoBa cohort is currently regulated by the Norwegian Health Registry Act. The current study was approved by The Regional Committees for Medical and Health Research Ethics of South/East Norway (ref 2018/1256).

For peer review only - http://bmjopen.bmj.com/site/about/guidelines.xhtml

UK Biobank

The UK Biobank has approval from the North West Multi-centre Research Ethics Committee (MREC) as a Research Tissue Bank (RTB) approval. This RTB approval was granted initially in 2011 (11/NW/0382) and it is renewed on a 5-yearly cycle, with the latest one successfully renewed in 2021 (21/NW/0157).

Acknowledgments

ALSPAC

We are extremely grateful to all the families who took part in this study, the midwives for their help in recruiting them, and the whole ALSPAC team, which includes interviewers, computer and laboratory technicians, clerical workers, research scientists, volunteers, managers, receptionists and nurses.

BiB

BiB is only possible because of the enthusiasm and commitment of the Children and Parents in BiB. We are grateful to all the participants, teachers, school staff, health professionals and researchers who have made BiB happen.

MoBa

This research has been conducted using MoBa data using application number 2552. MoBa is supported by the Norwegian Ministry of Health and Care services and the Ministry of Education and Research. We are grateful to all the participating families in Norway who take part in this on-going cohort study. We thank the Norwegian Institute of Public Health (NIPH) for generating high-quality genomic data. This research is part of the HARVEST collaboration, supported by the Research Council of Norway (#229624). We also thank the NORMENT Centre for providing genotype data, funded by the Research Council of Norway (#223273), South East Norway Health Authority and KG Jebsen Stiftelsen. We further thank the Center for Diabetes Research, the University of Bergen for providing genotype data and performing QC and imputation of the data funded by the ERC AdG project SELECTionPREDISPOSED, Stiftelsen Kristian Gerhard Jebsen, Trond Mohn Foundation, the Research Council of Norway, the Novo Nordisk Foundation, the University of Bergen, and the Western Norway health Authorities (Helse Vest).

FinnGen

The authors thank the FinnGen investigators for sharing their summary-level data.

UK Biobank

We would like to thank all the participants of UK Biobank for their vital contribution to the resource. This research has been conducted using the UK Biobank Resource under Application Number 23938**.**

For peer review only - http://bmjopen.bmj.com/site/about/guidelines.xhtml

Funding

ALSPAC

The UK Medical Research Council and Wellcome (Grant ref: 217065/Z/19/Z) and the University of Bristol provide core support for ALSPAC.A comprehensive list of grants funding is available on the ALSPAC website (http://www.bristol.ac.uk/alspac/external/documents/grant-acknowledgements.pdf). ALSPAC GWAS data was generated by Sample Logistics and Genotyping Facilities at Wellcome Sanger Institute and LabCorp (Laboratory Corporation of America) using support from 23andMe. This research was funded in part by the Wellcome Trust (Grant ref: 217065/Z/19/Z)]. For the purpose of Open Access, the author has applied a CC BY public copyright licence to any Author Accepted Manuscript version arising from this submission.

MoBa

MoBa funding is under **Acknowledgements** as requested by MoBa publication guidelines.

BiB

BiB receives core funding from the Wellcome Trust (WT101597MA), a joint grant from the UK Medical and Economic and Social Science Research Councils (MR/N024397/1), British Heart Foundation (CS/16/4/32482), and the National Institute of Health Research under its Applied Research Collaboration for Yorkshire and Humber and Clinical Research Network research delivery support. Further support for genome-wide and multiple ‘omics measurements in BiB is from the UK Medical Research Council (G0600705), National Institute of Health Research (NF-SI-0611-10196), US National Institute of Health (R01DK10324), and the European Research Council under the European Union’s Seventh Framework Programme (FP7/2007–2013) / ERC grant agreement no 669545.

UK Biobank

UK Biobank is funded primarily by the Wellcome Trust and the Medical Research Council (MRC). It is also funded by the Department of Health, British Heart Foundation, Cancer Research UK, Diabetes UK, National Institute for Health Research (NIHR), Scottish Government, Northwest Regional Development Agency, and Welsh Assembly Government.

For peer review only

For peer review only - http://bmjopen.bmj.com/site/about/guidelines.xhtml

References

1. Boyd A, Golding J, Macleod J, Lawlor DA, Fraser A, Henderson J, et al. Cohort profile: the 'children of the 90s'—the index offspring of the Avon Longitudinal Study of Parents and Children. Int J Epidemiol. 2013;42(1):111-27.
2. Fraser A, Macdonald-Wallis C, Tilling K, Boyd A, Golding J, Davey Smith G. Cohort profile: The Avon Longitudinal Study of Parents and Children: ALSPAC mothers cohort. Int J Epidemiol. 2013;42.
3. Wright J, on behalf of the Born in Bradford Scientific Collaborators G, Small N, Raynor P, Tuffnell D, Bhopal R, et al. Cohort Profile: The Born in Bradford multi-ethnic family cohort study. International Journal of Epidemiology. 2013;42(4):978-91.
4. Corfield EC, Frei O, Shadrin AA, Rahman Z, Lin A, Athanasiu L, et al. The Norwegian Mother, Father, and Child cohort study (MoBa) genotyping data resource: MoBaPsychGen pipeline v.1. bioRxiv. 2022:2022.06.23.496289.
5. Mitchell R, Hemani G, Dudding T, Corbin L, Harrison S, Paternoster L. UK Biobank Genetic Data: MRC-IEU Quality Control, version 2. 2019.
6. Bycroft C, Freeman C, Petkova D, Band G, Elliott LT, Sharp K, et al. The UK Biobank resource with deep phenotyping and genomic data. Nature. 2018;562(7726):203-9.
7. O'Connell J, Sharp K, Shrine N, Wain L, Hall I, Tobin M, et al. Haplotype estimation for biobank-scale data sets. Nat Genet. 2016;48(7):817.
8. Huang J, Howie B, McCarthy S, Memari Y, Walter K, Min JL, et al. Improved imputation of low-frequency and rare variants using the UK10K haplotype reference panel. Nature Communications. 2015;6:8111.
9. Howie B, Marchini J, Stephens M. Genotype Imputation with Thousands of Genomes. G3 Genes|Genomes|Genetics. 2011;1(6):457-70.
10. Manichaikul A, Mychaleckyj JC, Rich SS, Daly K, Sale M, Chen W-M. Robust relationship inference in genome-wide association studies. Bioinformatics. 2010;26(22):2867-73.
11. Kurki MI, Karjalainen J, Palta P, Sipilä TP, Kristiansson K, Donner KM, et al. FinnGen provides genetic insights from a well-phenotyped isolated population. Nature. 2023;613(7944):508-18.
12. FinnGen. Documentation of R12 release 2024 [Available from: https://finngen.gitbook.io/documentation/.
13. Purcell S, Chang C. PLINK. 2.00 alpha (build 22 Dec 2024) ed2024.
14. Chang CC, Chow CC, Tellier LC, Vattikuti S, Purcell SM, Lee JJ. Second-generation PLINK: rising to the challenge of larger and richer datasets. Gigascience. 2015;4(1):7.
15. Mbatchou J, Barnard L, Backman J, Marcketta A, Kosmicki JA, Ziyatdinov A, et al. Computationally efficient whole-genome regression for quantitative and binary traits. Nature Genetics. 2021;53(7):1097-103.
16. Abraham G, Qiu Y, Inouye M. FlashPCA2: principal component analysis of Biobank-scale genotype datasets. Bioinformatics. 2017;33(17):2776-8.
17. Abraham G. FlashPCA2 2016 [Available from: https://github.com/gabraham/flashpca.
18. Lawson D. R package pcapred 2020 [Available from: https://github.com/danjlawson/pcapred.
19. Ani A, van der Most PJ, Snieder H, Vaez A, Nolte IM. GWASinspector: comprehensive quality control of genome-wide association study results. Bioinformatics. 2021;37(1):129-30.
20. Willer CJ, Li Y, Abecasis GR. METAL: fast and efficient meta-analysis of genomewide association scans. Bioinformatics. 2010;26(17):2190-1.
21. Beaumont RN, Flatley C, Vaudel M, Wu X, Chen J, Moen G-H, et al. Genome-wide association study of placental weight in 179,025 children and parents reveals distinct and shared genetic influences between placental and fetal growth. medRxiv. 2022:2022.11.25.22282723.

For peer review only - http://bmjopen.bmj.com/site/about/guidelines.xhtml

22. Warrington NM, Beaumont RN, Horikoshi M, Day FR, Helgeland Ø, Laurin C, et al. Maternal and fetal genetic effects on birth weight and their relevance to cardio-metabolic risk factors. Nat Genet. 2019:1.
23. Warrington NM, Hwang L-D, Nivard MG, Evans DM. Estimating direct and indirect genetic effects on offspring phenotypes using genome-wide summary results data. Nature Communications. 2021;12(1):5420.
24. Wu Y, Zhong X, Lin Y, Zhao Z, Chen J, Zheng B, et al. Estimating genetic nurture with summary statistics of multigenerational genome-wide association studies. Proceedings of the National Academy of Sciences. 2021;118(25):e2023184118.
25. Bulik-Sullivan B, Finucane HK, Anttila V, Gusev A, Day FR, Loh P-R, et al. An atlas of genetic correlations across human diseases and traits. Nat Genet. 2015;47(11):1236-41.

For peer review only

For peer review only - http://bmjopen.bmj.com/site/about/guidelines.xhtml

The Mendelian Randomization in Pregnancy (MR-PREG) collaboration: Improving evidence on prevention, prediction and treatment of adverse pregnancy and perinatal outcomes

Supplementary figures

Avon Longitudinal Study of Parents and Children

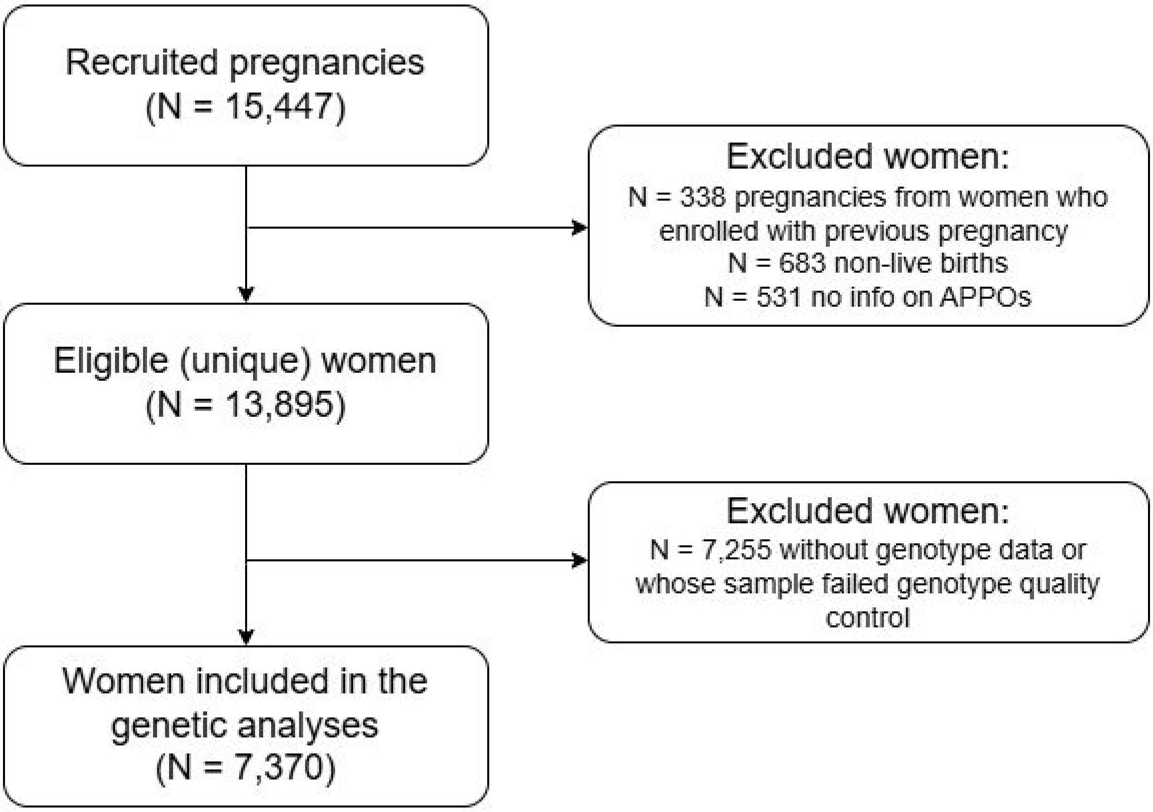

Genetic data is available for a subsample of mothers

Born in Bradford

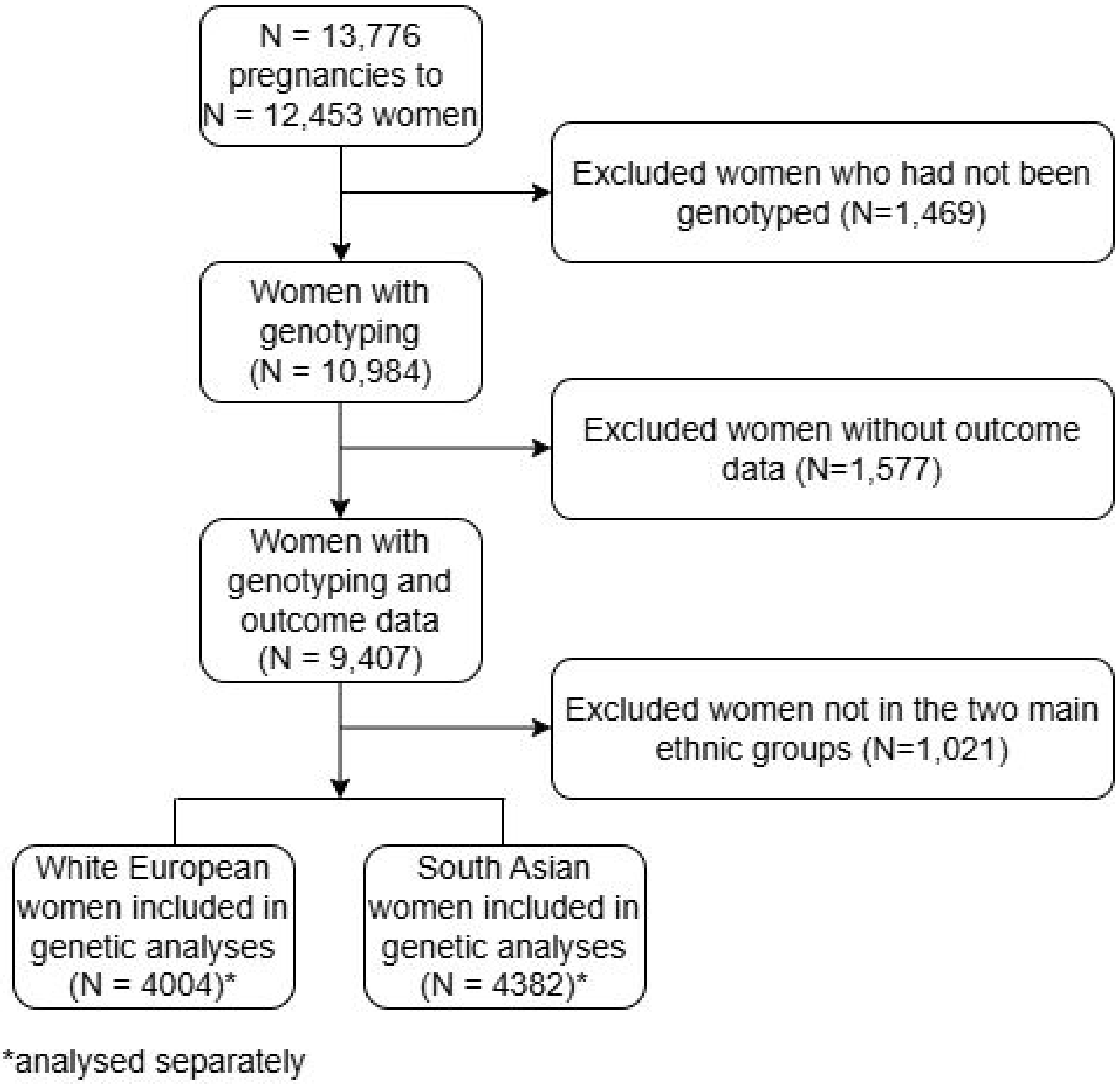

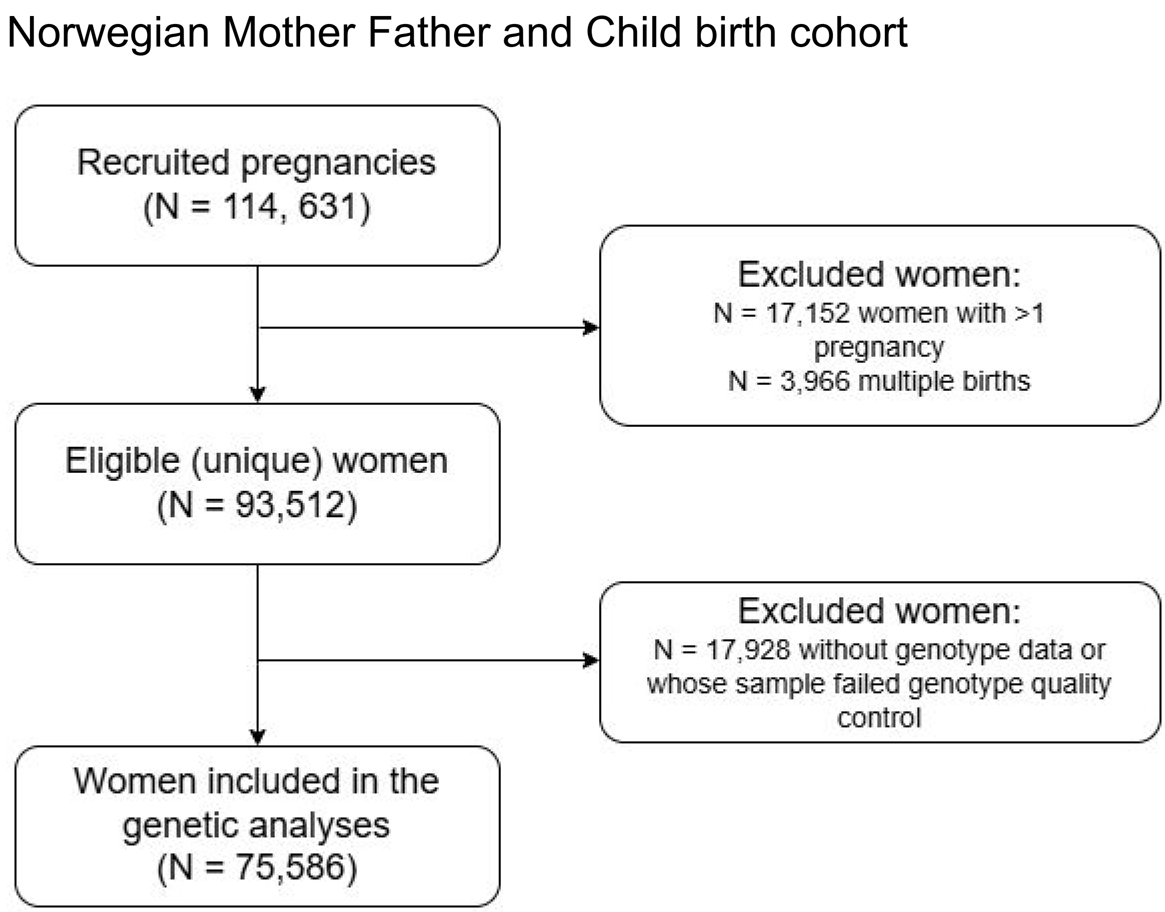
Norwegian Mother Father and Child birth cohort
Recruited pregnancies (N = 114, 631)
Excluded women:
N = 17,152 women with >1 pregnancy
N = 3,966 multiple births
Eligible (unique) women (N = 93,512)
Excluded women:
N = 17,928 without genotype data or whose sample failed genotype quality control
Women included in the genetic analyses (N = 75,586)

eview only

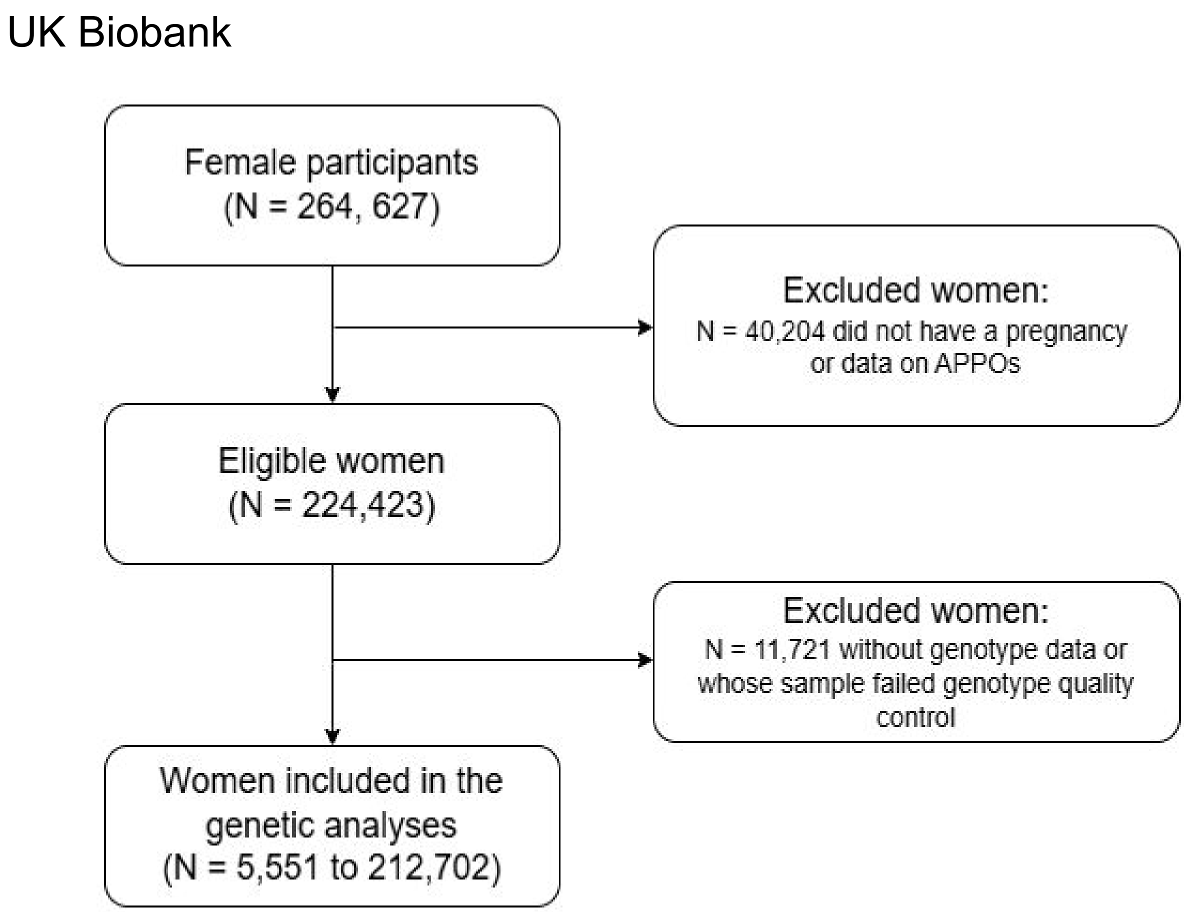

Note: some phenotypes have much smaller sample size because the source of information was maternity records or follow-up questionnaires, and these were not available for all women

Supplementary Tables

**ST1.**	Supplementary Table 1
**ST2A.**	Supplementary Table 2A
**ST2B.**	Supplementary Table 2B
**ST3A.**	Supplementary Table 3A
**ST3B.**	Supplementary Table 3B
**ST3C.**	Supplementary Table 3C
**ST3D.**	Supplementary Table 3D
**ST4A.**	Supplementary Table 4A
**ST4B.**	Supplementary Table 4B
**ST5.**	Supplementary Table 5

For peer review only

Summary of approaches that each have different assumptions that can help determine the causality of risk fac
Overview of definitions of (binary) APPOs and contributing studies
Overview of definitions of (continuous and categorical) APPOs and contributing studies
Number of cases and controls per APPO by each contributing study
Number of cases and controls - that contribute to maternal genetic analyses - per APPO by each contributing s
Number of cases and controls - that contribute to offspring and paternal genetic analyses - per APPO by each c
Overall N, mean and SD for continuous APPOs - split by overall N and N with maternal genotype by each contri
Summary of any deviations from the MR PREG (binary) APPO definition by each contributing study
Summary of any deviations from the MR PREG (continuous) APPO definition by each contributing study
Summary of the publicly available GWAS that were used for particular outcomes and the overlap with core MF

For peer review only

ctors (or mediation) on APPOs

study
contributing study
ributing study

R PREG studies

For peer review only

Type of study design/approach	Assumptions
**Conventional multivariable regression**	
To estimate the effect of a potential risk factor on adverse pregnancy outcomes (APPOs) by adjusting for observed confounders	All confounders are observed, correctly measured and modelled<br>No selection / measurement bias
**Negative control (exposure or outcome) study**	
To detect residual confounding or other biases by testing associations between the exposure and an outcome that should theoretically not be affected by the exposure, i.e., paternal factors which could not influence the maternal intrauterine environment	No plausible 'biological'/causal relationship between control exposure and outcome (or between exposure and control outcome)<br>Same confounders for negative control exposure/outcome as for main research question
**Mendelian randomisation (MR)**	
Uses genetic variants, which are randomly assigned at conception, that cannot be affected by the environment (i.e., confounding), to make causal statements	Strong relevant genetic instruments (when genetic variants explain the exposure variance)<br>No horizontal pleiotropy (when genetic variants affect the outcome through pathways other than the exposure)<br>No population stratification (i.e., no confounding by ancestry difference)<br>No selection bias or measurement error
**Cross-context comparisons**	
To assess causal effects by examining if relationships hold across different populations/time periods/contexts	Marked differences in confounders between contexts (populations), not just in their prevalence<br>No effect modification by context/population<br>Assumes unmeasured confounding which is similar in magnitude between contexts
**Within-family analyses**	
Comparing siblings with a differential exposure can control for genetic and family-level confounding (as siblings share many genetic and environmental factors)	Within sibling pairs, observed and unobserved confounders are matched<br>No or little individual confounding<br>Little individual measurement error<br>No crossover or selection bias
**Test-negative study design**	

For peer review only

To estimate the effect of an exposure (e.g., vaccination in pregnancy) on an outcome (e.g., APPO) by comparing test-positive (had vaccine) and test-negative (did not have vaccine) cases within a population	Assumes both groups (i.e., test negative and test positive) have undergone similar diagnostic procedures<br><br>Match test-positive and test-negative cases on key covariates<br>Assumes no misclassification biases (i.e., if cases are misclassified)<br><br>Assumes no selection bias (if individuals in both groups are systematically different)
**Natural experiment**	
To assess causal effects by using an external factor (e.g., policy change) as a quasi-random assignment of exposure	Assumes the external factor occurs randomly (i.e., the event or intervention (e.g., a new law or a natural disaster) should be unrelated to the participants' characteristics or outcomes before it occurred)<br><br>Assumes random assignment<br><br>Assumes no confounding from differences in populations exposed versus unexposed
**Target trial emulation**	
To mimic a randomized controlled trial (RCT) by using observational data, specifying eligibility criteria, treatment, exposure, and outcome measurement as if it were an RCT	By applying the correct study design to observational data (i.e., eligibility criteria) that we can emulate the causal question as in a real trial
**Drug target MR**	
Used to assess the causal effect of a drug target on disease outcomes by leveraging genetic variants as proxies, providing insights into the potential efficacy and safety of drug interventions	Assumes that genetic variants influencing the target gene or protein act as valid instruments, meaning they affect the disease outcome only through the drug target, not through other pathways

For peer review only

Rationale	Example
Quick and efficient insights through observational data	Effect of maternal body mass index (BMI) on APPOs
Reveals existence of potential unmeasured confounding<br>Can be extended to explore other types of bias (e.g., recall bias, selection bias)<br>Efficient insights through use of data already collected	Main hypothesis: Effect of maternal BMI on APPOs; Negative control exposure: Effect of paternal BMI on APPOs. If an association is found between paternal BMI and APPOs, this could indicate residual confounding in the main analysis
Less prone to reverse causation<br>Less prone to residual or unmeasured confounding	Effect of hyperglycaemia during pregnancy (instrumented by genetic variants) on APPOs
Can reveal context-specific confounding, selection bias or measurement error<br>Can examine the influence of high-level socioeconomic factors which are difficult to measure and fully account for	Breastfeeding and offspring health outcomes (e.g., blood pressure) across contexts (i.e., countries which have a very different socioeconomic pattern of breastfeeding)
Adjustment for a wide range of shared familial and parental characteristics<br>Control for genetic confounding (if identical twins)	The effect of assisted reproductive technologies (ART) vs natural conceptions on APPOs

For peer review only

Can help to control for unmeasured confounding: as both cases (those diagnosed with exposure) and controls (those not diagnosed) would have undergone similar diagnostic procedures	Effect of vaccination on APPOs - comparing test positive (had vaccine) and test negative (did not have vaccine)

When a natural event randomly affects some groups but not others (like a sudden policy change in one region but not another), it can help mimic randomisation, making comparisons between groups more reliable.<br>Can use existing real world data	Effect of air pollution on APPOs using implementation of low emission zone as natural experiment

Can answer key public health questions where a real trial might be too costly, too long or ethically impossible<br>Can avoid immortal time bias (i.e., when bias occurs due to including periods of time where people must survive to be included) by setting the causal question up correctly	The effect (efficacy and safety) of hypoglycaemics on APPOs

Infer causality<br>Can predict the potential impact of targeting specific proteins, aiding in drug development and prioritization	The effect of statins on pre-term birth

For peer review only

Type	Outcomes	Variables	Contributing studies[1] A	B	M	U	F	G*
Pregnancy	Hypertensive disorders of pregnancy	HDP	x	x	x			
						x	x	
	Gestational hypertension	GH	x	x	x			
						x	x	
	Pre-eclampsia	PE	x	x	x			x
						x	x	x
	Gestational diabetes	GDM			x			x
			x					x
						x	x	x
				x				x
	Perinatal depression	depr	x					x
				x				x
						x		x
					x			
	Anemia	anaemia_preg	x					
					x			
				x				
	Pregnancy loss	pregloss	x	x	x	x		
	Miscarriage	misc	x		x	x		
				x		x	x	
	Sporadic miscarriage	s_mis	x		x	x		
	Recurrent miscarriage	r_misc	x		x	x		
	Stillbirth	sb	x			x		
				x	x			
	Hyperemesis	hyp			x			
			x	x		x	x	
	Induction of labor	induction	x	x	x	x		
	Premature rupture of membranes	rup_memb	x		x		x	

review only

**Delivery**	Caesarian section	cs	x	x	x	x	x	
	Emergency C-section	em_cs	x	x	x	x		
	Elective C-section	el_cs	x	x	x	x		
	Very pre-term birth	vpretb	x	x	x	x		
	Pre-term birth	pretb	x	x	x	x	x	x
	Post-term birth	posttb	x	x	x	x	x	x
	Low birth weight	LBW	x	x	x	x		
	High birth weight	HBW	x	x	x	x		
	Small for gestational age	SGA	x	x	x	x		
	Large for gestational age	LGA	x	x	x	x		
	Low Apgar score at 1 minute	lowapgar1	x	x	x			
	Low Apgar score at 5 minutes	lowapgar5	x	x	x			
**Postnatal**	NICU admission	nicu	x		x			
	Congenital anomalies	conmal_any			x			
	Congenital heart disease	conmal_cvd			x			
	Breastfeeding initiation	bf_ini	x	x	x			
	Breastfeeding established	bf_est	x	x	x			
	Breastfeeding sustained	bf_sus	x	x	x			

[1] Contributing studies: ALSPAC (A), BiB (B), MoBa (M), UK Biobank (U), FinnGen (F), multiple pub

[2] Pavord S et al. UK guidelines on the management of iron deficiency in pregnancy. Br J Haemato

* Definition of cases and controls in the pubicly available GWAS might vary

**Case definition**	**Control definition**
Gestational hypertension or pre-eclampsia<br>ICD-10 codes: O13, O14	
SBP ≥ 140 mmHg or DBP ≥ 90 mmHg on at least 2 occasions after 20 gestational weeks, with no concurrent proteinuria<br>ICD-10 code: O13	No HDP
Gestational hypertension with concurrent proteinuria<br>ICD-10 code: O14	
Self-reported diabetes arising in pregnancy<br>Clinical diagnoses abstracted from medical records by research midwives<br>ICD-10 code: O24<br>Fasting glucose ≥ 6.1 mmol/L or 2 h post-load glucose ≥ 7.8 mmol/L in OGTT	No GDM
Mean EPDS total score across 12 and 32 gestational weeks and 8 weeks and 8 months postpartum > 12<br>Mean score of questions 22 to 28 in the GHQ administered at cohort registration during pregnancy > the 85th percentile<br>Self-reported antenatal depression<br>Mean of selective items from the (Hopkins) Symptoms Checklist-25 at 14 and	No depr
Maternal haemoglobin (Hb) < 100 g/L during pregnancy<br>Self reported anaemia/low haemoglobin at 15 weeks gestation<br>Clinical diagnosis abstracted from medical records	No anaemia_preg
Miscarriage or stillbirth in the index or previous pregnancies	No pregloss
Miscarriage in the index or previous pregnancies; miscarriage in the index pregnancy was defined as fetal loss before 20 weeks gestation; miscarriage history in previous pregnancies was self-reported in questionnaires<br>Miscarriage in the index or previous pregnancies; information on miscarriage was abstracted from medical records	
≤ 2 miscarriages in the index and previous pregnancies	
≥ 3 miscarriages in the index and previous pregnancies	
Stillbirth in the index or previous pregnancies; stillbirth in the index pregnancy was defined as fetal death before delivery at 20 gestational weeks or more; stillbirth history in previous pregnancies was self-reported in questionnaires<br>Stillbirth in the index or previous pregnancies; information on stillbirth was abstracted from medical records	
Self reported: excessive vomiting noted during pregnancy before onset of labour<br>Clinical diagnosis abstracted from medical records	No hyp
Medical induction needed during labour	No induction
Premature rupture of membranes at any gestational age	No rup_memb

For peer review only

Had a C-section	No cs
Had an emergency C-section	
Had an elective C-section	
Gestational age at birth < 34 weeks	Gestational age at birth ≥ 37 to < 42 weeks
Gestational age at birth < 37 weeks	
Gestational age at birth ≥ 42 weeks	
Birth weight < 2500 g	Birth weight ≥ 2500 g to ≤ 4500 g
Birth weight > 4500 g	
Birth weight-for-gestational age < 10th percentile	No SGA
Birth weight-for-gestational age > 90th percentile	No LGA
Apgar score at 1 minute < 7	No lowapgar1
Apgar score at 5 minutes < 7	No lowapgar5
Admission to Neonatal intensive care unit (NICU)	No NICU admission
Congenital malformations from the medical birth registry	No congenital anomalies
Congenital malformations (heart condition) from the medical birth registry	No congenital heart disease
Breastfeeding initiated	No bf_ini
Breastfeeding ≥ 2 months	No bf_est
Breastfeeding ≥ 6 months	No bf_sus

ɔlicly available GWAS (G)
ol. 2020;188(6):819-830.

For peer review only

Exclusions			
**Multiple births**	**Duplicated pregnancies**	**Non-live births**	**Other**
x	x	x	
x	x	x	Pre-existing hypertension
x	x	x	
x	x	x	Pre-existing diabetes
			Pre-existing depression
x	x	x	NA
x	x	x	NA
x	x		NA
x	x		NA
x	x		Age menarche < 9 or > 17 y and some
x	x		specific medical conditions
x	x		NA
x	x	x	NA
x	x	x	NA
x	x	x	NA

For peer review only

x	x	x	NA
x	x	x	NA
x	x	x	NA
x	x	x	NA
x	x	x	NA
x	x	x	NA
x	x	x	NA
x	x	x	NA
x	x	x	NA
x	x	x	NA
x	x	x	NA
x	x	x	NA
x	x	x	NA
x	x	x	NA
x	x	x	NA
x	x	x	NA
x	x	x	Never breastfed, preterm birth
x	x	x	

For peer review only

Type	Outcomes	Variables	Contributing studies[1]					
			A	B	M	U	F	G
**Pregnancy**	Severity of nausea and vomiting	nvp_sev	x		x			
**Delivery**	Birth weight	zBW	x	x	x			
						x		
	Gestational age at birth	GA	x	x	x	x		x
**Postnatal**	Breastfeeding duration	bf_dur_4c	x	x	x			

[1] Contributing studies: ALSPAC (A), BiB (B), MoBa (M), UK Biobank (U), FinnGen (F), multiple public

For peer review only

Source	Unit/definition
Self-reported in questionnaires	0 = None; 1 = Nausea only; 2 = Nausea and vomiting; 3 = On medication
Abstracted from medical records	SD units (Z-score transformed separately in males and females)
Self-reported in questionnaires	
Abstracted from medical records	Weeks
Self-reported in questionnaires	0 = never breasfed or < 1 month; 1 = 1-3 months; 2 = 4-6 months; 3 ≥ 6 months

:ly available GWAS (G)

For peer review only

Exclusions			
Multiple births	Duplicated pregnancies	Non-live births	Other
x	x	x	NA
x	x	x	Birth weight > 5 SD
x	x	x	NA
x	x	x	NA

For peer review only

Type	Outcomes	Acronym	Overall		ALS[illegible]
			Cases	Controls	Cases
Pregnancy	Hypertensive disorders of pregnancy	HDP	15,485	278,472	2,148
	Gestational hypertension	GH	11,134	278,971	1,865
	Pre-eclampsia	PE	4,428	278,969	283
	Gestational diabetes	GDM	2,772	333,614	55
	Depression	depr	12,669	122,588	772
	Anemia	anaemia_preg	4,354	102,045	1,001
	Pregnancy loss	pregloss	104,412	230,957	3,155
	Miscarriage	misc	73,462	230,957	3,045
	Sporadic miscarriage	s_mis	61,900	209,622	2,069
	Recurrent miscarriage	r_misc	6,007	209,732	153
	Stillbirth	sb	7,340	230,957	170
	Hyperemesis	hyp	1,298	274,857	81
	Severity of nausea and vomiting	nvp_sev		16,401	
	Nausea only		9,123		3,540
	Nausea and vomiting		34,776		5,946
	On medication		33,998		1,122
Delivery	Induction of labor	induction	18,098	100,985	1,502
	Premature rupture of membranes	rup_memb	15,011	85,761	2,143
	Caesarean section	cs	19,518	99,935	1,383
	Emergency C-section	em_cs	11,749	101,182	890
	Elective C-section	el_cs	7,497	101,226	493
	Very pre-term birth	vpretb	1,672	106,882	196
	Pre-term birth	pretb	6,227	106,814	695
	Post-term birth	posttb	8,893	106,774	1,032
	Low birth weight	LBW	4,357	115,392	605
	High birth weight	HBW	4,494	115,392	232
	Small for gestational age	SGA	9,729	112,726	1,058
	Large for gestational age	LGA	13,102	109,353	1,466
	Low Apgar score at 1 minute	lowapgar1	6,537	102,995	990
	Low Apgar score at 5 minutes	lowapgar5	1,290	108,248	125
Postntal	NICU admission	nicu	9,170	90,404	753
	Congenital anomalies	conmal_any	4,582	88,032	
	Congenital heart disease	conmal_cvd	797	91,817	
	Breastfeeding initiation	bf_ini	82,785	25,833	9,189
	Breastfeeding established	bf_est	71,564	31,296	5,897
	Breastfeeding sustained	bf_sus	54,481	26,208	3,490
	Breastfeeding duration	bf_dur_4c		29,423	
	1 to 3		8,417		2,077
	4 to 6		61,547		1,636
	>6		4,761		2,979

PAC	BiB		MoBa		UK Biobank	
Controls	Cases	Controls	Cases	Controls	Cases	Controls
10,692	764	6,831	11,669	80,774	904	180,175
10,865	563	6,831	8,129	80,774	577	180,501
10,692	201	6,831	3,540	80,774	404	180,672
11,835	765	8,233	803	91,356	1,149	222,190
9,923	972	6,033	4,649	80,190	6,276	26,442
7,013	1,850	6,169	1,503	88,863		
9,696	185	7,847	16,589	76,924	84,483	136,490
9,696	44	7,847	16,152	76,924	54,221	136,490
9,696			15,046	76,610	44,785	123,316
9,696			1,028	76,610	4,826	123,426
9,696	142	7,847	292	76,924	6,736	136,490
7,933	84	7,944	834	76,382	299	182,598
2,113			5,583	14,288		
			28,830			
			32,876			
6,359	1,961	7,384	12,370	80,224	2,265	7,018
5,630			12,868	80,131		
6,491	2,025	7,327	13,485	79,299	2,625	6,818
6,491	974	7,396	8,454	79,299	1,431	7,996
6,491	644	7,396	4,973	79,299	1,387	8,040
11,769	127	8,724	1,238	80,366	111	6,023
11,769	490	8,724	4,571	80,366	471	5,955
11,769	138	8,724	7,274	80,366	449	5,915
12,490	669	8,590	2,697	85,769	386	8,543
12,490	92	8,590	3,974	85,769	196	8,543
12,265	1,302	8,046	6,801	85,272	568	7,143
11,857	703	8,645	9,690	82,383	1,243	6,468
6,846	772	8,473	4,775	87,676		
7,708	115	9,127	1,050	91,413		
11,035			8,417	79,369		
			4,582	88,032		
			797	91,817		
2,707	2,255	955	71,341	22,171		
1,537	1,112	802	64,555	28,957		
3,944	673	1,241	50,318	21,023		
1,079	98	955	6,242	27,389		
	29		59,882			
	1,782					

For peer review only

Type	Outcomes	Acronym	Overall		ALSI
			Cases	Controls	Cases
Pregnancy	Hypertensive disorders of pregnancy	HDP	32,549	509,219	1,187
	Gestational hypertension	GH	20,777	507,155	1,029
	Pre-eclampsia^	PE	19,566	658,435	158
	Gestational diabetes^	GDM	26,346	908,220	
	Depression	depr	22,785	124,816	390
	Anemia	anaemia_preg	4,105	82,062	559
	Pregnancy loss	pregloss	94,152	204,716	1,524
	Miscarriage	misc	89,086	397,131	1,477
	Sporadic miscarriage	s_mis	55,888	185,271	1,156
	Recurrent miscarriage	r_misc	6,233	315,523	90
	Stillbirth	sb	6,331	201,339	70
	Hyperemesis	hyp	4,235	474,231	
	Severity of nausea and vomiting	nvp_sev		14,469	
	Nausea only		7,166		1,972
	Nausea and vomiting		30,133		3,312
	On medication		31,210		625
Delivery	Induction of labor	induction	14,495	80,744	849
	Premature rupture of membranes	rup_memb	21,839	288,782	1,212
	Caesarean section	cs	33,909	200,060	757
	Emergency C-section	em_cs	9,165	81,273	481
	Elective C-section	el_cs	6,004	81,279	276
	Very pre-term birth	vpretb	1,214	84,060	82
	Pre-term birth^	pretb	29,987	488,862	357
	Post-term birth^	posttb	27,841	397,832	573
	Low birth weight	LBW	18,617	256,597	293
	High birth weight	HBW	6,679	260,156	123
	Small for gestational age	SGA	6,882	82,151	
	Large for gestational age	LGA	10,468	85,837	811
	Low Apgar score at 1 minute	lowapgar1	4,950	81,718	544
	Low Apgar score at 5 minutes	lowapgar5	876	77,863	55
Postnatal	NICU admission	nicu	6,996	70,289	408
	Congenital anomalies	conmal_any	3,569	71,132	
	Congenital heart disease	conmal_cvd	612	74,089	
	Breastfeeding initiation	bf_ini	66,408	18,260	5,485
	Breastfeeding established	bf_est	58,035	23,223	3,698
	Breastfeeding sustained	bf_sus	44,401	20,788	2,254
	Breastfeeding duration	bf_dur_4c		23,515	
	1 to 3		6,372		1,270
	4 to 6		49,123		1,000
	>6		3,419		1,821

For the GWAS metanalyses, study-specifc results for a given APPO were excluded if the number of cases was lowe

For peer review only

PAC	BiB		MoBa		UK Biobank		FinnGen	
Controls	Cases	Controls	Cases	Controls	Cases	Controls	Cases	Controls
5,851	683	5,965	9,472	64,878	802	170,866	20,405	261,659
5,851	499	5,965	6,681	64,877	518	171,149	12,050	259,313
5,851	184	5,965	2,628	60,335	354	171,311	9,023	259,313
	630	7,088	602	73,507	964	210,985	18,581	263,483
5,790			2,889	52,282	6,158	25,617		
3,901	1,597	5,421	1,949	72,740				
5,470	167	6,865	13,451	61,563	79,010	130,818		
5,470			13,116	61,564	51,326	130,818	23,167	199,279
5,470			12,227	61,336	42,505	118,465		
5,470			832	61,336	4,500	118,557	811	130,160
5,470	87	3,488	223	61,563	5,951	130,818		
			656	63,115	250	173,210	3,329	237,906
1,177				13,292				
			5,194					
			26,821					
			30,585					
3,538	1,686	6,333	9,915	64,563	2,045	6,310		
3,127			10,219	64,469			10,408	221,186
3,636	1,700	6,324	10,582	64,019	2,303	6,191	18,567	119,890
3,636	836	6,382	6,605	64,019	1,243	7,236		
3,636	561	6,382	3,929	64,020	1,238	7,241		
6,440	107	7,484	940	64,670	85	5,466		
6,440	414	7,484	2,892	48,159	406	5,403	11,405	214,925
6,440	126	7,484	4,543	48,160	424	5,360	7,049	221,186
6,858			2,038	69,079	16,286	180,660		
6,858	64	3,560	3,244	69,078	3,248	180,660		
	1,114	6,907	5,303	68,765	465	6,479		
6,461	626	7,395	7,865	66,203	1,166	5,778		
3,831	628	7,302	3,778	70,585				
4,316			821	73,547				
6,327			6,588	63,962				
			3,569	71,132				
			612	74,089				
1,307	1,977	885	58,946	16,068				
859	962	725	53,375	21,639				
2,303	556	1,131	41,591	17,354				
660		856		21,999				
	88		5,014					
	26		48,097					
	1,598							

er than 50 or if the study had already been included by publicly available GWAS.

For peer review only

GWAS	
**Cases**	**Controls**
7,219	155,660
5,569	353,157
13,348	41,127
14,513	206,451
15,126	109,202

For peer review only

Type	Outcomes	Acronym	Overall Offspring Cases	Overall Offspring Controls	ALS Offsp Cases
Pregnancy	Hypertensive disorders of pregnancy	HDP	9,653	64,516	1,215
	Gestational hypertension	GH	7,227	64,516	1,060
	Pre-eclampsia	PE	2,263	59,673	155
	Gestational diabetes	GDM	990	66,772	
	Depression^	depr	3,795	65,973	399
	Anemia	anaemia_preg	3,439	67,875	533
	Pregnancy loss	pregloss	12,988	58,567	1,566
	Miscarriage	misc	12,698	55,988	1,531
	Sporadic miscarriage	s_mis	11,871	55,989	1,432
	Recurrent miscarriage	r_misc	822	55,989	95
	Stillbirth	sb	389	71,167	61
	Hyperemesis	hyp	484	42,872	
	Severity of nausea and vomiting	nvp_sev		11,505	
	Nausea only		6,019		1,987
	Nausea and vomiting		24,158		3,337
	On medication		24,373		630
Delivery	Induction of labor	induction	10,094	62,012	840
	Premature rupture of membranes	rup_memb	9,724	56,207	1,218
	Caesarean section	cs	9,592	62,574	740
	Emergency C-section	em_cs	5,594	62,590	460
	Elective C-section	el_cs	3,647	62,590	280
	Very pre-term birth	vpretb	492	60,574	80
	Pre-term birth	pretb	2,888	66,419	337
	Post-term birth	posttb	5,556	63,119	561
	Low birth weight	LBW	1,799	70,252	260
	High birth weight	HBW	2,919	67,058	130
	Small for gestational age	SGA	5,391	69,381	512
	Large for gestational age	LGA	7,988	66,784	852
	Low Apgar score at 1 minute	lowapgar1	3,647	68,352	508
	Low Apgar score at 5 minutes	lowapgar5	492	61,000	
Postnatal	NICU admission	nicu	4,993	60,080	382
	Congenital anomalies	conmal_any	2,665	58,824	
	Congenital heart disease	conmal_cvd	439	61,050	
	Breastfeeding initiation	bf_ini	58,700	12,017	5,611
	Breastfeeding established	bf_est	51,357	16,290	3,849
	Breastfeeding sustained	bf_sus	39,199	18,412	2,359
	Breastfeeding duration	bf_dur_4c		15,908	
	1 to 3		5,508		1,323
	4 to 6		40,573		1,042
	>6		3,096		1,898

For the GWAS metanalyses, study-specifc results for a given APPO were excluded if the number of cases was l

PAC	BiB		MoBa			
pring	Offspring		Offspring		Fathers	
Controls	Cases	Controls	Cases	Controls	Cases	Controls
6,116	517	4,766	7,921	53,634	6,476	42,973
6,116	382	4,766	5,785	53,634	4,546	42,972
5,852	127	4,529	1,981	49,292	1,930	42,876
	510	5,655	480	61,117	391	49,127
5,901	586	3,918	2,810	56,154	2,075	44,092
3,910	1,241	4,033	1,665	59,932	1,198	48,370
5,486	68	2,812	11,354	50,269	8,852	40,926
5,532			11,167	50,456	8,719	41,059
5,532			10,439	50,457	8,167	41,059
5,532			727	50,457	552	41,060
6,992	53	2,827	275	61,348	193	49,585
					484	42,872
1,186				10,319		8,106
			4,032		3,167	
			20,821		16,355	
			23,743		18,651	
3,535	1,254	4,932	8,000	53,545	6,784	42,649
3,116			8,506	53,091	7,190	42,378
3,640	1,447	4,742	7,405	54,192	7,174	42,344
3,640	637	4,758	4,497	54,192	4,569	42,343
3,640	490	4,758	2,877	54,192	2,579	42,344
6,484			412	54,090	691	42,819
6,484	259	5,845	2,292	54,090	2,424	42,819
6,484	50	2,546	4,945	54,089	3,991	42,818
6,900	357	5,758	1,182	57,594	1,410	45,904
6,900	53	2,564	2,736	57,594	2,046	45,904
6,776	823	5,365	4,056	57,240	3,545	45,619
6,436	495	5,693	6,641	54,655	5,071	44,093
3,854	438	5,716	2,701	58,782	2,564	46,795
			492	61,000	564	48,803
6,502			4,611	53,578	4,606	42,835
			2,665	58,824	2,448	47,140
			439	61,050	430	49,158
1,324	1,502	657	51,587	10,036	39,683	10,095
904	732	539	46,776	14,847	35,918	13,861
2,394	433	838	36,407	15,180	30,075	9,607
687		642		18,072		14,579
	66		4,119		3,323	
	19		39,512		31,876	
	1,198					

ower than 50 or if the study had already been included by publicly available GWAS.

For peer review only

Type	Outcomes	Acronym	Overall			
			N phenotype	N genotype	Mean	SD
Delivery	Birth weight (grams)	BW	328,256	289,846	3376.7	601.9
	Gestational age (weeks)	GA	126,151	226,810	39.5	1.9

For the GWAS metanalyses, study-specifc results for a given outcome were excluded if the study had already [illegible]

For peer review only

ALSPAC				BiB				
N phenotype	N genotype	Mean	SD	N phenotype	N genotype	Mean	SD	N phenotype
13,354	7,273	3408.9	555	12,346	8,023	3225.9	557	92,440
13,496	7,370	39.4	1.9	12,346	8,024	39.5	1.8	92,211

been included by publicly available GWAS.

For peer review only

MoBa			UK Biobank				GWAS	
N genotype	Mean	SD	N phenotype	N genotype	Mean	SD	N	Mean
74,361	3596	558	210,116	200,189	3239.4	640	NA	NA
55,595	39.5	1.9	8,098	7,303	39.5	1.7	148,518	NA

For peer review only

**SD**
NA
NA

For peer review only

Type	Outcomes	Variables	ALSPAC
Pregnancy	Gestational hypertension	GH	-
	Pre-eclampsia	PE	-
	Hypertensive disorders of pregnancy	HDP	-
	Gestational diabetes	GDM	-
	Hyperemesis	hyp	-
	Severity of nausea and vomiting	nvp_sev	-
	Depression	depr	-
	Anemia	anaemia_preg	Cases defined as maternal haemoglobin (Hb) < 100 g/L during pregnancy
	Pregnancy loss	pregloss	-
	Miscarriage	misc	Cases defined as pregnancy loss before 20 weeks gestation
	Sporadic miscarriage	s_mis	-
	Recurrent miscarriage	r_misc	-
	Stillbirth	sb	Cases defined as pregnancy loss at ≥20 weeks gestation
	Induction of labor	induction	-

For peer review only

Delivery	Premature rupture of membranes	rup_memb	-
	Caesarean section	cs	-
	Emergency C-section	em_cs	-
	Elective C-section	el_cs	-
	Very pre-term birth	vpretb	-
	Pre-term birth	pretb	-
	Post-term birth	posttb	-
	Low birth weight	LBW	NA
	High birth weight	HBW	-
	Small for gestational age	SGA	-
	Large for gestational age	LGA	-
	Low Apgar score at 1 minute	lowapgar1	-
	Low Apgar score at 5 minutes	lowapgar5	-
	NICU admission	nicu	-

For peer review only

**Postnatal**	Congenital anomalies	CA	NA
	Congenital heart disease	CHD	NA
	Breastfeeding initiation	bf_ini	-
	Breastfeeding established	bf_est	-
	Breastfeeding sustained	bf_sus	-
	Breastfeeding duration	bf_dur_4c	-

_: no deviations from MR-PREG definition; NA: not available

For peer review only

Deviations from the collaboration definition		
BiB	MoBa	UK Biobank
-	-	-
-	-	-
-	-	-
-	-	-
-	-	-
NA	-	NA
Only antenatal depression available, derived from GHQ; pre-existing depression not excluded	-	Depression possibly related to childbirth, based on self-report
-	Self reported ~ 15 weeks gestation	NA
-	-	-
-	Self report cases defined as pregnancy loss before 23 weeks gestation (previous not index)	Self-reported cases might not be pregnancy loss at < 22 gestational weeks; no index pregnancy
-	-	Self-reported cases might not be pregnancy loss at < 22 gestational weeks; no index pregnancy
-	-	Self-reported cases might not be pregnancy loss at < 22 gestational weeks; no index pregnancy
-	Self report cases defined as pregnancy loss at ≥21 weeks gestation (previous not index)	Self-reported cases might not be pregnancy loss at ≥ 20 gestational weeks; no index pregnancy
-	-	No index pregnancy; random birth selected from available data

For peer review only

-	-	NA
-	-	No index pregnancy; random birth selected from available data
-	-	No index pregnancy; random birth selected from available data
-	-	No index pregnancy; random birth selected from available data
-	-	No index pregnancy; random birth selected from available data
-	-	No index pregnancy; random birth selected from available data
-	-	No index pregnancy; random birth selected from available data
-	-	No index pregnancy; random birth selected from available data
-	-	No index pregnancy; random birth selected from available data
-	-	No index pregnancy; random birth selected from available data
-	-	No index pregnancy; random birth selected from available data
-	-	NA
-	-	NA
NA	-	NA

For peer review only

NA	-	NA
NA	-	NA
BF questionnaire contained different questions than the other cohorts*	-	NA
BF questionnaire contained different questions than the other cohorts*	-	NA
BF questionnaire contained different questions than the other cohorts*	-	NA
BF questionnaire contained different questions than the other cohorts*	No last category of 6+ months	NA

For peer review only

FinnGen	GWAS
-	NA
-	ICD codes or ISSHP criteria
-	NA
ICD-9 code also used, pre-existing diabetes included	NA
ICD-10 O21, ICD-9 643, ICD-8 638	NA
NA	NA
NA	NA
NA	NA
NA	NA
ICD-10 O03, ICD-9 634, ICD-8 643	NA
NA	NA
ICD-10 N96	NA
NA	NA
NA	NA

For peer review only

ICD-10 O42, ICD-9 6581A, ICD-8 63495	NA
ICD-10 O82	NA
NA	NA
NA	NA
NA	NA
ICD-10 O60, ICD-9 644, ICD-8 63497, excluded ICD-9 6440A	cases: <37 gestational weeks, controls: 39-42 gestational weeks, singleton live birth & a spontaneous onset of delivery without congenital malformations, healthy mothers
ICD-10 O48, ICD-9 645	cases: >42 & <=44 gestational weeks, controls: 39-42 gestational weeks, singleton live birth & a spontaneous onset of delivery without congenital malformations, healthy mothers
NA	NA
NA	NA
NA	NA
NA	NA
NA	NA
NA	NA
NA	NA

For peer review only

NA	NA
NA	NA
NA	NA
NA	NA
NA	NA
NA	NA

For peer review only

Type	Outcomes	Variables	ALSPAC
Delivery	Birth weight	zBW	-
	Gestational age	GA	-

_: no deviations from MR-PREG definition; NA: not available

For peer review only

Deviations from the collaboration definition		
BiB	MoBa	UK Biobank
-	-	Based on self-reported birth weight of first child; not possible to standardise by sex
-	-	No index pregnancy; random birth selected from available data

For peer review only

FinnGen	GWAS
NA	NA
NA	NA

For peer review only

Study name	Trait name
**GenDiP**	Gestational diabetes
**EGG**	Gestational age
**EGG**	Pre-term birth
**EGG**	Post-term birth
**InterPregGen**	Pre-eclampsia
**PGC**	Post-natal depression

For peer review only

**Definition**
Cohort-specific definition, including data from health records, oral glucose tolerance tests and self-report
Days, estimated using either the last menstrual period date or ultrasound
<259 days<br>ICD-10 O60
>294 days<br>ICD-10 O48
New-onset SBP ≥ 140 mmHg or DBP ≥ 90 mmHg on at least two occasion after 20th week gestation or ICD-10 [illegible]
Cohort-specific definition of postpartum depression

For peer review only

Number of participants (N [illegible]	Maternal/fetal genotype[illegible]
N= 353,341 (5,485 cases)	Maternal
N= 195,555	Maternal/fetal
N= 279,043 (18,797 cases)	Maternal/fetal
N= 131,279 (15,972 cases)	Maternal/fetal
N= 377,975 (4,630 cases)	Maternal in all, maternal
N= 70,765 (17,339 cases)	Maternal

For peer review only

Phenotype transformations
Phenotype definition/covariates were not harmonized between GWAS due to differ
None specified
None specified
None specified
None specified
None specified

For peer review only

Covariables
PC adjustment in most studies, only additional covariates in 3 studies (age, age + BMI and age + genotyping ar
PCs or relationship matrix on autosomal chromosomes/chromosome X
PCs or relationship matrix on autosomal chromosomes/chromosome X
PCs or relationship matrix on autosomal chromosomes/chromosome X
Genotype counts from imputation, deCODE = county of origin, other cohorts = top five PCs as covariates, FINR
PCs or relationship matrix on autosomal chromosomes/chromosome X

For peer review only

**Cohorts needed to be excluded**
ALSPAC, BIB, UK Biobank
ALSPAC, MOBA
ALSPAC, MOBA
ALSPAC, MOBA
ALSPAC
ALSPAC, MOBA, UKB

For peer review only

BMJ Open

Cohort Profile: The Mendelian Randomization in Pregnancy (MR-PREG) collaboration - Improving evidence for prevention and treatment of adverse pregnancy and perinatal outcomes

Journal:	*BMJ Open*
Manuscript ID	bmjopen-2025-103753.R1
Article Type:	Cohort profile
Date Submitted by the Author:	04-Feb-2026

Complete List of Authors:	McBride, Nancy; MRC Integrative Epidemiology Unit at the University of Bristol<br>Clayton, Gemma; MRC Integrative Epidemiology Unit<br>Soares, Ana Luiza; University of Bristol, Population Health Sciences<br>Yang, Qian ; MRC Integrative Epidemiology Unit at the University of Bristol; Shanghai Jiao Tong University School of Medicine, Department of Endocrine and Metabolic Diseases; Shanghai Jiao Tong University School of Medicine<br>Bond, Tom A; MRC Integrative Epidemiology Unit at the University of Bristol; University of Queensland<br>Taylor, Amy; MRC Integrative Epidemiology Unit at the University of Bristol; University College London<br>Chatzigeorgiou, Charikleia; MRC Integrative Epidemiology Unit at the University of Bristol<br>Aiton, Elisabeth; MRC Integrative Epidemiology Unit at the University of Bristol<br>West, Jane; University of Sheffield, Population Health; Bradford Teaching Hospitals<br>Magnus, Maria; Norwegian Institute of Public Health<br>Lawlor, Deborah; MRC Integrative Epidemiology Unit at the University of Bristol<br>Borges, Maria Carolina ; MRC Integrative Epidemiology Unit at the University of Bristol

**Primary Subject Heading**:	Epidemiology
Secondary Subject Heading:	Genetics and genomics, Public health, Obstetrics and gynaecology
Keywords:	Pregnancy, EPIDEMIOLOGY, Mendelian Randomization Analysis

SCHOLARONE™
Manuscripts

I, the Submitting Author has the right to grant and does grant on behalf of all authors of the Work (as defined in the below author licence), an exclusive licence and/or a non-exclusive licence for contributions from authors who are: i) UK Crown employees; ii) where BMJ has agreed a CC-BY licence shall apply, and/or iii) in accordance with the terms applicable for US Federal Government officers or employees acting as part of their official duties; on a worldwide, perpetual, irrevocable, royalty-free basis to BMJ Publishing Group Ltd ("BMJ") its licensees and where the relevant Journal is co-owned by BMJ to the co-owners of the Journal, to publish the Work in this journal and any other BMJ products and to exploit all rights, as set out in our licence.

The Submitting Author accepts and understands that any supply made under these terms is made by BMJ to the Submitting Author unless you are acting as an employee on behalf of your employer or a postgraduate student of an affiliated institution which is paying any applicable article publishing charge ("APC") for Open Access articles. Where the Submitting Author wishes to make the Work available on an Open Access basis (and intends to pay the relevant APC), the terms of reuse of such Open Access shall be governed by a Creative Commons licence – details of these licences and which Creative Commons licence will apply to this Work are set out in our licence referred to above.

Other than as permitted in any relevant BMJ Author's Self Archiving Policies, I confirm this Work has not been accepted for publication elsewhere, is not being considered for publication elsewhere and does not duplicate material already published. I confirm all authors consent to publication of this Work and authorise the granting of this licence.

For peer review only

Cohort Profile: The Mendelian Randomization in Pregnancy (MR-PREG) collaboration - Improving evidence for prevention and treatment of adverse pregnancy and perinatal outcomes

McBride, Nancy [1,2]* [nancy.mcbride@bristol.ac.uk]

Clayton, Gemma L[1,2]* [gemma.clayton@bristol.ac.uk, corresponding author]

Goncalves Soares, Ana[1,2]* [analuiza.soares@bristol.ac.uk]

Yang, Qian[1,2,3,4] [qian.yang@bristol.ac.uk]

Bond, Tom A[1,2,5] [tom.bond@bristol.ac.uk]

Taylor, Amy[1,2,6] [amy.taylor@bristol.ac.uk]

Chatzigeorgiou, Charikleia[1,2] [fb23004@bristol.ac.uk]

Aiton, Elisabeth[1,2] [e.aiton@bristol.ac.uk]

West, Jane[7,8] [jane.west@bthft.nhs.uk]

Magnus, Maria C[9] [mariachristine.magnus@fhi.no]

Lawlor, Deborah A[1,2]** [d.a.lawlor@bristol.ac.uk]

Borges, Maria Carolina[1,2]** [m.c.borges@bristol.ac.uk]

*Joint first author

**Joint last author

Corresponding author: Gemma L Clayton, gemma.clayton@bristol.ac.uk, MRC Integrative Epidemiology Unit, University of Bristol, Oakfield House, Oakfield Grove, Bristol, BS8 2BN, United Kingdom, Tel: +44 (0)117 331 0098

Keywords: Pregnancy, epidemiology, Mendelian randomization, triangulation, causal inference

Word count: 4389

For peer review only - http://bmjopen.bmj.com/site/about/guidelines.xhtml

For peer review only

Affiliations

[1]MRC Integrative Epidemiology Unit at the University of Bristol

[2]Population Health Science, Bristol Medical School, University of Bristol

[3]Department of Endocrine and Metabolic Diseases, Shanghai Institute of Endocrine and Metabolic Diseases, Ruijin Hospital, Shanghai Jiao Tong University School of Medicine, Shanghai, China

[4]Shanghai National Clinical Research Center for Metabolic Diseases, Key Laboratory for Endocrine and Metabolic Diseases of the National Health Commission of the PR China, Shanghai Key Laboratory for Endocrine Tumor, Lifecycle Health Management Center, Ruijin Hospital, Shanghai Jiao Tong University School of Medicine, Shanghai, China

[5]Frazer Institute, University of Queensland, Woolloongabba, Australia

[6]Division of Surgery and Interventional Science, University College London, London

[7]Population Health, School of Medicine and Population Health, University of Sheffield

[8]Bradford Institute for Health Research, Bradford Teaching Hospitals, Bradford, UK; Department of Health Sciences, University of York, York, UK

[9]Centre for Fertility and Health, Norwegian Institute of Public Health, Oslo, Norway.

For peer review only

Abstract

Purpose

Adverse pregnancy and perinatal outcomes (APPOs), including pre-term birth, pre-eclampsia, and gestational diabetes, can result in maternal and neonatal morbidity and mortality, parental anxiety, and increased health care costs. A better understanding of the causes of APPOs is essential to inform lifestyle and pharmaceutical interventions for their prevention and management. Given the difficulty of undertaking randomised controlled trials in pregnant women, triangulating evidence from across methods with different sources of bias may improve causal inference for APPOs. The purpose of the Mendelian Randomization in Pregnancy (MR-PREG) collaboration is to support such triangulation using genetic (e.g., Mendelian randomization [MR]) and non-genetic (e.g., partner negative controls) approaches to investigate the causal effects of maternal exposures on a comprehensive set of APPOs.

Participants

The MR-PREG collaboration includes individual participant data from three birth cohorts (two from the UK and one from Norway) and UK Biobank, as well as summary data from FinnGen and publicly available genome-wide association studies (GWAS). Data have been harmonised across studies and currently include information on up to 35 APPOs in up to 714,899 women.

Findings to date

The main aims of MR-PREG are to strengthen the evidence base for 1) prevention, by advancing understanding of maternal lifestyle factors on APPOs, 2) the role of pre-conceptional health, by improving understanding of the effect of maternal pre-existing conditions on APPOs, and 3) treatments, by evaluating the efficacy and safety of existing medications used for pre-existing conditions, and by identifying and testing novel or repurposed therapies for APPOs. To date, our published work has mainly addressed aims 1 and 3. Examples include triangulation of evidence from MR, conventional multivariable regression and paternal negative control, showing that higher maternal body mass index increases the risk of multiple APPOs, as well as the identification of maternal circulating metabolites and proteins that may influence birthweight.

Future Plans

For peer review only - http://bmjopen.bmj.com/site/about/guidelines.xhtml

Future priorities include increasing diversity within the MR-PREG collaboration by expanding representation of participants from non-European ancestries. We are also integrating molecular data, including circulating protein levels and placental transcriptomics, to better characterise the molecular mechanisms underlying APPOs. Additionally, we are using whole-exome and whole-genome sequencing to identify novel causal genes and to inform the prioritisation of candidate therapeutic targets for APPOs.

Strengths and Limitations

- We have curated data on 35 APPOs and harmonized data across multiple studies to support large-scale investigations of causes of APPOs.
- The breadth and nature of the data support triangulation of evidence from a range of genetic and non-genetic methods, each with distinct sources of bias, to identify causes of APPOs.
- Over the coming year, we will further enhance the data to enable identification of molecular mechanisms underlying APPOs.
- MR-PREG has limited power to detect causal effects for rarer APPOs, such as congenital anomalies and low Apgar scores at 5 minutes, particularly when using genetic methods. Future work will incorporate larger samples and rare genetic variant data.
- Participants are predominantly of European ancestry; future efforts will focus on increasing ancestral diversity within the data.

For peer review only

For peer review only - http://bmjopen.bmj.com/site/about/guidelines.xhtml

Why was the collaboration established?

A substantial proportion of pregnant women experience adverse pregnancy and perinatal outcomes (APPOs), including miscarriage, stillbirth, gestational diabetes mellitus (GDM), gestational hypertension (GH), pre-eclampsia (PE), pre-term birth (PTB), or having a baby who is small (SGA) or large for gestational age (LGA). Globally, one in six recognised pregnancies results in miscarriage [1], one in six women develop GDM [2], one in ten women experience hypertension during pregnancy [3], and one in ten babies is born preterm [4]. Some APPOs are major causes of severe morbidity and mortality for mothers and their babies [5]. For illustration, hypertensive disorders of pregnancy (HDP), including GH and PE, account for approximately 14% of all maternal deaths worldwide [6]. In addition, APPOs are associated with long-term adverse physical and mental health outcomes, as well as substantial costs to families, healthcare systems and society [7 8]. In the UK, the short-term costs of miscarriage alone are estimated to be around £471 million per year, reflecting costs to health services, families and loss of productivity [1].

The marked variation in the prevalence of several APPOs across regions and over time, although influenced by different screening and diagnostic practices, indicates that many APPOs are at least partly preventable [2]. A better understanding of the underlying causes of APPOs is therefore crucial for guiding effective interventions to prevent them. Such knowledge would also enable antenatal care services to provide women and couples with accurate advice on the most plausible risk factors for reducing APPOs, potentially reducing the often-conflicting advice given to women about exposures that could impact their own and their babies' health during pregnancy. Furthermore, APPOs are often related, meaning that preventing one may help reduce the burden of others. For example, both GDM and HDP can influence fetal growth, leading to the delivery of LGA and SGA babies, respectively [9 10]. Preventing GDM and HDP is, therefore, a way of avoiding fetal over/under growth and the related complications.

In addition to lifestyle and health-related risk factors, there is also an urgent need to better understand the effects of pharmaceutical treatments during pregnancy. This includes therapies for the prevention and management of APPOs, as well as for the treatment of pre-existing conditions that are increasingly common among women of reproductive age, such as autoimmune, mental health, thyroid, reproductive, and cardiometabolic disorders [11-14]. An

For peer review only - http://bmjopen.bmj.com/site/about/guidelines.xhtml

increasing proportion of women start pregnancy on medication, and the use of medication among pregnant women has also been rising over the past few decades [15]. Despite this, pregnant women are rarely included in pre-licensure randomized controlled trials (RCTs) largely due to concerns about potential teratogenic effects and uncertainty regarding drug dosing in the context of pregnancy-related physiological changes [16 17]. As a result, evidence on the benefits and risks of drugs for mother and baby is poor, investment in new drug development for APPOs is scarce, and very few medications are explicitly licensed for use during pregnancy. This situation exposes mothers and babies to unknown risks of drugs, compels pregnant women and their doctors to undertreat medical conditions, and forces them to make difficult decisions about whether to prescribe or continue medications and at what dose. It also means that APPOs, such as GDM and GH, are managed less well than equivalent conditions outside of pregnancy (i.e., type 2 diabetes and hypertension) [16 17].

RCTs are the gold-standard method for testing the impact of lifestyle and pharmaceutical interventions to prevent or treat APPOs. For several years, national bodies have highlighted the need for clinical trials of medications in pregnant women, largely to no avail [16 18]. Reflecting this challenge, the UK 2021 Report of the Commission on Human Medicines Expert Working Group on Optimising Data On Medicines Used During Pregnancy recommended improved use, linkage, and access to routinely collected healthcare data to support high-quality observational research and pharmacovigilance on the safety of medicines used during pregnancy and breastfeeding [18].

In the absence of well-powered, well-conducted RCTs, we need to make the best use of observational data to improve the current evidence base on causes of APPOs and medication efficacy and safety during pregnancy. More than 20 years ago, the use of genetic variants to infer causal effects of exposures − a method known as Mendelian randomization (MR) − was first proposed [19]. MR leverages the random allocation of genetic variants at conception to investigate the effects of modifiable risk factors on health outcomes. This method mitigates confounding by socioeconomic, behavioural, or health-related factors that frequently affect traditional observational study analyses (**Figure 1**) [19-21]. Since then, its application has expanded significantly, including for assessing the effects of a few maternal risk factors on a small range of APPOs (e.g. [22 23 24]).

For peer review only - http://bmjopen.bmj.com/site/about/guidelines.xhtml

For peer review only

Building on this framework, MR has also been extended to evaluate the effects of medications through genetic proxies for drug targets (also referred to as drug target MR). The validity of drug target MR is supported by proof-of-concept studies comparing MR findings to RCT results for established medications, such as antihypertensives and statins [25]. Drug targets supported by genetic evidence have higher success rates and genetic evidence (including from MR) is increasingly used to prioritise (or de-prioritise) new drug targets to be tested in RCTs [26-28].

As with all methods, MR is limited by violation of its assumptions. Triangulation of evidence acknowledges and exploits the fact that all methods have sources of bias [29]. It involves integrating multiple lines of evidence using one or more different approaches (e.g., different analytical methods, different data sources, or different study designs) with distinct and unrelated key sources of bias. If results are consistent, this increases the credibility of that being the correct causal effect, as it would be unlikely for different biases to produce similar results. Where there is disagreement across the different methods, prior specification of key sources of bias, the direction of these biases, and statistical efficiency for each method can help inform whether the inconsistency is explained by the different biases or power for each approach. This process can then inform the choice of additional sensitivity analyses or complementary approaches needed to strengthen causal inference.

The Mendelian Randomization in Pregnancy (MR-PREG) collaboration was established to improve causal understanding of the effects of maternal lifestyle and health factors on APPOs, as well as efficacy and safety of medication use in pregnancy. In this paper, we outline the aims of the MR-PREG collaboration, describe the characteristics of the contributing studies, detail our approaches to data generation and causal analysis, and summarise findings to date while highlighting ongoing and future work.

Aims of the MR-PREG collaboration

Aim 1: Using triangulation of evidence to improve knowledge on the impact of maternal lifestyle factors on APPOs

The MR-PREG collaboration uses triangulation of genetic methods (e.g., MR, and colocalization) and non-genetic methods (e.g., conventional multivariable regression,

For peer review only - http://bmjopen.bmj.com/site/about/guidelines.xhtml

negative paternal controls, and within-family analyses) to explore the effect of a range of maternal modifiable lifestyle factors, such as smoking, alcohol intake, coffee consumption, adiposity, sleeping habits, and physical activity, on the risk of multiple APPOs (**Figure 2**) (**Supplementary Table 1**) [29].

Aim 2: Better understanding the effect of maternal predisposition to conditions, such as autoimmune, cardiovascular, hormonal, musculoskeletal, and mental health conditions, on APPOs

An increasing number of women begin pregnancy with one or more pre-existing conditions that may elevate the risk of multiple APPOs [12]. A systematic understanding of the overall impact of these conditions on APPO risk is important for guiding clinical decision-making in the management and treatment of such conditions during pregnancy. Within the MR-PREG collaboration, we employ MR to investigate how genetic predisposition to common conditions in women of reproductive age - such as autoimmune, cardiovascular, hormonal, musculoskeletal, and mental health conditions - affects APPO risk (**Figure 2**).

Aim 3: Leveraging genomics to explore drug efficacy and safety of medications in pregnancy

The MR-PREG collaboration aims to improve the evidence on the benefits and risks of medications during pregnancy using drug target MR. We investigate potential effects of medications for mothers and their babies by: a) investigating the efficacy and safety in pregnancy of medications used to treat pre-existing conditions, b) discovering new candidate drug targets to prevent/treat APPOs, and c) identifying opportunities to repurpose existing drugs, designed to treat conditions unrelated to pregnancy, to prevent/treat APPOs (**Figure 2**).

Collaboration description

Adverse pregnancy and perinatal outcomes (APPOs)

A key focus of the MR-PREG collaboration is on assessing the causal effect of exposures on multiple outcomes simultaneously, so that we have a more complete picture of the potential beneficial, detrimental, or null effect of an exposure on APPOs. This ‘outcome-wide epidemiology’ approach [30] is informative so that women, their partners and healthcare

For peer review only - http://bmjopen.bmj.com/site/about/guidelines.xhtml

providers have a more balanced view of the potential adverse and beneficial effects of risk factors than what might be obtained from studies that focus on a single or small number of outcomes.

We have included 35 related binary or categorical outcomes that can occur in pregnancy, delivery, or the first year postpartum, as well as two underlying continuous traits. **Table 1** presents the number of women with both phenotypic and genetic data for each APPO, as well as the number of cases for binary outcomes. APPOs definitions and exclusion criteria are provided in **Supplementary Tables 2A** (binary outcomes) and **2B** (categorical or continuous outcomes). Study-specific sample sizes for binary and categorical APPOs are reported in **Supplementary Table 3A** (all women with APPO data across core studies), **Supplementary Table 3B** (women with APPO and maternal genetic data across all studies), and **Supplementary Table 3C** (women with APPO and linked fetal genetic data). Study-specific sample size, means and standard deviations for the continuous traits are reported in **Supplementary Table 3D** and **Supplementary Table 3E** (sample sizes for women with APPO and linked fetal genetic data). Any study-specific deviations from the collaboration definition for APPOs is described in **Supplementary Tables 4A** and **4B**.

Data sources

Currently, the MR-PREG collaboration includes data from four core studies, which comprise three prospective birth cohorts [the Avon Longitudinal Study of Parents and Children (ALSPAC), Born in Bradford (BiB), and the Norwegian Mother, Father and Child Cohort Study (MoBa)], and a biobank [UK Biobank (UKB)]. Data from additional sources are also used, such as genetic association data from publicly available biobanks (FinnGen) and genome-wide association studies (GWAS) meta-analyses, described below.

Core studies

A brief description of each of the four core studies is presented below and the participant characteristics are summarised in **Table 2**. **Supplementary Figures 1-4** show the flow of participants from recruitment into their cohorts to inclusion in our analyses involving maternal genetic and APPOs data. The APPOs each study contributed to MR-PREG are summarised in **Supplementary Tables 2A** and **2B**. Details on genotyping in each study are presented in supplementary material.

For peer review only - http://bmjopen.bmj.com/site/about/guidelines.xhtml

ALSPAC: The Avon Longitudinal Study of Parents and Children

The Avon Longitudinal Study of Parents and Children (ALSPAC) is a prospective birth cohort that started recruiting pregnant women resident in the former county of Avon (centred around the city of Bristol), with delivery dates between April 1991 and December 1992. A total of 14,541 women (ALSPAC-G0) were enrolled during pregnancy (14,676 fetuses) and gave birth to 14,062 live children (ALSPAC-G1) [31 32]. Women responded to four questionnaires during pregnancy (average 8, 12, 18, and 32 weeks' gestation) and two postpartum (average 8 weeks and 8 months). Biological samples were collected during pregnancy (blood and urine) and birth (cord blood and placenta). Maternal anthropometrics was based on self-report collected at 12 weeks' gestation and children had anthropometrics measured at birth. Obstetric records were also linked to the participants. Genetic data is available for mothers, partners and children (details on genotyping array and imputation are presented in supplementary material). Children of the children (ALSPAC-G2) are also being assessed and followed-up, but this data has not yet been included in the MR-PREG collaboration. The study website contains details of all the data available through a fully searchable data dictionary and variable search tool: http://www.bristol.ac.uk/alspac/researchers/our-data/. Ethical approval for the study was obtained from the ALSPAC Ethics and Law Committee and the Local Research Ethics Committees. Full details of the ALSPAC consent procedures are available on the study website (http://www.bristol.ac.uk/alspac/researchers/research-ethics/http://www.bristol.ac.uk/alspac/researchers/research-ethics/).

BiB: Born in Bradford

Born in Bradford (BiB) is a prospective birth cohort that recruited women with expected delivery dates between March 2007 and December 2010. Most women were recruited at their oral glucose tolerance test (OGTT) at approximately 26–28 weeks' gestation, which was offered to all women booked for delivery at Bradford Royal Infirmary, except those with known diabetes, during the recruitment period. In BiB, most of the obstetric population consists of women of White British or Pakistani origin (together accounting for 81%, with the remaining women being of other ancestries). A total of 12,453 women (13,776 pregnancies) were enrolled during pregnancy who gave birth to 13,858 live children. Women had anthropometrics measured at recruitment and responded to questionnaires during pregnancy (~28 weeks) and postpartum (6, 12 and 18 months). BiB participants have been

For peer review only - http://bmjopen.bmj.com/site/about/guidelines.xhtml

linked to maternity health records, hospital admission data and primary care data for both mother and child. Biological samples were collected during pregnancy (blood and urine) and birth (cord blood). Full details of the study methodology were reported previously [33]. Genetic data is available for mothers and children (details on genotyping array and imputation presented in supplementary material). Ethical approval for the study was granted by the Bradford National Health Service Research Ethics Committee (ref 06/Q1202/48). The study website provides further cohort details and an overview of available data (https://borninbradford.nhs.uk/).

MoBa: The Norwegian Mother, Father and Child Cohort Study

The Norwegian Mother, Father and Child Cohort Study (MoBa) is a prospective birth cohort that recruited pregnant women from all over Norway from 1999-2008 [34 35]. The cohort includes approximately 95,200 mothers, 75,200 fathers, and 114,500 children. Mothers and their partners responded to questionnaires during pregnancy (15, 22, and 30 weeks' gestation) and at multiple times postpartum (starting from when the child was 6 months old). Blood samples were obtained from both parents during pregnancy and from mothers and children (cord blood) at birth. Maternal anthropometrics were collected from a questionnaire at 15 weeks' gestation. Data was linked to the Medical Birth Registry (MBRN), which is a national health registry containing information about all births in Norway. Genetic data is available for mothers, children and partners (details on genotyping array and imputation presented in supplementary material). The establishment of MoBa and initial data collection was based on a license from the Norwegian Data Protection Agency and approval from The Regional Committees for Medical and Health Research Ethics. The MoBa cohort is currently regulated by the Norwegian Health Registry Act. Ethical approval for our study was obtained from The Regional Committees for Medical and Health Research Ethics (ref 2018/1256).

UKB: UK Biobank

UK Biobank (UKB) is an adult cohort that retrospectively collected relevant data on APPOs. All people in the UK National Health Service (NHS) registry aged between 40-69 years and living within an approximately 25-mile radius from one of the 22 study centres were invited to participate in UKB between 2006-2010 [36 37]. A total of 500,000 adults (5.5% of the ~9.2 million invited) were recruited into the study (54.4% females). Information was assessed at baseline

For peer review only - http://bmjopen.bmj.com/site/about/guidelines.xhtml

via a self-completed questionnaire, physical measures (including anthropometrics), and collection of non-fasting blood, urine, and saliva. Participants have been followed up by linkage to electronic health records and a subset of participants responded to online questionnaires. Hospital Episode Statistics (HES) from 1997 for England, 1998 for Wales and 1981 for Scotland are available [38]. HES data also contain maternity-related admissions for England and Wales. It is important to note that not every participant has a hospital inpatient record, as not all have been admitted to hospital within the period covered. Genetic data from UKB participants is available (details on genotyping array and imputation presented in supplementary material). Ethical approval for UKB was obtained from the Northwest Multi-Centre Research Ethics Committee (MREC), and MR-PREG collaboration studies are linked to UKB application number 23938. The UKB showcase website contains details of the data available: https://biobank.ndph.ox.ac.uk/showcase/.

Additional data sources

To increase statistical power for MR analyses, the MR-PREG collaboration also uses genetic association data for APPOs from publicly available datasets – i.e., FinnGen and several publicly available GWAS meta-analyses – as described below.

FinnGen

FinnGen is a nationwide network of Finnish biobanks linked to national electronic health registries that provide information on prescriptions and disease diagnoses (ICD-9 and ICD-10 codes).[39] The current FinnGen release (R12) includes data from 500,348 individuals (282,064 females and 218,284 males). In addition to clinical endpoints, including several APPOs, genetic data are also available. The APPOs and corresponding numbers of cases and controls contributed by FinnGen are provided in **Supplementary Table 3B**. FinnGen has been approved by the Coordinating Ethics Committee of the Helsinki and Uusimaa Hospital District (Nr HUS/990/2017). Further information on FinnGen is available at https://www.finngen.fi/en. The metadata used by the MR-PREG collaboration can be accessed at https://www.finngen.fi/en/access_results.

Publicly available GWAS meta-analyses

For peer review only - http://bmjopen.bmj.com/site/about/guidelines.xhtml

At the time of writing, the MR-PREG collaboration has harmonised and quality-controlled data from GWAS meta-analyses for GDM [5,485 cases and 347,856 controls from the GENetics of Diabetes in Pregnancy Consortium (GenDIP) consortium [40]]; PE [9,515 cases and 157,719 controls from the International Pregnancy Genetics (InterPregGen) consortium [41]]; gestational duration-related traits [18,797 PTB cases and 260,246 controls, 15,972 post-term birth cases and 115,307 controls, and 195,555 individuals with gestational age at delivery from the Early Growth Genetics (EGG) consortium [42]]; post-natal depression [17,339 cases and 53,426 controls from the Psychiatric Genomics consortium (PGC) [43]]. Details are provided in **Supplementary Table 5**, including the number of participants, outcome definition and source of data (e.g., electronic health records, maternal report, research data collection), and availability of maternal and/or fetal genetic effects.

Genetic association data for adverse pregnancy and perinatal outcomes

For studies with access to individual-level data (ALSPAC, BiB, MoBa, and UKB), we conducted GWAS analyses to generate genetic association data for APPOs, enabling two-sample MR analyses across the aims of the MR-PREG collaboration. The procedures used for conducting GWAS and quality control in each study are described in detail in the **Supplementary text**. We excluded genetic variants with low imputation accuracy (INFO score < 0.4) and/or low minor allele frequency (MAF < 0.01). In addition, we excluded studies that overlapped with public GWAS metanalyses or that contributed fewer than 50 cases for a given APPO, and applied genomic control to each study. We then pooled study-specific genetic association data on APPOs using inverse variance weighted, fixed-effects meta-analyses implemented in METAL (v. 2020-05-05) [44] and estimated Cochrane's Q statistic to explore between-study heterogeneity. Separate meta-analyses were conducted for maternal and offspring genetic effects. We used GWASInspector to evaluate the quality of both study-level and meta-analytic GWAS summary data[45].

Decomposing Maternal and Fetal Genetic Contributions to APPOs

The MR-PREG collaboration is primarily interested in causal effects of maternal exposures on APPOs. Due to the correlation between maternal and offspring genotype, accounting for offspring genetic effects is crucial for reliable interpretation of MR findings of maternal

For peer review only - http://bmjopen.bmj.com/site/about/guidelines.xhtml

exposure effects (**Box 1, Figure 3a** and **Figure 3b**). Genetically instrumented maternal exposure effects (not biased due to exclusion restriction violation via fetal effects) and genetically instrumented fetal effects (not confounded by maternal genotype) can be estimated. We used a weighted linear model (WLM) implemented in the DONUTS (Decomposing nature and nurture using GWAS summary statistics) software [46] to estimate mutually adjusted maternal and fetal genetic effects at each SNP for each APPO. The WLM calculates conditional genetic effects on APPOs (i.e., the mutually adjusted coefficients for maternal, offspring and paternal genotype, fitted jointly in the same model) as linear combinations of the marginal genetic effects (i.e., the coefficients for maternal, offspring and paternal genotype, estimated separately in potentially overlapping samples). We created two sets of conditional genetic effects estimates on APPOs. The first corresponded to mutually adjusted maternal and fetal estimates, leveraging data from the maternal and fetal GWAS metanalyses. The second corresponded to mutually adjusted maternal, fetal and paternal estimates, leveraging data from maternal and fetal GWAS metanalyses, as well as paternal GWAS conducted in MoBa. Details can be found in **Supplementary text.**

Patient, Participant and Public Involvement (PPI)

The birth cohorts involved in this collaboration (ALSPAC[47], BiB[33] and MoBa[35]) have a strong track record of PPI. For example, in ALSPAC since 2021 participants who have become pregnant, or a parent, and their children have been invited to be involved in the ALSPAC G2 cohort[48]. PPI with them identified new data collection that have informed this collaboration, such as understanding the potential effects of vaping, mental health and climate change on APPOs. Similarly, the current offspring of BiB, although young for starting pregnancy (currently aged 14 to 18), have been involved in designing the current follow-up that will go into their early 20s, and highlighted reproductive health as an important area for research. The design of the MoBa study, the recruitment procedures, the aims of the study, and the research questions were discussed during the 1990's with the Norwegian Society for Gynecology and Obstetrics, international colleagues, and at national meetings with midwives. The study was also discussed in media and in the Norwegian Parliament before it became a national study.

For peer review only - http://bmjopen.bmj.com/site/about/guidelines.xhtml

Findings to date

To date there have been 11 peer-reviewed publications [23 49-58] and 2 preprints [59 60] resulting from the MR-PREG collaboration. We briefly describe a selection of these here to give readers an indication of the different ways in which MR-PREG can be used to explore effects of exposures on APPOs.

Most publications to date align with **Aim 1**, enhancing our understanding of how maternal lifestyle factors influence the risk of APPOs, thereby informing interventions aimed at their prevention. For example, by triangulating evidence from MR, conventional multivariable regression and paternal negative control, we have shown that higher maternal BMI increases the risk of GH, PE, GDM, pre-labour membrane rupture, induction of labour, Caesarean section, LGA, high birthweight, low Apgar score, and admission to a neonatal intensive care unit. We also found evidence of higher maternal BMI reducing the odds of SGA and breastfeeding and having no detectable effect on perinatal depression [52]. Triangulating evidence from MR and conventional multivariable regression, we found evidence that insomnia may increase the risk of perinatal depression but does not appear to influence most other APPOs that we were able to explore [54]. In a study focused on fetal growth trajectories, assessed by repeat ultrasound scan measures in two cohorts, we triangulated evidence from MR, conventional multivariable regression and paternal negative control study and found evidence of a consistent linear dose-response association of maternal smoking with fetal growth from early in the second trimester onwards. No major growth deficit was found in women who quit smoking early in pregnancy [57].

In relation to **Aim 3**, we have published preliminary work identifying novel molecular targets (i.e., proteins and metabolites) causally related to APPOs. In one study, we used MR to investigate the effect of 1,139 maternal and fetal circulating proteins on offspring birthweight. Results support maternal and fetal protein effects on birthweight, implicating roles for glucose metabolism, energy balance, and vascular function. [59] For example, we found evidence that higher maternal levels of PCSK1 potentially increase birthweight whilst higher maternal levels of LGALS4 potentially decrease birthweight. In contrast, fetal genetically predicted PCSK1 and LGALS4 showed effects in the opposite direction. We have also identified

For peer review only - http://bmjopen.bmj.com/site/about/guidelines.xhtml

maternal circulating metabolites beyond glucose that may influence birthweight, such as amino acids, including glutamine[49]. In further studies, we used conventional multivariable regression and MR to explore the potential effect of > 1,000 maternal circulating metabolites on the risk of offspring congenital heart disease. We found that pregnancy amino acid metabolism, androgenic steroid lipids, and levels of succinylcarnitine may be contributing factors for congenital heart disease (CHD) [53].

Strengths and Limitations

The collaboration has created curated data for 35 APPOs and harmonised data across several studies to support large-scale investigations of causes of APPOs. In addition, the generation of genetic association data enables well-powered genetic studies, including MR studies, to improve causal knowledge of many key targets for lifestyle and pharmaceutical interventions aimed at preventing APPOs. Data on equivalent exposures in partners and on a wide range of plausible confounders, enables triangulation across genetic and non-genetic approaches, and also pertinent sensitivity analyses. In relation to those sensitivity analyses, the collaboration has also derived conditional estimates so that maternal genetic variants can be used as an instrument to test the effect of maternal exposures without potential biases related to offspring genetic effects.

Despite the size and scale of the data, we are still underpowered to detect causal effects on rare APPOs, such as congenital anomalies and low Apgar scores. Furthermore, participants are of predominantly European ancestry, except for BiB. Enhancing the ancestry diversity in the data contributing to the collaboration is a key priority as outlined below in 'Collaborations and future plans'. Finally, despite our best efforts to derive accurate and standardised APPO definitions, there is inevitably a considerable degree of misclassification, especially where some studies derived their data only from self-reported information or only from electronic health records.

Collaborations and future plans

We are currently focussing on systematically assessing the effects of predisposition to autoimmune, mental health, thyroid, reproductive, and cardiometabolic disorders on APPOs

For peer review only - http://bmjopen.bmj.com/site/about/guidelines.xhtml

(**Aim 2**), discovering new candidate drug targets for APPOs (**Aim 3**), and investigating the efficacy, safety and potential of repurposing existing drugs during pregnancy (**Aim 3**).

A future priority is to increase diversity in the genetic background of MR-PREG collaboration participants, given the current effort is predominantly focussed on participants of European ancestry. This will enable us to improve the internal validity of the MR-PREG studies (e.g., leveraging trans-ancestral data to mitigate challenges due to linkage disequilibrium) but also external validity (e.g., testing transportability of estimated effects across ancestries).

Additionally, we are incorporating more molecular data into MR-PREG studies to better understand the molecular mechanisms underlying APPOs, for example, placental transcriptomics and proteomics. To enhance our work identifying molecular targets for the prevention and treatment of APPOs (**Aim 3**), we are utilizing whole-exome and whole-genome sequencing data to pinpoint causal genes linked to these adverse outcomes.

We are advancing collaborations with additional large-scale studies including genetic and APPO data, with a focus on rarer outcomes such as congenital anomalies and participants of non-European ancestry to increase statistical power for key safety outcomes and ancestry diversity.

Data availability

Analytical code used in the MR-PREG collaboration can be accessed at https://github.com/MRCIEU/MR-PREG.

Genetic association data for APPOs generated by the MR-PREG collaboration can only be used for research that is covered by data agreements with current contributing studies.

The ALSPAC access policy that describes the proposal process in detail including any costs associated with conducting research at ALSPAC, which may be updated from time to time, and is available at: https://www.bristol.ac.uk/medialibrary/sites/alspac/documents/researchers/dataaccess/ALSPAC_Access_Policy.pd

Data is available upon request from BiB, and information is available at: https://borninbradford.nhs.uk/research/how-to-access-data/

For peer review only - http://bmjopen.bmj.com/site/about/guidelines.xhtml

Data from MoBa are available upon application to Helsedata administered by the Norwegian Institute of Public Health (see its website https://www.fhi.no/en/ch/studies/moba/for-forskere-artikler/research-and-data-access/#:~:text=Relevant%20information%20for%20researchers%20applying%20for%20access%20to,be%20submitted%20via%20the%20application%20form%20on%20helsedata.no. for details).

Researchers can apply for access to the UK Biobank data via the Access Management System (AMS) (https://www.ukbiobank.ac.uk/enable-your-research/apply-for-access).

Author contributions

The MR-PREG collaboration was conceptualised by DAL and MCB. GC, NM, AGS, MCB, QY, TB, CC, EA, AT undertook data curation and generated GWAS pipelines for the individual cohorts. QY and MCB ran the meta-analysis. TB and MCB generated the WLM models. GC, NM, MCB, AGS and DAL wrote the paper, and all authors edited and approved the manuscript. This publication is the work of the authors and MCB acts as guarantors for the contents of this paper.

Acknowledgements

The authors would like to thank Dr Marwa Al-Arab, Dr Alba Fernandez-Sanles, Dr Helena Urquijo, Jevvy Huang, and Peiyuan Huang for their assistance with data preparation and statistical analysis. The authors also thank Professor Dave Evans for the valuable discussions regarding the implementation of WLM models to estimate conditional genetic effects.

All cohort specific acknowledgements are detailed in the **Supplementary text**.

Funding declaration

All cohort specific funding information is detailed in the **Supplementary text**.

DAL, MCB, GC, AGS, TB, QY, AT, HC, EA, PH, and NM work in a unit supported by the University of Bristol and UK Medical Research Council (MC_UU_00032/5) and the British Heart Foundation (AA/18/1/34219). AGS was supported by the European Union's Horizon 2020 research and innovation programme (grant agreement No 874739, LongITools). AGS, GC and DAL are supported by STAGE that has received funding from the European Union's

For peer review only - http://bmjopen.bmj.com/site/about/guidelines.xhtml

Horizon Europe Research and Innovation Programme under grant agreement nº 101137146 (via UKRI grant number 10112787 (Beta Technology) 10099041). EA is supported by a Wellcome Trust PhD studentship (228276/Z/23/Z). MCM is supported by the Research Council of Norway through its Centres of Excellence funding scheme (project No 262700) and the European Research Council under the European Union's Horizon 2020 research and innovation program (ERC Starting Grant, INFERTILITY grant agreement No 947684). JW was funded for BiB data collection by a UK Medical Research Council (MRC) Special Training Fellowship in Health of the Public and Health Services Research (MRC GO601712) and is now supported by supported by the UK Prevention Research Partnership (MR/S037527/1), an initiative funded by UK Research and Innovation Councils, the Department of Health and Social Care (England) and the UK devolved administrations, and leading health research charities.

Conflict of interest

All authors declare no conflict of interest.

For peer review only

For peer review only - http://bmjopen.bmj.com/site/about/guidelines.xhtml

References

1. Quenby S, Gallos ID, Dhillon-Smith RK, et al. Miscarriage matters: the epidemiological, physical, psychological, and economic costs of early pregnancy loss. *The Lancet* 2021;397(10285):1658-67. doi: 10.1016/S0140-6736(21)00682-6.
2. Wang H, Li N, Chivese T, et al. IDF Diabetes Atlas: Estimation of Global and Regional Gestational Diabetes Mellitus Prevalence for 2021 by International Association of Diabetes in Pregnancy Study Group's Criteria. *Diabetes Research and Clinical Practice* 2022;183:109050. doi: 10.1016/j.diabres.2021.109050.
3. Abalos E, Cuesta C, Grosso AL, et al. Global and regional estimates of preeclampsia and eclampsia: a systematic review. *European Journal of Obstetrics and Gynecology and Reproductive Biology* 2013;170(1):1-7. doi: 10.1016/j.ejogrb.2013.05.005.
4. Ohuma EO, Moller AB, Bradley E, et al. National, regional, and global estimates of preterm birth in 2020, with trends from 2010: a systematic analysis. *The Lancet* 2023;402(10409):1261-71. doi: 10.1016/s0140-6736(23)00878-4.
5. Souza JP, Day LT, Rezende-Gomes AC, et al. A global analysis of the determinants of maternal health and transitions in maternal mortality. *The Lancet Global Health* 2024;12(2):e306-e16. doi: 10.1016/S2214-109X(23)00468-0.
6. Geller SE, Koch AR, Garland CE, et al. A global view of severe maternal morbidity: moving beyond maternal mortality. *Reproductive Health* 2018;15(1):98. doi: 10.1186/s12978-018-0527-2.
7. Catov JM, Margerison-Zilko C. Pregnancy as a window to future health: short-term costs and consequences. *American Journal of Obstetrics and Gynecology* 2016;215(4):406-7. doi: 10.1016/j.ajog.2016.06.060.
8. Rich-Edwards JW, Fraser A, Lawlor DA, et al. Pregnancy Characteristics and Women's Future Cardiovascular Health: An Underused Opportunity to Improve Women's Health? *Epidemiologic Reviews* 2014;36(1):57-70. doi: 10.1093/epirev/mxt006.
9. Brito Nunes C, Borges MC, Freathy RM, et al. Understanding the Genetic Landscape of Gestational Diabetes: Insights into the Causes and Consequences of Elevated Glucose Levels in Pregnancy. *Metabolites* 2024;14(9) doi: 10.3390/metabo14090508.
10. Wu P, Green M, Myers JE. Hypertensive disorders of pregnancy. *BMJ* 2023;381:e071653. doi: 10.1136/bmj-2022-071653.

For peer review only - http://bmjopen.bmj.com/site/about/guidelines.xhtml

11. Angum F, Khan T, Kaler J, et al. The Prevalence of Autoimmune Disorders in Women: A Narrative Review. *Cureus* 2020;12(5):e8094. doi: 10.7759/cureus.8094.

12. Singh M, Wambua S, Lee SI, et al. Autoimmune diseases and adverse pregnancy outcomes: an umbrella review. *BMC Medicine* 2024;22(1):94. doi: 10.1186/s12916-024-03309-y.

13. Molenaar NM, Bais B, Lambregtse-van den Berg MP, et al. The international prevalence of antidepressant use before, during, and after pregnancy: A systematic review and meta-analysis of timing, type of prescriptions and geographical variability. *Journal of Affective Disorders* 2020;264:82-89. doi: 10.1016/j.jad.2019.12.014.

14. Conrad N, Molenberghs G, Verbeke G, et al. Trends in cardiovascular disease incidence among 22 million people in the UK over 20 years: population based study. *BMJ* 2024;385:e078523. doi: 10.1136/bmj-2023-078523.

15. Subramanian A, Azcoaga-Lorenzo A, Anand A, et al. Polypharmacy during pregnancy and associated risk factors: a retrospective analysis of 577 medication exposures among 1.5 million pregnancies in the UK, 2000-2019. *BMC Medicine* 2023;21(1):21. doi: 10.1186/s12916-022-02722-5.

16. Academy of Medical Sciences. Understanding pregnancy: Accelerating the development of new therapies for pregnancy specific conditions, 2023.

17. National Academies of Sciences, Engineering, and Medicine. Advancing Clinical Research with Pregnant and Lactating Populations: Overcoming Real and Perceived Liability Risks, 2024.

18. UK Commission on Human Medicines. Report of the Commission on Human Medicines Expert Working Group on Optimising Data on Medicines used During Pregnancy, 2021.

19. Smith GD, Ebrahim S. 'Mendelian randomization': can genetic epidemiology contribute to understanding environmental determinants of disease? *International Journal of Epidemiology* 2003;32(1):1-22. doi: 10.1093/ije/dyg070.

20. Lawlor DA, Harbord RM, Sterne JA, et al. Mendelian randomization: using genes as instruments for making causal inferences in epidemiology. *Statistics in Medicine* 2008;27(8):1133-63. doi: 10.1002/sim.3034.

21. Zheng J, Baird D, Borges MC, et al. Recent Developments in Mendelian Randomization Studies. *Current Epidemiology Reports* 2017;4(4):330-45. doi: 10.1007/s40471-017-0128-6.

For peer review only - http://bmjopen.bmj.com/site/about/guidelines.xhtml

22. Yan M, Chen Z, Tang J, et al. Association between gestational diabetes mellitus and offspring health: a two-sample mendelian randomization study. *BMC Pregnancy and Childbirth* 2025;25(1):321. doi: 10.1186/s12884-025-07423-4.

23. Taylor K, Wootton RE, Yang Q, et al. The effect of maternal BMI, smoking and alcohol on congenital heart diseases: a Mendelian randomisation study. *BMC Medicine* 2023;21(1):35. doi: 10.1186/s12916-023-02731-y.

24. Tyrrell J, Richmond RC, Palmer TM, et al. Genetic Evidence for Causal Relationships Between Maternal Obesity-Related Traits and Birth Weight. *JAMA* 2016;315(11) doi: 10.1001/jama.2016.1975.

25. Schmidt AF, Finan C, Gordillo-Marañón M, et al. Genetic drug target validation using Mendelian randomisation. *Nature Communications* 2020;11(1):3255. doi: 10.1038/s41467-020-16969-0.

26. Holmes MV, Richardson TG, Ference BA, et al. Integrating genomics with biomarkers and therapeutic targets to invigorate cardiovascular drug development. *Nature Reviews Cardiology* 2021;18(6):435-53. doi: 10.1038/s41569-020-00493-1.

27. Zheng J, Haberland V, Baird D, et al. Phenome-wide Mendelian randomization mapping the influence of the plasma proteome on complex diseases. *Nature Genetics* 2020;52(10):1122-31. doi: 10.1038/s41588-020-0682-6.

28. Atia A, Aboeldahab H, Wageeh A, et al. Safety and Efficacy of Proprotein Convertase Subtilisin-Kexin Type 9 Inhibitors After Acute Coronary Syndrome: A Meta-analysis of Randomized Controlled Trials. *American Journal of Cardiovascular Drugs* 2024;24(1):83-102. doi: 10.1007/s40256-023-00621-5.

29. Lawlor DA, Tilling K, Davey Smith G. Triangulation in aetiological epidemiology. *International Journal of Epidemiology* 2016;45(6):1866-86. doi: 10.1093/ije/dyw314.

30. VanderWeele TJ. Outcome-wide Epidemiology. *Epidemiology* 2017;28(3):399-402. doi: 10.1097/ede.0000000000000641.

31. Boyd A, Golding J, Macleod J, et al. Cohort Profile: the ‘children of the 90s’ – the index offspring of the Avon Longitudinal Study of Parents and Children. *International Journal of Epidemiology* 2013;42 doi: 10.1093/ije/dys064.

32. Fraser A, Macdonald-Wallis C, Tilling K, et al. Cohort profile: The Avon Longitudinal Study of Parents and Children: ALSPAC mothers cohort. *International Journal of Epidemiology* 2013;42 doi: 10.1093/ije/dys066.

For peer review only - http://bmjopen.bmj.com/site/about/guidelines.xhtml

33. Wright J, Small N, Raynor P, et al. Cohort Profile: the Born in Bradford multi-ethnic family cohort study. *International Journal of Epidemiology* 2013;42(4):978-91. doi: 10.1093/ije/dys112.

34. Magnus P, Birke C, Vejrup K, et al. Cohort Profile Update: The Norwegian Mother and Child Cohort Study (MoBa). *International Journal of Epidemiology* 2016;45(2):382-8. doi: 10.1093/ije/dyw029.

35. Magnus P, Irgens LM, Haug K, et al. Cohort profile: the Norwegian Mother and Child Cohort Study (MoBa). *International Journal of Epidemiology* 2006;35(5):1146-50. doi: 10.1093/ije/dyl170.

36. Littlejohns TJ, Sudlow C, Allen NE, et al. UK Biobank: opportunities for cardiovascular research. *European Heart Journal* 2019;40(14):1158-66. doi: 10.1093/eurheartj/ehx254.

37. Sudlow C, Gallacher J, Allen N, et al. UK biobank: an open access resource for identifying the causes of a wide range of complex diseases of middle and old age. *PLoS Med* 2015;12(3):e1001779. doi: 10.1371/journal.pmed.1001779.

38. UK Biobank. UK Biobank hospital inpatient data. Version 4.0. 2023 [Available from: https://biobank.ctsu.ox.ac.uk/crystal/crystal/docs/HospitalEpisodeStatistics.pdf.

39. Kurki MI, Karjalainen J, Palta P, et al. FinnGen provides genetic insights from a well-phenotyped isolated population. *Nature* 2023;613(7944):508-18. doi: 10.1038/s41586-022-05473-8.

40. Pervjakova N, Moen GA-O, Borges MC, et al. Multi-ancestry genome-wide association study of gestational diabetes mellitus highlights genetic links with type 2 diabetes. *Human Molecular Genetics* 2022;31(19) doi: 10.1093/hmg/ddac050.

41. Steinthorsdottir V, McGinnis R, Williams NO, et al. Genetic predisposition to hypertension is associated with preeclampsia in European and Central Asian women. *Nature Communications* 2020;11(1):5976. doi: 10.1038/s41467-020-19733-6.

42. Solé-Navais P, Flatley C, Steinthorsdottir V, et al. Genetic effects on the timing of parturition and links to fetal birth weight. *Nature Genetics* 2023;55(4):559-67. doi: 10.1038/s41588-023-01343-9.

43. Guintivano J, Byrne EM, Kiewa J, et al. Meta-Analyses of Genome-Wide Association Studies for Postpartum Depression. *American Journal of Psychiatry* 2023;180(12):884-95. doi: 10.1176/appi.ajp.20230053.

For peer review only - http://bmjopen.bmj.com/site/about/guidelines.xhtml

44. Willer CJ, Li Y, Abecasis GR. METAL: fast and efficient meta-analysis of genomewide association scans. *Bioinformatics* 2010;26(17):2190-1. doi: 10.1093/bioinformatics/btq340.

45. Ani A, van der Most PJ, Snieder H, et al. GWASinspector: comprehensive quality control of genome-wide association study results. *Bioinformatics* 2021;37(1):129-30. doi: 10.1093/bioinformatics/btaa1084.

46. Wu Y, Zhong X, Lin Y, et al. Estimating genetic nurture with summary statistics of multigenerational genome-wide association studies. *Proceedings of the National Academy of Sciences* 2021;118(25):e2023184118. doi: doi:10.1073/pnas.2023184118.

47. Northstone K, Lewcock M, Groom A, et al. The Avon Longitudinal Study of Parents and Children (ALSPAC): an update on the enrolled sample of index children in 2019. *Wellcome Open Research* 2019;4:51. doi: 10.12688/wellcomeopenres.15132.1.

48. Lawlor DA, Lewcock M, Rena-Jones L, et al. The second generation of The Avon Longitudinal Study of Parents and Children (ALSPAC-G2): a cohort profile. *Wellcome Open Research* 2019;4:36. doi: 10.12688/wellcomeopenres.15087.2.

49. Zhao J, Stewart ID, Baird D, et al. Causal effects of maternal circulating amino acids on offspring birthweight: a Mendelian randomisation study. *eBioMedicine* 2023;88 doi: 10.1016/j.ebiom.2023.104441.

50. Barry CA-O, Lawlor DA, Shapland CA-O, et al. Using Mendelian Randomisation to Prioritise Candidate Maternal Metabolic Traits Influencing Offspring Birthweight. *Metabolites* 2022;12(6) doi: 10.3390/metabo12060537.

51. Yang Q, Magnus MC, Kilpi F, et al. Investigating causal relations between sleep duration and risks of adverse pregnancy and perinatal outcomes: linear and nonlinear Mendelian randomization analyses. *BMC Medicine* 2022;20(1):295. doi: 10.1186/s12916-022-02494-y.

52. Borges MC, Clayton GL, Freathy RM, et al. Integrating multiple lines of evidence to assess the effects of maternal BMI on pregnancy and perinatal outcomes. *BMC Medicine* 2024;22(1):32. doi: 10.1186/s12916-023-03167-0.

53. Taylor K, McBride N, Zhao J, et al. The Relationship of Maternal Gestational Mass Spectrometry-Derived Metabolites with Offspring Congenital Heart Disease: Results from Multivariable and Mendelian Randomization Analyses. *Journal of Cardiovascular Development and Disease* 2022;9(8) doi: 10.3390/jcdd9080237.

For peer review only - http://bmjopen.bmj.com/site/about/guidelines.xhtml

54. Yang Q, Borges MC, Sanderson E, et al. Associations between insomnia and pregnancy and perinatal outcomes: Evidence from mendelian randomization and multivariable regression analyses. *PLOS Medicine* 2022;19(9):e1004090. doi: 10.1371/journal.pmed.1004090.

55. Yang Q, Sanderson E, Tilling K, et al. Exploring and mitigating potential bias when genetic instrumental variables are associated with multiple non-exposure traits in Mendelian randomization. *European Journal of Epidemiology* 2022;37(7):683-700. doi: 10.1007/s10654-022-00874-5.

56. Morales-Berstein F, Goncalves-Soares A, Yang Q, et al. Assessing the impact of maternal blood pressure during pregnancy on perinatal health: a wide-angled Mendelian randomization study. *BMC Medicine* 2026;24(1):2. doi: 10.1186/s12916-025-04548-3.

57. Brand JS, Gaillard R, West J, et al. Associations of maternal quitting, reducing, and continuing smoking during pregnancy with longitudinal fetal growth: Findings from Mendelian randomization and parental negative control studies. *PLOS Medicine* 2019;16(11):e1002972. doi: 10.1371/journal.pmed.1002972.

58. Yang Q, Magnus MC, Kilpi F, et al. Evaluating causal associations of chronotype with pregnancy and perinatal outcomes and its interactions with insomnia and sleep duration: a mendelian randomization study. *BMC Pregnancy Childbirth* 2024;24(1):816. doi: 10.1186/s12884-024-07023-8.

59. McBride N, Fernández-Sanlés A, Al Arab M, et al. Effects of the maternal and fetal proteome on birth weight: a Mendelian randomization analysis. *medRxiv* 2025 doi: 10.1101/2023.10.20.23297135.

60. Aiton E, McBride NS, Clayton GL, et al. Characterising the effects of genetic liability to autoimmune conditions on pregnancy outcomes using Mendelian Randomization. *medRxiv* 2025:2025.08.12.25333479. doi: 10.1101/2025.08.12.25333479.

61. Sanderson E, Glymour MM, Holmes MV, et al. Mendelian randomization. *Nat Rev Methods Primers* 2022;2 doi: 10.1038/s43586-021-00092-5.

For peer review only - http://bmjopen.bmj.com/site/about/guidelines.xhtml

Figure 1 Summary of the core Mendelian randomization assumptions to estimate the presence of an effect of maternal risk factors on adverse pregnancy and perinatal outcomes

Figure 2 MR-PREG collaboration aims

Box 1 Approaches to explore the plausibility of the Mendelian randomisation core assumptions (**Figure 1**), with a focus on the context of maternal risk factors and adverse pregnancy and perinatal outcomes (APPOs). For a review of standard MR sensitivity analyses, we refer readers to Sanderson et al.[61]

Figure 3a Illustration of how the exclusion restriction assumption may be violated; **Figure 3b** Illustration of how collider bias might be induced in MR analysis when conditioning on offspring genetic variants.

Offspring genetic variation might violate the exclusion restriction assumption (**Figure 3a**). Adjusting for offspring genetic variants only (which are a collider variables) (**Figure 3b**) means that a false association between maternal and paternal genetic variants can be created (shown as a dashed line). Such associations can also arise via assortative mating. This will only cause bias if the outcome is influenced by paternal genetic variants, independently of maternal and offspring genetic variants. However, by additionally adjusting for paternal genetic variants, the pathway highlighted in black dashed arrows can be closed.

For peer review only

For peer review only - http://bmjopen.bmj.com/site/about/guidelines.xhtml

Table 1. Adverse pregnancy and perinatal outcomes (APPOs) and underlying traits, total available sample and respective number of cases

Type	Outcomes/Traits	Total	Cases N (%)
Pregnancy	Hypertensive disorders of pregnancy	541,768	32,549 (6.0%)
	Gestational hypertension	527,932	20,777 (3.9%)
	Pre-eclampsia	671,992	19,408 (2.9%)
	Gestational diabetes	707,797	24,641 (3.5%)
	Perinatal depression	105,656	14,591 (13.8%)
	Anaemia	86,167	4,105 (4.8%)
	Pregnancy loss	298,868	94,152 (31.5%)
	Miscarriage	486,217	89,086 (18.3%)
	Sporadic miscarriage	241,159	55,888 (23.2%)
	Recurrent miscarriage	321,756	6,233 (1.9%)
	Stillbirth	207,670	6,331 (3.0%)
	Hyperemesis	478,466	4,235 (0.9%)
	Severity of nausea and vomiting	82,978	
	Nausea only		7,166 (8.6%)
	Nausea and vomiting		30,133 (36.3%)
	On medication		31,210 (37.6%)
Delivery	Induction of labour	95,239	14,495 (15.2%)
	Premature rupture of membranes	310,621	21,839 (7.0%)
	Caesarean section	233,969	33,909 (14.5%)
	Emergency C-section	90,438	9,165 (10.1%)
	Elective C-section	87,283	6,004 (6.9%)
	Very pre-term birth	77,683	1,107 (1.4%)
	Pre-term birth	285,722	18,225 (6.4%)

For peer review only - http://bmjopen.bmj.com/site/about/guidelines.xhtml

	Spontaneous pre-term birth	272,940	17,176 (6.3%)
	Post-term birth	414,667	27,213 (6.6%)
	Low birth weight	283,151	19,180 (6.8%)
	High birth weight	266,835	6,679 (2.5%)
	Small for gestational age	96,305	7,448 (7.7%)
	Large for gestational age	96,305	10,468 (10.9%)
	Low Apgar score at 1 minute	86,668	4,950 (5.7%)
	Low Apgar score at 5 minutes	74,368	821 (1.1%)
Postnatal	NICU admission	77,285	6,996 (9.1%)
	Congenital anomalies	74,701	3,569 (4.8%)
	Congenital heart disease	74,701	612 (0.8%)
	Breastfeeding initiation	84,668	66,408 (78.4%)
	Breastfeeding established	81,258	58,035 (71.4%)
	Breastfeeding sustained	65,189	44,401 (68.1%)
	Breastfeeding duration	82,429	
	1 to 3		6,372 (7.7%)
	4 to 6		49,123 (59.6%)
	>6		3,419 (4.1%)
Underlying traits	Birth weight*	289,846	-
	Gestational age*	189,471	-

* continuous traits

Details on trait definitions can be found in Supplementary Tables 2A and 2B

For peer review only - http://bmjopen.bmj.com/site/about/guidelines.xhtml

Table 2. Maternal participant characteristics in the participating core studies#

Characteristics	ALSPAC	BiB	MoBa	UK Biobank
Total N	13,895	9,407	93,512	224,423
Age, mean (SD)	28.0 (5.0)	27.4 (5.6)	30.1 (4.7)	25.3 (4.6)*
Missing	369 (2.7%)	1,169 (12.4%)	458 (0.5%)	45,402 (20.2%)
Ethnicity (White), N (%)	11,600 (97.4%)	3,430 (36.4%)	88,989 (95.1%)	211,677 (94.6%)
Missing	1,982 (14.3%)	1,025 (10.1%)	2,021 (2.1%)	706 (0.3%)
Education (university level), N (%)	1,544 (12.9%)	1,432 (11.8%)	50,047 (53.5%)	65,334 (29.5%)**
Missing	1,890 (13.6%)	1,186 (12.6%)	11,064 (11.8%)	2,714 (1.2%)
Index of multiple deprivation, (N%)				
1st quintile (most deprived)	1,628 (13.9%)	5,232 (55.6%)	NA	NA
5th quintile (least deprived)	3,045 (26.0%)	158 (1.7%)	NA	NA
Missing	2,197 (15.8%)	1,171 (12.4%)		
Parity (nulliparous), N(%)	5,619 (44. 7%)	3,768 (40.0%)	42,135 (45.1%)	NA¥
Missing	1,333 (9.6%)	345 (3.6%)	399 (0.4%)	
Smoking during pregnancy (ever), N (%)	3,301 (25.9%)	1,408 (14.9%)	5,939 (6.4%)	NA
Missing	1,124 (8.1%)	1,185 (12.6%)	16,824 (18.0%)	
Alcohol intake during pregnancy, N (%)	8,036 (64.0%)	1,707 (18.1%)	2,472 (2.6%)	NA
Missing	1,340 (9.6%)	2,114 (22.9%)	27,648 (29.6%)	
Body mass index§, mean (SD)	22.9 (3.8)	26.1 (5.7)	24.1 (4.3)	NA**
Missing	2,711 (19.5%)	1,562 (16.6%)	11,561 (12.3%)	

Participants included if they had data on at least one APPO
* age at first birth; ** information assessed at recruitment and not at pregnancy; ¥ In UKB, the sample was restricted to women who have ever been pregnant; § Assessed pre- or during pregnancy

For peer review only - http://bmjopen.bmj.com/site/about/guidelines.xhtml

Abbreviations: ALSPAC, The Avon Longitudinal Study of Parents and Children; BiB, Born in Bradford; MoBa, The Norwegian Mother, Father and Child cohort study; SD, standard deviation; NA, information not available in this study. This includes all available data at the outcome level.

For peer review only

For peer review only - http://bmjopen.bmj.com/site/about/guidelines.xhtml

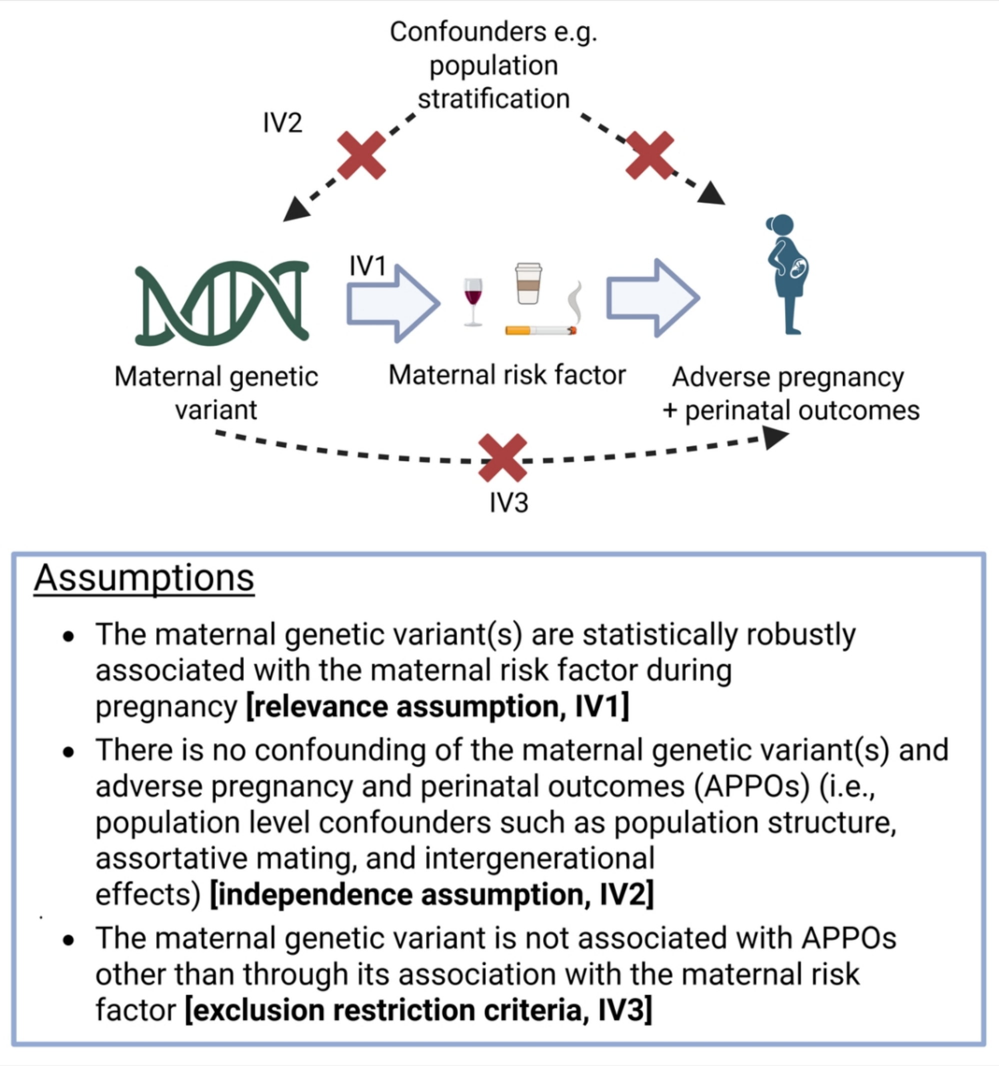

Figure 1 Summary of the core Mendelian randomizsation assumptions to estimate the presence of an effect of maternal risk factors on adverse pregnancy and perinatal outcomes

102x109mm (300 x 300 DPI)

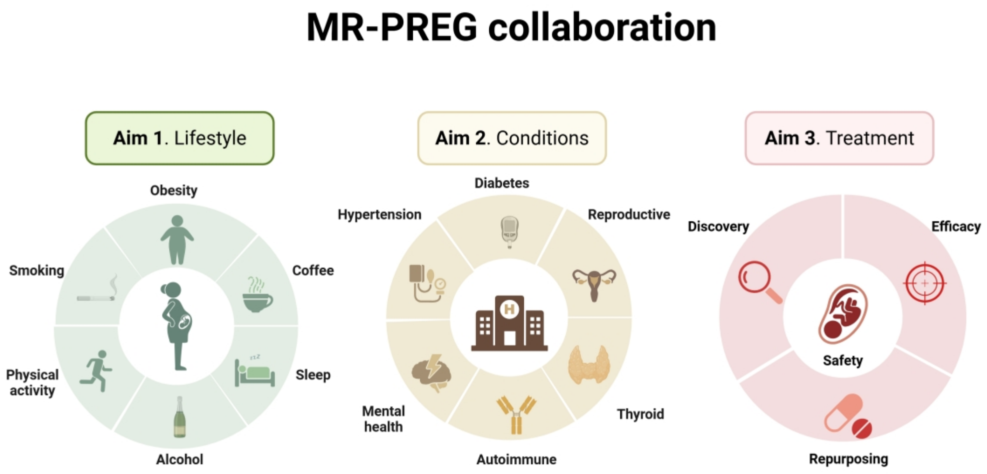

Figure 2 MR-PREG collaboration aims

254x129mm (300 x 300 DPI)

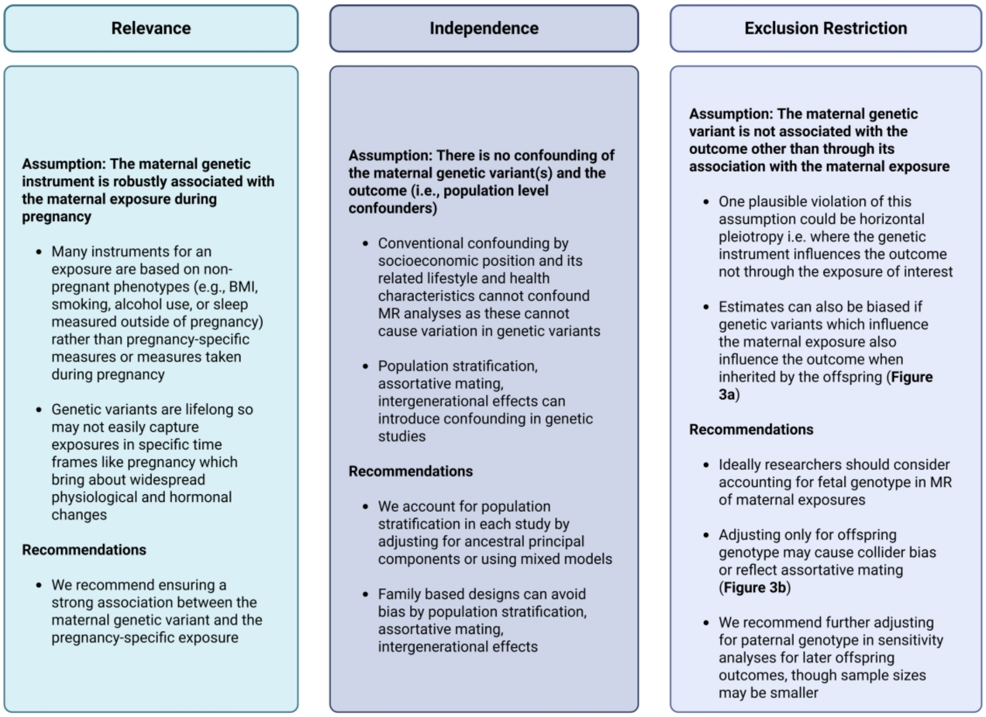

Box 1 Approaches to explore the plausibility of the Mendelian randomisation core assumptions (Figure 1), with a focus on the context of maternal risk factors and adverse pregnancy and perinatal outcomes (APPOs). For a review of standard MR sensitivity analyses, we refer readers to Sanderson et al.

287x207mm (300 x 300 DPI)

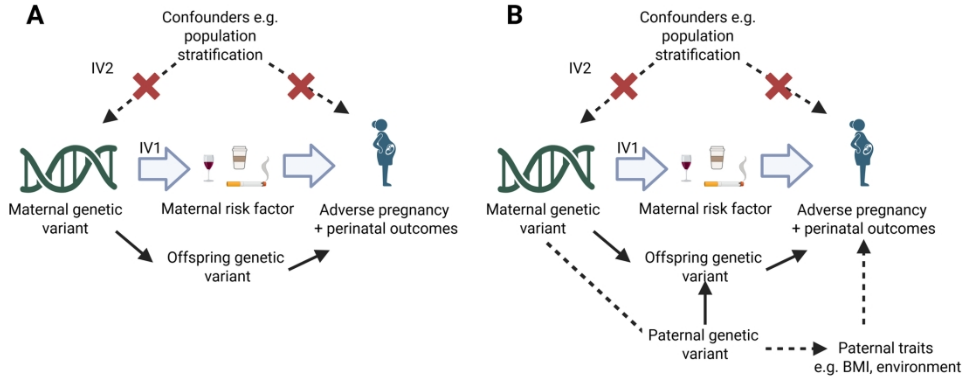

Figure 3a Illustration of how the exclusion restriction assumption may be violated; Figure 3b Illustration of how collider bias might be induced in MR analysis when conditioning on offspring genetic variants. Offspring genetic variation might violate the exclusion restriction assumption (Figure 3a). Adjusting for offspring genetic variants only (which are a collider variables) (Figure 3b) means that a false association between maternal and paternal genetic variants can be created (shown as a dashed line). Such associations can also arise via assortative mating. This will only cause bias if the outcome is influenced by paternal genetic variants, independently of maternal and offspring genetic variants. However, by additionally adjusting for paternal genetic variants, the pathway highlighted in black dashed arrows can be closed.

184x75mm (300 x 300 DPI)

The Mendelian Randomization in Pregnancy (MR-PREG) collaboration: Improving evidence for prevention and treatment of adverse pregnancy and perinatal outcomes

Contents

Genotyping, imputation and quality control

Genome-wide association analyses

Weighted linear model (WLM)

Ethical approval

Acknowledgments

Funding

For peer review only

For peer review only - http://bmjopen.bmj.com/site/about/guidelines.xhtml

Genotyping, imputation and quality control

Genotyping, imputation and quality control (QC) procedures were applied within each cohort and are described below.

Avon Longitudinal Study of Parents and Children (ALSPAC)

Avon Longitudinal Study of Parents and Children (ALSPAC) children [1] were genotyped using the Illumina HumanHap550 quad chip genotyping platforms by 23andMe subcontracting the Wellcome Trust Sanger Institute, Cambridge, UK and the Laboratory Corporation of America, Burlington, NC, US. The resulting raw genome-wide data were subjected to standard QC methods. Individuals were excluded based on gender mismatches; minimal or excessive heterozygosity; disproportionate levels of individual missingness (> 3%) and insufficient sample replication (identity by descent [IBD] < 0.8). Population stratification was assessed by multidimensional scaling analysis and compared with Hapmap II (release 22) European descent (CEU), Han Chinese, Japanese and Yoruba reference populations; all individuals with non-European ancestry were removed. Single nucleotide polymorphisms (SNPs) with a minor allele frequency (MAF) of < 1%, a call rate of < 95% or evidence for violations of Hardy-Weinberg equilibrium (HWE, P < 5e-7) were removed. Cryptic relatedness was measured as proportion of IBD > 0.1. Related subjects that passed all other QC thresholds were retained during subsequent phasing and imputation. 9,115 subjects and 500,527 SNPs passed these QC filters.

ALSPAC mothers [2] were genotyped using the Illumina human660W-quad array at Centre National de Génotypage (CNG) and genotypes were called with Illumina GenomeStudio. PLINK (v1.07) was used to carry out QC measures on an initial set of 10,015 subjects and 557,124 directly genotyped SNPs. SNPs were removed if they displayed more than 5% missingness or a HWE p-value of less than 1e-6. Additionally, SNPs with MAF < 1% were removed. Samples were excluded if they displayed more than 5% missingness, had indeterminate X chromosome heterozygosity or extreme autosomal heterozygosity. Samples showing evidence of population stratification were identified by multidimensional scaling of genome-wide identity by state pairwise distances using the four HapMap populations as a reference and then excluded. Cryptic relatedness was assessed using an IBD estimate of more than 0.125, which is expected to correspond to 12.5% alleles

For peer review only - http://bmjopen.bmj.com/site/about/guidelines.xhtml

shared IBD or relatedness at the first cousin level. Related subjects that passed all other QC thresholds were retained during subsequent phasing and imputation. 9,048 subjects and 526,688 SNPs passed these QC filters.

SNP genotypes (477,482 SNPs) in common between the sample of mothers and sample of children were combined. SNPs with genotype missingness above 1% (N = 11,396 SNPs) or individuals with potential ID mismatches (N = 321 individuals) were removed. This resulted in a dataset of 17,842 subjects containing 6,305 duos and 465,740 SNPs (112 were removed during liftover and 234 were out of HWE after combination).

Haplotypes were estimated using ShapeIT (v2.r644) which utilises relatedness during phasing. The phased haplotypes were then imputed to the Haplotype Reference Consortium (HRC) r1.1, 2016 panel of approximately 31,000 phased whole genomes. The HRC panel was phased using ShapeIt v2, and the imputation was performed using the Michigan imputation server. This resulted in 8,237 eligible children and 8,196 eligible mothers with available genotype data after exclusion of related subjects using the cryptic relatedness measures described previously. Reference genome build version 37 (GRCh37/hg19) was used for genomic positions for ALSPAC genotype data.

Born in Bradford (BiB)

Born in Bradford (BiB) [3] mothers and offspring were genotyped at Bristol Bioresource Laboratories, Bristol, UK, using four different Illumina arrays: HumanCoreExome12v1.0, HumanCoreExome12v1.1, HumanCoreExome24v1.0 and Infinium Global Screening Array-24 v1.0 (GSA). Genotypes were called with Illumina GenomeStudio. For each of the four subsets of participants genotyped on each array, any individual or SNP with > 3% missing genotype calls was dropped, and the four subsets were merged, giving a total merged sample of 20,000 individuals. It was not appropriate to apply filters for deviation from HWE and excess homozygosity to this merged dataset, given the population structure and consanguinity known to be present. 707 individuals were then dropped due to i) mismatch between genetically- and phenotypically-defined sex (110 individuals), ii) suspected sample duplication (127 individuals), and iii) discrepancy between expected and genetically inferred relationship for first degree relatives (470

For peer review only - http://bmjopen.bmj.com/site/about/guidelines.xhtml

individuals). Principal component analysis (PCA) was carried out and involved assigning participants to two groups that were genetically similar to the European or South Asian HapMap samples. Ancestry data were also self-reported or obtained from primary care medical records, and categorised using the UK Office of National Statistics criteria: (1) White European ('White British' or 'White European'), (2) South Asian ('Pakistani', 'Indian' or 'Bangladeshi'), (3) Caribbean or African ('Afro-Caribbean' or 'African') or (4) others. Individuals whose PCA-based ancestry agreed with their self-reported/medical record-derived ancestry were assigned to two groups, for which we use the shorthand "White European" (WE) and "South Asian" (SA). The SA group consisted primarily of individuals with Pakistani self-reported/medical record ancestry and does not include Bangladeshi ancestry individuals but does include some Indian ancestry individuals.

Genotype imputation was performed for 7606 WE and 8692 SA participants, with each ancestry and array (CoreExome/GSA) imputed separately. Prior to imputation, variants with call rate < 95%, HWE p-value < 1e-6 or MAF < 1% were removed for each of the four ancestry/array groups separately. A/T and C/G SNPs were dropped, as were indels, non-autosomal SNPs, SNPs with no match in the HRC panel (position or SNP ID), SNPs with non-matching alleles, and those that failed checks for strand, reference/alternative alleles, position, and SNP duplication. Genotype data were imputed to the HRC r1.1 reference panel. Reference genome build version 37 (GRCh37/hg19) was used for genomic positions for BiB genotype data.

The Norwegian Mother Father and Child Cohort Study (MoBa)

In The Norwegian Mother Father and Child Cohort Study (MoBa), [4] genotyping of the samples was performed in 24 different batches on different Illumina platforms (HumanCoreExome-12 v.1.1, HumanCoreExome-24 v.1.0, Global Screening Array MD v.1.0, Global Screening Array MD v.3.0, HumanOmniExpress-24-v1.0 and InfiniumOmniExpress-24v1.2) at deCODE Genetics, Reykjavik, Iceland, the Genomics Core Facility, Trondheim, Norway, and Erasmus MC, Rotterdam, Netherlands. Further details of the procedures used for genotyping, genotype imputation and quality control of the genetic data in MoBa are described elsewhere. [5] In total, 238,001 MoBa samples were sent for genotyping. We used reference genome build version 37 (GRCh37/hg19) for genomic positions for MoBa genotype data.

For peer review only - http://bmjopen.bmj.com/site/about/guidelines.xhtml

Subpopulations were defined via PCA using 1000 Genomes phase 1 data to identify the European, Asian, and African core subpopulations (after temporarily removing SNPs with MAF < 1%, call rate < 95%, and HWE p-value < 0.001 and individuals with call rate ≤ 95%). A comprehensive suite of pre-imputation QC measures was then performed for each subpopulation on the SNP, individual, and family level. [5] The primary software packages used for the QC procedures were PLINK 1.9 and KING 2.2.5. The SNP-level QC included removing SNPs if MAF < 0.5%, call rate < 98%, HWE p-value < 1e-6, discordant in duplicate pairs, associated with genotype plate and genotype batch at p-value 0.001. The individual-level QC included removing individuals if they were heterozygosity outliers (± 3 standard deviations from the mean heterozygosity across all individuals), had erroneous sex assignment, were duplicate samples, had implausible or erroneous relationships (reported by the participants or inferred via the KING method) had excessive cryptic relatedness, had unexpectedly high identity-by-decent (PI_HAT > 0.15), or were principal component (PC) outliers. The family-level QC included removing families with Mendelian errors at > 5% of SNPs. Genotyping batches that were genotyped using the same array were merged and the pre-imputation QC was performed on the merged batches, keeping only SNPs that passed three rounds of subpopulation QC in all the batches genotyped on the same array.

Phasing and imputation were performed using the publicly available HRC data. Prior to phasing, further QC filters were applied, including dropping strand ambiguous (A/T and C/G) SNPs and indels. [5] Phasing was performed using SHAPEIT2 release 904 with the duoHMM algorithm to incorporate the pedigree information into the haplotype estimates. IMPUTE4.1.2_r300 was then used to perform imputation. Dosage data were converted to best-guess (hard call) genotype data using certainty threshold 0.7, and variants with imputation quality score (INFO) ≤ 0.8 were dropped.

Post-imputation QC was then performed as for the steps outlined for pre-imputation QC, with the following changes: i) MAF filter 1%, ii) call rate < 95% for SNPs, and iii) p-value for batch effects filter < 5e-8. To ensure that across batch relatedness (both known and unknown) was accounted for in all analyses the three imputation batches were then merged, and the post-

For peer review only - http://bmjopen.bmj.com/site/about/guidelines.xhtml

imputation QC steps were repeated, with the exception that a filter was applied for imputation batch effects rather than genotype batch effects.

UK Biobank

A full description of the genetic QC performed by the MRC-Integrative Epidemiology Unit on UK Biobank data is available elsewhere. [6] The full data release contains the cohort of successfully genotyped samples (n=488,377). 49,979 individuals were genotyped using the UK BiLEVE array and 438,398 using the UK Biobank axiom array. Pre-imputation QC, phasing and imputation are described elsewhere. [7] In brief, prior to phasing, multiallelic SNPs or those with MAF ≤ 1% were removed. Phasing of genotype data was performed using a modified version of the SHAPEIT2 algorithm. [8] Genotype imputation to a reference set combining the UK10K haplotype and HRC reference panels [9] was performed using IMPUTE2 algorithms. [10] The analyses presented here were restricted to autosomal variants using a graded filtering with varying imputation quality for different allele frequency ranges. Therefore, rarer genetic variants are required to have a higher imputation INFO score (INFO > 0.3 for MAF > 3%; INFO > 0.6 for MAF 1-3%; INFO > 0.8 for MAF 0.5-1%; INFO > 0.9 for MAF 0.1-0.5%) with MAF and Info scores having been recalculated on an in-house derived 'European' subset.

In post-imputation QC, individuals with sex-mismatch (derived by comparing genetic sex and reported sex) or individuals with sex chromosome aneuploidy were excluded from the analysis (n=814). The sample was restricted to individuals of 'European' ancestry as defined by an in-house k-means cluster analysis performed using the first 4 PCs provided by UK Biobank in the statistical software environment R. The current analysis includes the largest cluster from this analysis (n=464,708). Estimated kinship coefficients using the KING toolset [11] identified 107,162 pairs of related individuals. An in-house algorithm was then applied to this list and preferentially removed the individuals related to the greatest number of other individuals until no related pairs remain. These individuals were excluded (n=79,448). Additionally, 2 individuals were removed due to them relating to a very large number (> 200) of individuals. Reference genome build version 37 (GRCh37/hg19) was used for genomic positions for UKB genotype data.

For peer review only - http://bmjopen.bmj.com/site/about/guidelines.xhtml

FinnGen

A full description of the FinnGen study and the QC procedures applied to the genetic data is available elsewhere. [12,13] We used release 12 of the publicly available FinnGen summary statistics. FinnGen individuals were genotyped with Illumina and Affymetrix chip arrays (Illumina Inc., San Diego, and Thermo Fisher Scientific, Santa Clara, CA, USA). Genotype calls were made with the GenCall and the zCall algorithms for Illumina and AxiomGT1 algorithm for Affymetrix data. Chip genotyping data produced with previous chip platforms and reference genome builds were lifted over to build version 38 (GRCh38/hg38). In sample-wise QC steps, individuals with ambiguous gender, high genotype missingness (> 5%), excess heterozygosity (+-4SD) and non-Finnish ancestry were excluded. In variant-wise QC steps, variants with high missingness (> 2%), low HWE p-value (< 1e-6) and low minor allele count (MAC < 3) were excluded. Before imputation, chip-genotyped samples were pre-phased with Eagle 2.3.5 using the default parameters, except the number of conditioning haplotypes, which was set to 20,000. Chip genotype data were imputed using the population-specific SISu v4.2 imputation reference panel of 8,554 whole genomes with Beagle 4.1 (version 27Jan18.7e1). Post-imputation QC involved checking the expected conformity of the imputation INFO-value distribution, MAF differences between the target dataset and the imputation reference panel and checking chromosomal continuity of the imputed genotype calls. Merged imputed genotype data is composed of 141 data sets and includes samples from multiple cohorts, yielding a total sample size of 520,210 participants with genotype data for 21,311,644 variants.

The FinnGen data for 180,042 pruned SNPs was merged with the 1k genome project (1kgp) data, and two rounds of PCA were carried out. After kinship analysis, the sample was separated into three groups: 296,829 inliers (unrelated participants with Finnish ancestry), 203,908 outliers (non-duplicate samples with Finnish ancestry but who are also related to the inliers), and 19,473 rejected participants who had non-Finnish ancestry, or who were twins/duplicates with relations to other (non-inlier) samples. A final round of PCA for the inliers was calculated, after which outliers were projected onto the same PC space, yielding PC covariates for a total of 500,737 participants. After dropping 355 participants due to missing minimum phenotype data, and dropping 34 participants due to sex check failure, 500,348 participants were available for

For peer review only - http://bmjopen.bmj.com/site/about/guidelines.xhtml

genome-wide association study (GWAS) analyses, of which 282,064 were female. Reference genome build version 38 (GRCh38/hg38) was used for genomic positions for FinnGen.

Genome-wide association analyses

GWAS analyses were run separately for mothers, fathers and offspring (when sufficient data were available) within each cohort, using software and approaches which were tailored to optimally account for the population stratification and relatedness present in each sample.

ALSPAC

We performed GWAS analysis in ALSPAC mothers and offspring using PLINK 2.0 alpha (build 22 Dec 2024) [14 15]. The analytical samples were restricted to unrelated mothers (IBD ≤ 0.125) and offspring (IBD ≤ 0.1). Association analyses were performed with imputed autosomal genotype data for adverse pregnancy and perinatal outcomes (APPOs) via the "--glm" command using Firth logistic regression for binary outcomes and linear regression for continuous outcomes. Firth regression enables less biased effect size estimation in the presence of case-control imbalance for binary outcomes.[16] We assumed an additive model and adjusted for the top 10 PCs of ancestry. PCs were generated using PLINK –pca option after removing related individuals based on a set of independent SNPs excluding any long-range linkage disequilibrium (LD) regions (variant count window size = 100, variant count to shift the window = 5, variance inflation factor (VIF) threshold = 1.01).

BiB

We performed GWAS analysis in BiB mothers and offspring using REGENIE version 3.6 [17] to account for the complex population structure (population stratification and relatedness) that is present in BiB, via a whole-genome regression approach. REGENIE implements whole-genome regression in two steps: in step 1, array genotypes are used to generate 22 leave-one-chromosome-out (LOCO) predictions for each outcome. In step 2, during which the imputed genotype associations with each outcome are estimated, these LOCO predictions are then used as covariates. We performed GWAS separately for the two largest ancestry groups present in BiB

For peer review only - http://bmjopen.bmj.com/site/about/guidelines.xhtml

as described above (SA and WE). We did not use REGENIE's automatic phenotype imputation feature for missing APPOs and instead ran REGENIE for each outcome separately. For step 1 we used the called genotype data obtained by merging the samples genotyped on different arrays (as described above), after which the PLINK 2.0 filters "--maf 0.01", "--hwe 0.000001" and "--geno 0.03" were applied, yielding sets of 99,131 and 135,801 variants for the SA and WE samples, respectively. For step 1 we set genotype block size ("--bsize") to 1000. For step 2, we ran association analyses with imputed autosomal genotype data, having first merged the samples genotyped on the CoreExome and GSA arrays and subset variants to those with imputation accuracy $R^2 \geq 0.3$ when imputed from both arrays. We set genotype block size to 200, applied a minimum MAC filter of 20, and used linear regression or Firth logistic regression (via REGENIE options "--firth --approx --pThresh 0.999999") for continuous and binary outcomes, respectively. We computed the standard error based on the Firth regression effect size and likelihood ratio test *P*-value as per the "--firth-se" option. Models were adjusted for genotyping array and 40 PCs: 20 internal PCs (calculated using only BiB data via the flashpca package version 2.0 [18] with the authors' recommended settings [18 19]) and 20 external PCs (BiB participants were projected onto a UK Biobank PC space via the pcapred R package. [20]

MoBa

We performed GWAS analysis in MoBa mothers, fathers and offspring using whole genome regression implemented in REGENIE version 3.1.2. [17] We did not use REGENIE's automatic phenotype imputation feature for missing APPOs and instead ran REGENIE for each outcome separately. We performed step 1 using 455,827 imputed genetic variants which were directly genotyped in ≥ 1 imputation batch and had imputation quality INFO score > 0.99785, having first removed 35,810 variants in the major histocompatibility complex (MHC) region on chromosome 6 (variants with MAF < 1% had already been removed in pre-phase QC). We set genotype block size ("--bsize") to 1000. For step 2, we ran association analyses with imputed (hard called) autosomal genotype data for 6,981,748 SNPs, adjusting for genotyping batch and 20 PCs. We set genotype block size to 200 and used linear regression or Firth logistic regression (via REGENIE options "--firth --approx --pThresh 0.999999") for continuous and binary outcomes, respectively.

For peer review only - http://bmjopen.bmj.com/site/about/guidelines.xhtml

We computed the standard error based on the Firth regression effect size and likelihood ratio test *P*-value as per the "--firth-se" option.

UK BioBank

We performed GWAS analysis in UK Biobank mothers only, using whole genome regression implemented in REGENIE version v3.6. [17] We did not use REGENIE's automatic phenotype imputation feature for missing APPOs and instead ran REGENIE for each outcome separately. We performed step 1 using 549,043 directly genotyped variants with MAF ≥ 0.01, variant call rate ≥ 0.9; HWE $P > 1.0 \times 10^{-15}$ and MAC ≥ 100, and set genotype block size ("--bsize") to 1000. For step 2, we ran association analyses with imputed genotype data for the APPOs, adjusting for genotyping array and 40 PCs. We set genotype block size to 200 and used linear regression or Firth logistic regression (via REGENIE options "--firth --approx --pThresh 0.999999") for continuous and binary outcomes, respectively. We computed the standard error based on the Firth regression effect size and likelihood ratio test *P*-value as per the "--firth-se" option.

FinnGen

GWAS analyses were caried out in FinnGen mothers only, using whole genome regression implemented in REGENIE version 2.2.4. [17] For REGENIE step 1, 215,152 pruned (1.5Mb window and r2 threshold of 0.2) variants were used after applying the following filters: i) imputation INFO score > 0.95 in all batches, ii) > 97 % non-missing genotype, and iii) MAF > 1 %, and a genotype block size of 1000 was used. For REGENIE step 2, association analyses with imputed genotype data for APPOs were conducted for each variant with a minimum allele count of 5 among each phenotype's cases and controls. The approximate Firth test was used for variants with an initial p-value of less than 0.01 and the standard error was computed based on the Firth regression effect size and likelihood ratio test *P*-value (REGENIE options --firth --approx --pThresh 0.01 --firth-se).

For peer review only - http://bmjopen.bmj.com/site/about/guidelines.xhtml

Post-GWAS quality control

Prior to meta-analysis we excluded variants with low MAF (< 0.01) or low INFO score (< 0.4) from the summary statistics from individual cohorts and maternal/paternal/offspring samples. We applied a standardised QC pipeline to GWAS summary statistics from individual cohorts and from the meta-analysis using the GWASInspector R package. [21] This QC process included comparing the allele frequencies (AF) in the GWAS summary statistics to those in suitable reference panels. In the FinnGen summary statistics a small subset of variants had the opposite AF to the HRC reference panel (i.e. $AF_{FinnGen} = 1 - AF_{HRC}$). We therefore dropped 4064 variants which had a FinnGen versus HRC AF difference > 0.2 from the meta-analyses.

Weighted linear model (WLM)

We carried out marginal (unadjusted) GWAS as described above, in which outcomes were regressed separately on maternal and offspring genotype, without mutual adjustment of the effects of maternal and offspring genotypes. After meta-analysis of the marginal GWAS effects we applied a weighted linear model (WLM) [22-24] implemented in the DONUTS R package [25] to estimate conditional (adjusted) maternal ($\hat{\beta}_m$) and offspring ($\hat{\beta}_o$) genetic effects (the mutually adjusted coefficients for maternal and offspring genotype, fitted jointly in the same model), as linear combinations of the estimated marginal offspring ($\hat{b}_o$) and maternal ($\hat{b}_m$) genetic effects:

$$\hat{\beta}_o = \frac{4}{3}\hat{b}_o - \frac{2}{3}\hat{b}_m$$

$$\hat{\beta}_m = \frac{4}{3}\hat{b}_m - \frac{2}{3}\hat{b}_o$$

$$\mathrm{SE}(\hat{\beta}_o) = \sqrt{\frac{16}{9}\mathrm{var}(\hat{b}_o) + \frac{4}{9}\mathrm{var}(\hat{b}_m) - \frac{16}{9} \times \widehat{\mathrm{int}}_{o,m} \times \mathrm{SE}(\hat{b}_m)\mathrm{SE}(\hat{b}_o)}$$

$$\mathrm{SE}(\hat{\beta}_m) = \sqrt{\frac{16}{9}\mathrm{var}(\hat{b}_m) + \frac{4}{9}\mathrm{var}(\hat{b}_o) - \frac{16}{9} \times \widehat{\mathrm{int}}_{o,m} \times \mathrm{SE}(\hat{b}_m)\mathrm{SE}(\hat{b}_o)}$$

where $\widehat{\mathrm{int}}_{o,m}$ is the intercept term from bivariate LD score regression [26] of the maternal and offspring marginal summary statistics. These intercept terms are necessary to account for sample overlap between the marginal GWAS samples, and were estimated using Hapmap 3 SNPs with

For peer review only - http://bmjopen.bmj.com/site/about/guidelines.xhtml

MAF > 1%, excluding SNPs in the MHC region and with association Chi square statistics > 80. We refer to the above model as the duos WLM, and it assumes that conditional paternal effects are zero.

Paternal genotype data were available in MoBa. We therefore also conducted marginal paternal GWAS, enabling us to relax the assumption that conditional paternal effects are zero by estimating the conditional maternal, offspring and paternal ($\hat{\beta}_p$) genetic effects via a trios WLM:

$$\hat{\beta}_o = 2\hat{b}_o - \hat{b}_m - \hat{b}_p$$

$$\hat{\beta}_m = \frac{3}{2}\hat{b}_m - \hat{b}_o + \frac{1}{2}\hat{b}_p$$

$$\hat{\beta}_p = \frac{3}{2}\hat{b}_p - \hat{b}_o + \frac{1}{2}\hat{b}_m$$

with their standard errors:

$$\mathrm{SE}(\hat{\beta}_o) = \sqrt{4\mathrm{var}(\hat{b}_o) + \mathrm{var}(\hat{b}_m) + \mathrm{var}(\hat{b}_p) + 2 \times \mathrm{int}_{m,p} \times \mathrm{SE}(\hat{b}_m)\mathrm{SE}(\hat{b}_p) - 4 \times \mathrm{int}_{o,m} \times \mathrm{SE}(\hat{b}_o)\mathrm{SE}(\hat{b}_m) - 4 \times \mathrm{int}_{o,p} \times \mathrm{SE}(\hat{b}_o)\mathrm{SE}(\hat{b}_p)}$$

$$\mathrm{SE}(\hat{\beta}_m) = \sqrt{\frac{9}{4}\mathrm{var}(\hat{b}_m) + \mathrm{var}(\hat{b}_o) + \frac{1}{4}\mathrm{var}(\hat{b}_p) - \mathrm{int}_{o,p} \times \mathrm{SE}(\hat{b}_o)\mathrm{SE}(\hat{b}_p) - 3 \times \mathrm{int}_{o,m} \times \mathrm{SE}(\hat{b}_o)\mathrm{SE}(\hat{b}_m) + \frac{3}{2} \times \mathrm{int}_{m,p} \times \mathrm{SE}(\hat{b}_m)\mathrm{SE}(\hat{b}_p)}$$

$$\mathrm{SE}(\hat{\beta}_p) = \sqrt{\frac{9}{4}\mathrm{var}(\hat{b}_p) + \mathrm{var}(\hat{b}_o) + \frac{1}{4}\mathrm{var}(\hat{b}_m) - \mathrm{int}_{o,m} \times \mathrm{SE}(\hat{b}_o)\mathrm{SE}(\hat{b}_m) - 3 \times \mathrm{int}_{o,p} \times \mathrm{SE}(\hat{b}_o)\mathrm{SE}(\hat{b}_p) + \frac{3}{2} \times \mathrm{int}_{m,p} \times \mathrm{SE}(\hat{b}_m)\mathrm{SE}(\hat{b}_p)},$$

where $\mathrm{int}_{o,m}$, $\mathrm{int}_{o,p}$, and $\mathrm{int}_{m,p}$ are the intercepts from bivariate LD score regression of the maternal and offspring, paternal and offspring and maternal and paternal marginal summary statistics respectively, and $\hat{b}_p$ is the estimated marginal paternal genetic effect.

The DONUTS software package extends the duos and trios WLMs to account for assortative mating via a parameter α. We set α to zero (i.e. we assumed mating was random) because previous sensitivity analyses in MoBa suggested this gave the most accurate estimates for conditional effects on birth weight. [27]

For peer review only

Ethical approval

ALSPAC

Ethical approval for the study was obtained from the ALSPAC Ethics and Law Committee and the Local Research Ethics Committees. Consent for biological samples has been collected in accordance with the Human Tissue Act (2004).

Ethical approval was obtained from the ALSPAC Ethics and Law Committee and the Local Research Ethics Committees. Consent for biological samples has been collected in accordance with the Human Tissue Act (2004). Informed consent for the use of data collected via questionnaires and clinics was obtained from participants following the recommendations of the ALSPAC Ethics and Law Committee at the time (details and reference numbers of all ethics approvals can be found at http://www.bristol.ac.uk/media-library/sites/alspac/documents/governance/Research%20Ethics%20Committee%20approval%20references.pdf).

BiB

Ethical approval for the study was granted by the Bradford National Health Service Research Ethics Committee (ref 06/Q1202/48), and all participants gave written informed consent. The ALL IN sub-study had ethical approval from the London School of Hygiene & Tropical Medicine ethics committee (ref: 5320) and the Bradford Research Ethics committee (ref: 08/H1302/21). Parents (usually the mother) gave informed, written consent to take part in the study.

MoBa

The current study is based on version 12 of the quality-assured data files released for research in 2019. The establishment of MoBa and initial data collection was based on a license from the Norwegian Data Protection Agency and approval from The Regional Committees for Medical and Health Research Ethics. The MoBa cohort is currently regulated by the Norwegian Health Registry Act. The current study was approved by The Regional Committees for Medical and Health Research Ethics of South/East Norway (ref 2018/1256).

For peer review only - http://bmjopen.bmj.com/site/about/guidelines.xhtml

UK Biobank

The UK Biobank has approval from the North West Multi-centre Research Ethics Committee (MREC) as a Research Tissue Bank (RTB) approval. This RTB approval was granted initially in 2011 (11/NW/0382) and it is renewed on a 5-yearly cycle, with the latest one successfully renewed in 2021 (21/NW/0157).

Acknowledgments

ALSPAC

We are extremely grateful to all the families who took part in this study, the midwives for their help in recruiting them, and the whole ALSPAC team, which includes interviewers, computer and laboratory technicians, clerical workers, research scientists, volunteers, managers, receptionists and nurses.

BiB

BiB is only possible because of the enthusiasm and commitment of the Children and Parents in BiB. We are grateful to all the participants, teachers, school staff, health professionals and researchers who have made BiB happen.

MoBa

This research has been conducted using MoBa data using application number 2552. MoBa is supported by the Norwegian Ministry of Health and Care services and the Ministry of Education and Research. We are grateful to all the participating families in Norway who take part in this on-going cohort study. We thank the Norwegian Institute of Public Health (NIPH) for generating high-quality genomic data. This research is part of the HARVEST collaboration, supported by the Research Council of Norway (#229624). We also thank the NORMENT Centre for providing genotype data, funded by the Research Council of Norway (#223273), South East Norway Health Authority and KG Jebsen Stiftelsen. We further thank the Center for Diabetes Research, the University of Bergen for providing genotype data and performing QC and imputation of the data funded by the ERC AdG project SELECTionPREDISPOSED, Stiftelsen Kristian Gerhard Jebsen, Trond

For peer review only - http://bmjopen.bmj.com/site/about/guidelines.xhtml

Mohn Foundation, the Research Council of Norway, the Novo Nordisk Foundation, the University of Bergen, and the Western Norway health Authorities (Helse Vest).

FinnGen

The authors thank the FinnGen investigators for sharing their summary-level data.

UK Biobank

We would like to thank all the participants of UK Biobank for their vital contribution to the resource. This research has been conducted using the UK Biobank Resource under Application Number 23938**.**

Funding

ALSPAC

The UK Medical Research Council and Wellcome (Grant ref: 217065/Z/19/Z) and the University of Bristol provide core support for ALSPAC.A comprehensive list of grants funding is available on the ALSPAC website (http://www.bristol.ac.uk/alspac/external/documents/grant-acknowledgements.pdf). ALSPAC GWAS data was generated by Sample Logistics and Genotyping Facilities at Wellcome Sanger Institute and LabCorp (Laboratory Corporation of America) using support from 23andMe. This research was funded in part by the Wellcome Trust (Grant ref: 217065/Z/19/Z)]. For the purpose of Open Access, the author has applied a CC BY public copyright licence to any Author Accepted Manuscript version arising from this submission.

MoBa

MoBa funding is under **Acknowledgements** as requested by MoBa publication guidelines.

BiB

BiB receives core funding from the Wellcome Trust (WT101597MA), a joint grant from the UK Medical and Economic and Social Science Research Councils (MR/N024397/1), British Heart Foundation (CS/16/4/32482), and the National Institute of Health Research under its Applied

For peer review only - http://bmjopen.bmj.com/site/about/guidelines.xhtml

Research Collaboration for Yorkshire and Humber and Clinical Research Network research delivery support. Further support for genome-wide and multiple 'omics measurements in BiB is from the UK Medical Research Council (G0600705), National Institute of Health Research (NF-SI-0611-10196), US National Institute of Health (R01DK10324), and the European Research Council under the European Union's Seventh Framework Programme (FP7/2007–2013) / ERC grant agreement no 669545.

UK Biobank

UK Biobank is funded primarily by the Wellcome Trust and the Medical Research Council (MRC). It is also funded by the Department of Health, British Heart Foundation, Cancer Research UK, Diabetes UK, National Institute for Health Research (NIHR), Scottish Government, Northwest Regional Development Agency, and Welsh Assembly Government.

For peer review only

For peer review only - http://bmjopen.bmj.com/site/about/guidelines.xhtml

References

1. Boyd A, Golding J, Macleod J, et al. Cohort profile: the 'children of the 90s'—the index offspring of the Avon Longitudinal Study of Parents and Children. *International journal of epidemiology* 2013;42(1):111-27.
2. Fraser A, Macdonald-Wallis C, Tilling K, et al. Cohort profile: The Avon Longitudinal Study of Parents and Children: ALSPAC mothers cohort. *International Journal of Epidemiology* 2013;42 doi: 10.1093/ije/dys066
3. Wright J, on behalf of the Born in Bradford Scientific Collaborators G, Small N, et al. Cohort Profile: The Born in Bradford multi-ethnic family cohort study. *International Journal of Epidemiology* 2013;42(4):978-91. doi: 10.1093/ije/dys112
4. Corfield EC, Frei O, Shadrin AA, et al. The Norwegian Mother, Father, and Child cohort study (MoBa) genotyping data resource: MoBaPsychGen pipeline v.1. *bioRxiv* 2022:2022.06.23.496289. doi: 10.1101/2022.06.23.496289
5. Corfield EC, Shadrin AA, Frei O, et al. The Norwegian Mother, Father, and Child cohort study (MoBa) genotyping data resource: MoBaPsychGen pipeline v.1. *bioRxiv* 2024:2022.06.23.496289. doi: 10.1101/2022.06.23.496289
6. Mitchell R, Hemani G, Dudding T, et al. UK Biobank Genetic Data: MRC-IEU Quality Control, version 2, 2019.
7. Bycroft C, Freeman C, Petkova D, et al. The UK Biobank resource with deep phenotyping and genomic data. *Nature* 2018;562(7726):203-09. doi: 10.1038/s41586-018-0579-z
8. O'Connell J, Sharp K, Shrine N, et al. Haplotype estimation for biobank-scale data sets. *Nature genetics* 2016;48(7):817.
9. Huang J, Howie B, McCarthy S, et al. Improved imputation of low-frequency and rare variants using the UK10K haplotype reference panel. *Nature Communications* 2015;6:8111.
10. Howie B, Marchini J, Stephens M. Genotype Imputation with Thousands of Genomes. *G3 Genes|Genomes|Genetics* 2011;1(6):457-70. doi: 10.1534/g3.111.001198
11. Manichaikul A, Mychaleckyj JC, Rich SS, et al. Robust relationship inference in genome-wide association studies. *Bioinformatics* 2010;26(22):2867-73.

For peer review only

For peer review only - http://bmjopen.bmj.com/site/about/guidelines.xhtml

12. Kurki MI, Karjalainen J, Palta P, et al. FinnGen provides genetic insights from a well-phenotyped isolated population. *Nature* 2023;613(7944):508-18. doi: 10.1038/s41586-022-05473-8

13. FinnGen. Documentation of R12 release 2024 [Available from: https://finngen.gitbook.io/documentation/ accessed 2025-03-10.

14. PLINK [program]. 2.00 alpha (build 22 Dec 2024) version, 2024.

15. Chang CC, Chow CC, Tellier LC, et al. Second-generation PLINK: rising to the challenge of larger and richer datasets. *Gigascience* 2015;4(1):7.

16. Firth D. Bias reduction of maximum likelihood estimates. *Biometrika* 1993;80(1):27-38. doi: 10.1093/biomet/80.1.27

17. Mbatchou J, Barnard L, Backman J, et al. Computationally efficient whole-genome regression for quantitative and binary traits. *Nature Genetics* 2021;53(7):1097-103. doi: 10.1038/s41588-021-00870-7

18. Abraham G, Qiu Y, Inouye M. FlashPCA2: principal component analysis of Biobank-scale genotype datasets. *Bioinformatics* 2017;33(17):2776-78. doi: 10.1093/bioinformatics/btx299

19. Abraham G. FlashPCA2 2016 [Available from: https://github.com/gabraham/flashpca accessed 2025_03-11.

20. Lawson D. R package pcapred 2020 [Available from: https://github.com/danjlawson/pcapred accessed 2025-03-10.

21. Ani A, van der Most PJ, Snieder H, et al. GWASinspector: comprehensive quality control of genome-wide association study results. *Bioinformatics* 2021;37(1):129-30. doi: 10.1093/bioinformatics/btaa1084

22. Beaumont RN, Flatley C, Vaudel M, et al. Genome-wide association study of placental weight in 179,025 children and parents reveals distinct and shared genetic influences between placental and fetal growth. *medRxiv* 2022:2022.11.25.22282723. doi: 10.1101/2022.11.25.22282723

23. Warrington NM, Beaumont RN, Horikoshi M, et al. Maternal and fetal genetic effects on birth weight and their relevance to cardio-metabolic risk factors. *Nature genetics* 2019:1.

For peer review only

For peer review only - http://bmjopen.bmj.com/site/about/guidelines.xhtml

24. Warrington NM, Hwang L-D, Nivard MG, et al. Estimating direct and indirect genetic effects on offspring phenotypes using genome-wide summary results data. *Nature Communications* 2021;12(1):5420. doi: 10.1038/s41467-021-25723-z

25. Wu Y, Zhong X, Lin Y, et al. Estimating genetic nurture with summary statistics of multigenerational genome-wide association studies. *Proceedings of the National Academy of Sciences* 2021;118(25):e2023184118.

26. Bulik-Sullivan B, Finucane HK, Anttila V, et al. An atlas of genetic correlations across human diseases and traits. *Nature Genetics* 2015;47(11):1236-41. doi: 10.1038/ng.3406

27. Bond TA, Bhatta L, Yang Q, et al. Parental body mass index and offspring cardiovascular risk factors in adulthood: an intergenerational Mendelian randomization study. *medRxiv* 2025:2025.05.06.25327111. doi: 10.1101/2025.05.06.25327111

For peer review only

For peer review only - http://bmjopen.bmj.com/site/about/guidelines.xhtml

The Mendelian Randomization in Pregnancy (MR-PREG) collaboration: Improving evidence on prevention, prediction and treatment of adverse pregnancy and perinatal outcomes

Supplementary figures

Avon Longitudinal Study of Parents and Children

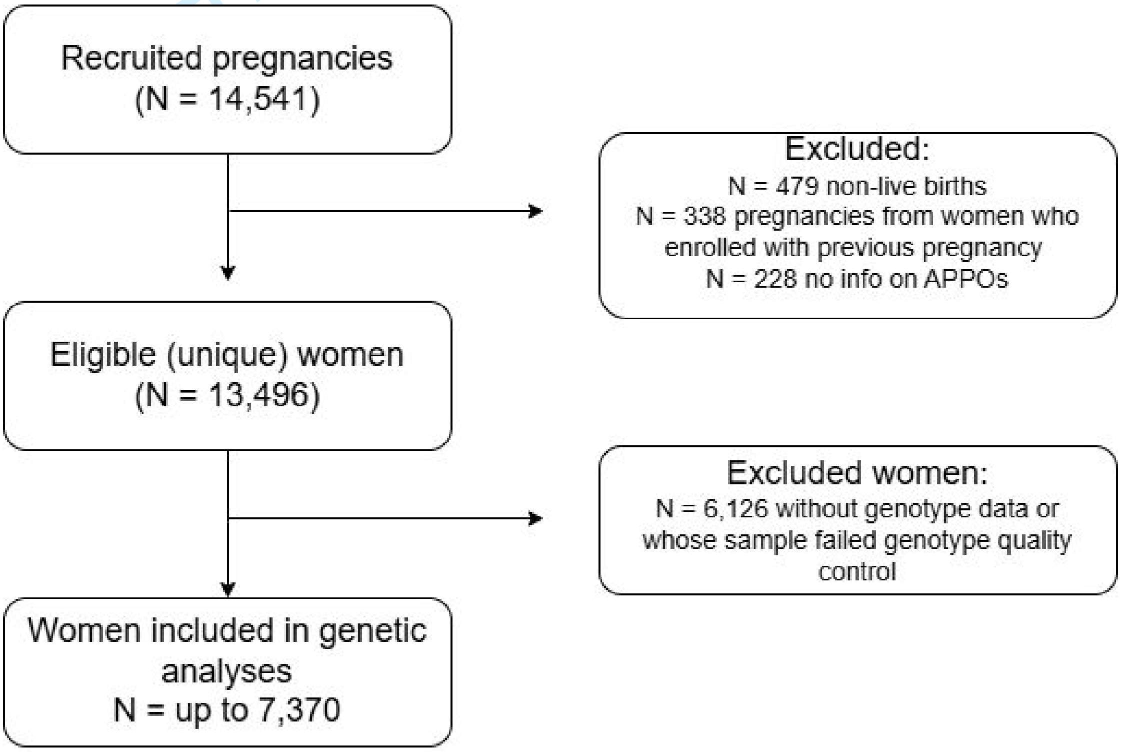

Genetic data is available for a subsample of mothers

Born in Bradford

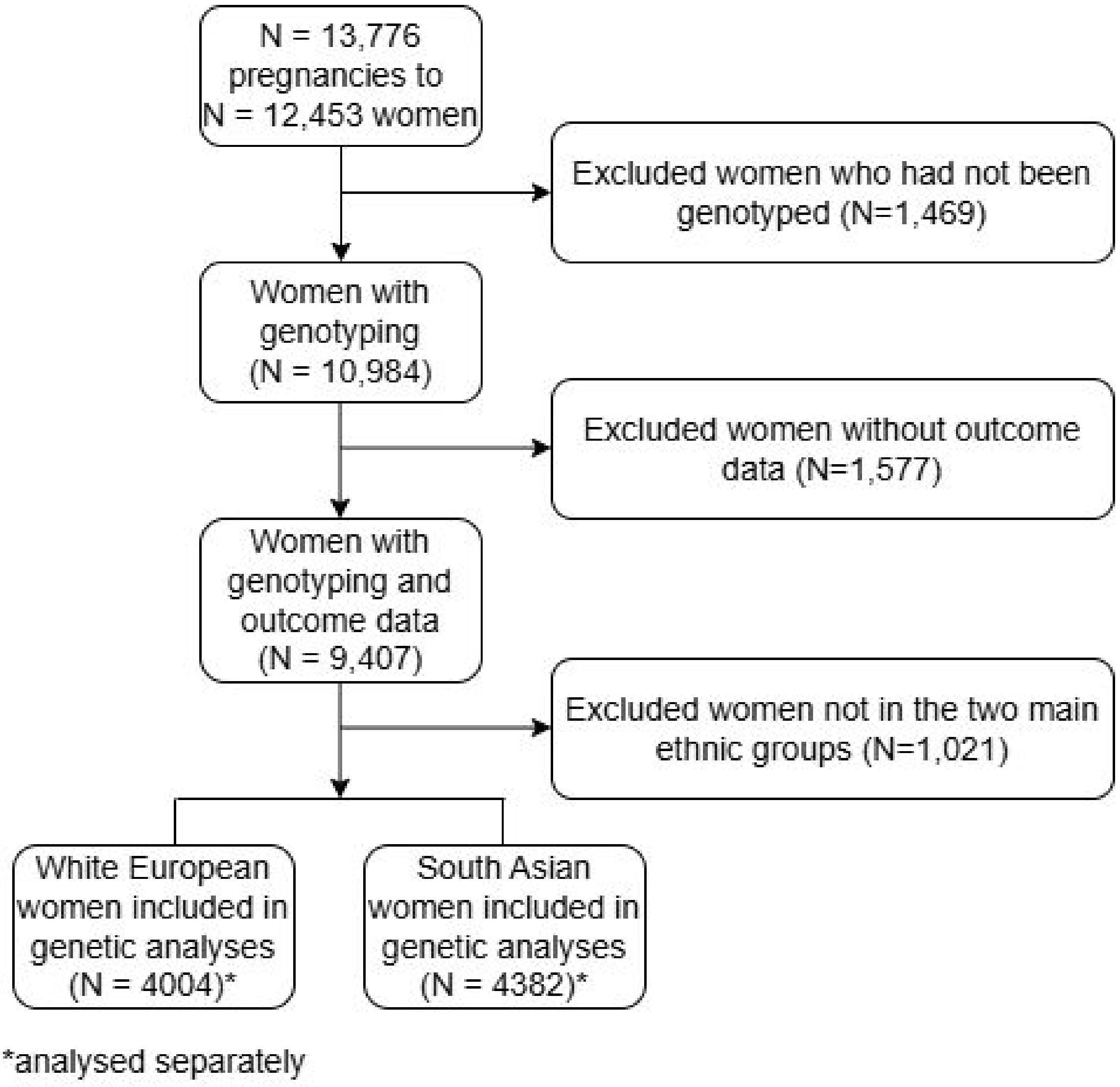

Norwegian Mother Father and Child Birth Cohort

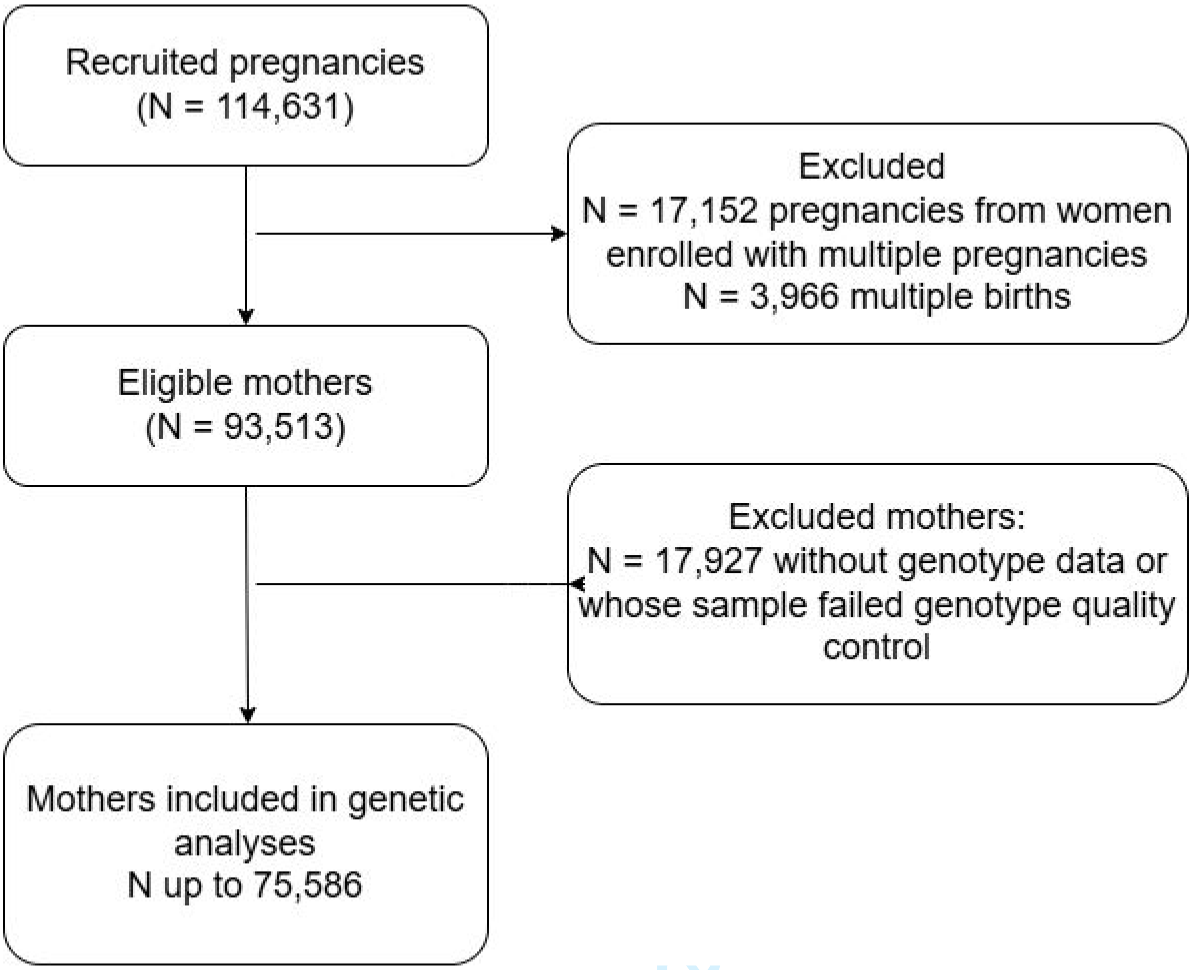

ew only

UK Biobank

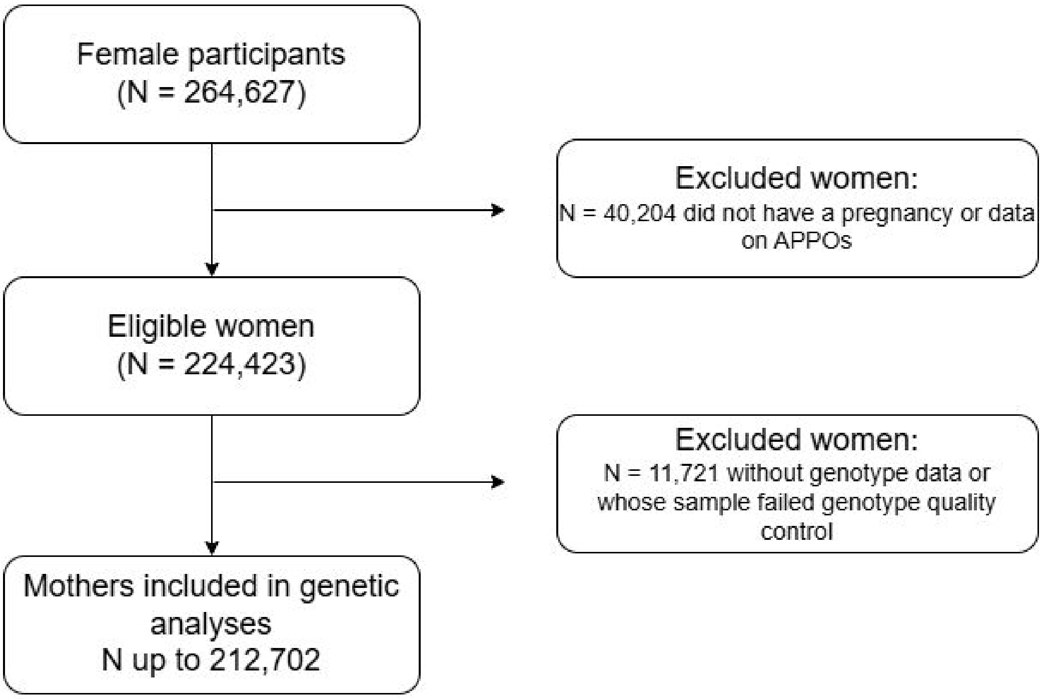

Note: some phenotypes have much smaller sample size because the source of information was maternity records (N up to 8,494), and these were not available for all women

Supplementary Tables

**ST1.**	Supplementary Table 1
**ST2A.**	Supplementary Table 2A
**ST2B.**	Supplementary Table 2B
**ST3A.**	Supplementary Table 3A
**ST3B.**	Supplementary Table 3B
**ST3C.**	Supplementary Table 3C
**ST3D.**	Supplementary Table 3D
**ST3E.**	Supplementary Table 3E
**ST4A.**	Supplementary Table 4A
**ST4B.**	Supplementary Table 4B
**ST5.**	Supplementary Table 5

For peer review only

Summary of approaches that each have different assumptions that can help determine the causality of risk fac
Overview of definitions of binary APPOs (N = 33) and contributing studies
Overview of definitions of continuous (N = 2) and categorical (N = 2) APPOs and contributing studies
Number of cases and controls per APPO by each contributing study
Number of cases and controls - that contribute to maternal genetic analyses - per APPO by each contributing s
Number of cases and controls - that contribute to offspring and paternal genetic analyses - per APPO by each c
Overall N, mean and SD for continuous APPOs - split by overall N and N with maternal genotype by each contri
Overall N, mean and SD for continuous APPOs with offspring and paternal genotype by each contributing stud
Summary of any deviations from the MR PREG (binary) APPO definition by each contributing study
Summary of any deviations from the MR PREG (continuous) APPO definition by each contributing study
Summary of the publicly available GWAS that were used for particular outcomes and the overlap with core MF

For peer review only

ctors on APPOs

study
contributing study
ibuting study
y

R PREG studies

For peer review only

Type of study design/approach	Key assumptions
**Conventional multivariable regression**	
To estimate the effect of a potential risk factor on adverse pregnancy outcomes (APPOs) by adjusting for observed confounders	All confounders are observed, correctly measured and modelled<br>No selection / measurement bias
**Negative control (exposure or outcome) study**	
To detect residual confounding or other biases by testing associations between the exposure and an outcome that should theoretically not be affected by the exposure, e.g., paternal factors which could not influence the maternal intrauterine environment	No plausible 'biological'/causal relationship between control exposure and outcome (or between exposure and control outcome)<br>Same biases (eg confounding) for negative control exposure/outcome as for main research question
**Mendelian randomisation (MR)**	
Uses genetic variants, which are randomly assigned at conception, to improve causal inference for modifiable exposures on APPOs	Strong relevant genetic instruments (ie genetic variants strongly associated with exposure in the relevant population)<br>No horizontal pleiotropy (when genetic variants affect the outcome through pathways other than the exposure)<br>No confounding of the variant-outcome association (e.g. population stratification)<br>No selection bias or measurement error
**Cross-context comparisons**	
To assess causal effects by examining if relationships hold across different populations/time periods/contexts	Marked differences in confounders between contexts (populations), not just in their prevalence<br>No effect modification by context/population
**Within-family analyses**	
Comparing siblings with a differential exposure can control for genetic and family-level confounding (as siblings share many genetic and environmental factors)	Within sibling pairs, observed and unobserved confounders are matched<br>No or little individual confounding<br>Little individual measurement error<br>No crossover or selection bias
**Natural experiment**	

To assess causal effects by using an external factor (e.g., policy change) as a quasi-random assignment of exposure	Assumes the external factor occurs randomly - i.e., the event or intervention (e.g., a new law or a natural disaster should be unrelated to the participants' characteristics or outcomes before it occurred)<br>Assumes random assignment<br>Assumes no confounding from differences in populations exposed versus unexposed
**Target trial emulation**	
To mimic a randomized controlled trial (RCT) by using observational data, emulating eligibility criteria, treatment strategy/assignment, time-zero, follow-up, outcome measurement, and causal contrast as if it were an RCT	Assumes that causal inference can be improved by designing and analysing an observational study to mimic, as closely as possible, the design and analyses of a real trial
**Drug target MR**	
Used to assess the causal effect of a drug target on disease outcomes by leveraging genetic variants as proxies, providing insights into the potential efficacy and safety of drug interventions	Assumes that genetic variants influencing the target gene or protein act as valid instruments, meaning they affect the disease outcome only through the drug target, not through other pathways

For peer review only

Rationale	Example
Quick and efficient insights through observational data	Effect of pre-pregnancy body mass index (BMI) on APPOs
Reveals existence of potential unmeasured confounding<br><br>Can be extended to explore other types of bias (e.g., recall bias, selection bias)<br>Efficient insights through use of data already collected	Main hypothesis: Effect of pre-pregnancy BMI on APPOs; Negative control exposure: Effect of paternal BMI on APPOs. If an association is found between paternal BMI and APPOs, this could indicate, for example, residual confounding in the main analysis
Less prone to reverse causation<br><br>Less prone to residual or unmeasured confounding	Effect of hyperglycaemia during pregnancy (instrumented by genetic variants) on APPOs
Can reveal context-specific confounding, selection bias or measurement error<br><br>Can examine the influence of high-level socioeconomic factors which are difficult to measure and fully account for	Breastfeeding and offspring health outcomes (e.g., blood pressure) across contexts (i.e., countries which have a very different socioeconomic pattern of breastfeeding)
Adjustment for a wide range of shared familial and parental characteristics<br><br>Control for genetic confounding (if identical twins)	The effect of assisted reproductive technologies (ART) vs natural conception on APPOs

For peer review only

When a natural event randomly affects some groups but not others (like a sudden policy change in one region but not another), it can help mimic randomisation, making comparisons between groups more reliable.<br><br>Can use existing real world data	Effect of air pollution on APPOs using implementation of low emission zone as natural experiment
Can answer key public health questions where a real trial might be too costly, too long or ethically impossible<br>Can avoid immortal time bias (i.e., when bias occurs due to including periods of time where people must survive to be included) and prevalent user bias	The effect (efficacy and safety) of hypoglycaemics use during pregnancy on APPOs
Can predict the potential intended and unintended on-target effects of drugs, aiding in drug development and prioritization	The on-target effect of statins on pre-term birth

For peer review only

Type	Outcomes	Contributing studies[1]					
		A	B	M	U	F	G*
Pregnancy	Hypertensive disorders of pregnancy	x	x	x			
					x	x	
	Gestational hypertension	x	x	x			
					x	x	
	Pre-eclampsia	x	x	x			x
					x	x	x
	Gestational diabetes			x	x		x
		x					x
					x	x	x
			x				x
	Perinatal depression	x					x
			x				x
					x		x
				x			
	Anaemia	x					
				x			
			x				
	Pregnancy loss	x	x	x	x		
	Miscarriage	x		x	x		
			x		x	x	
	Sporadic miscarriage	x		x	x		
	Recurrent miscarriage	x		x	x	x	
	Stillbirth	x			x		
			x	x			
	Hyperemesis			x			
		x	x		x	x	
	Induction of labor	x	x	x	x		

review only

**Delivery**	Premature rupture of membranes	x		x		x	
	Caesarian section	x	x	x	x	x	
	Emergency C-section	x	x	x	x		
	Elective C-section	x	x	x	x		
	Very pre-term birth	x	x	x	x		
	Pre-term birth	x	x	x	x		x
	Spontaneous pre-term birth		x	x	x		x
	Post-term birth	x	x	x	x	x	x
	Low birth weight	x	x	x	x		
	High birth weight	x	x	x	x		
	Small for gestational age (SGA)	x	x	x	x		
	Large for gestational age (LGA)	x	x	x	x		
	Low Apgar score at 1 minute	x	x	x			
	Low Apgar score at 5 minutes	x	x	x			
**Postnatal**	NICU admission	x		x			
	Congenital anomalies			x			
	Congenital heart disease			x			
	Breastfeeding initiation	x	x	x			
	Breastfeeding established	x	x	x			
	Breastfeeding sustained	x	x	x			

[1] Contributing studies: ALSPAC (A), BiB (B), MoBa (M), UK Biobank (U), FinnG
[2] Pavord S et al. UK guidelines on the management of iron deficiency in preg
* Definition of cases and controls in the publicly available GWAS might vary

review only

For peer review only - http://bmjopen.bmj.com/site/about/guidelines.xhtml

Case definition	Control definition
Gestational hypertension or pre-eclampsia<br>ICD-10 codes: O13, O14	No hypertensive disorders of pregnancy
SBP ≥ 140 mmHg or DBP ≥ 90 mmHg on at least 2 occasions after 20 gestational weeks, with no concurrent proteinuria<br>ICD-10 code: O13	
Gestational hypertension with concurrent proteinuria<br>ICD-10 code: O14	
Self-reported diabetes arising in pregnancy<br>Clinical diagnoses abstracted from medical records by research midwives<br>ICD-10 code: O24<br>Fasting glucose ≥ 6.1 mmol/L or 2 h post-load glucose ≥ 7.8 mmol/L in OGTT	No gestational diabetes
Mean EPDS total score across 12 and 32 gestational weeks and 8 weeks and 8 months postpartum > 12<br>Mean score of questions 22 to 28 in the GHQ administered at cohort registration during pregnancy > the 85th percentile<br>Self-reported antenatal depression<br>Mean of selective items from the (Hopkins) Symptoms Checklist-25 at 14 and 30 weeks (and 6 months post partum)>=2	No depression
Maternal haemoglobin (Hb) < 100 g/L during pregnancy<br>Self reported anaemia/low haemoglobin at 15 weeks gestation<br>Clinical diagnosis abstracted from medical records	No maternal anaemia
Miscarriage or stillbirth in the index or previous pregnancies	No pregnancy loss
Miscarriage in the index or previous pregnancies; miscarriage in the index pregnancy was defined as fetal loss before 20 weeks gestation; miscarriage history in previous pregnancies was self-reported in questionnaires<br>Miscarriage in the index or previous pregnancies; information on miscarriage was abstracted from medical records	
≤ 2 miscarriages in the index and previous pregnancies	
≥ 3 miscarriages in the index and previous pregnancies	
Stillbirth in the index or previous pregnancies; stillbirth in the index pregnancy was defined as fetal death before delivery at 20 gestational weeks or more; stillbirth history in previous pregnancies was self-reported in questionnaires<br>Stillbirth in the index or previous pregnancies; information on stillbirth was abstracted from medical records	
Self reported: excessive vomiting noted during pregnancy before onset of labour<br>Clinical diagnosis abstracted from medical records	No hyperemesis
Medical induction needed during labour	No induction of labour

For peer review only

Premature rupture of membranes at any gestational age	No premature rupture of membranes
Had a C-section	No C-section
Had an emergency C-section	
Had an elective C-section	
Gestational age at birth < 34 weeks	Gestational age at birth ≥ 37 to < 42 weeks
Gestational age at birth < 37 weeks	
Gestational age at birth < 37 weeks	
Gestational age at birth ≥ 42 weeks	
Birth weight < 2500 g	Birth weight ≥ 2500 g to ≤ 4500 g
Birth weight > 4500 g	
Birth weight-for-gestational age < 10th percentile	No SGA
Birth weight-for-gestational age > 90th percentile	No LGA
Apgar score at 1 minute < 7	Apgar at 1 minute ≥7
Apgar score at 5 minutes < 7	Apgar at 5 minutes ≥7
Admission to neonatal intensive care unit (NICU)	No NICU admission
Congenital malformations from the medical birth registry	No congenital anomalies
Congenital malformations (heart condition) from the medical birth registry	No congenital heart disease
Breastfeeding initiated	No breastfeeding
Breastfeeding ≥ 2 months	Breastfeeding < 2 months
Breastfeeding ≥ 6 months	Breastfeeding < 6 months

ʒen (F), multiple publicly available GWAS (G)
ʒnancy. Br J Haematol. 2020;188(6):819-830.
across studies contributing to the GWAS metanalyses

For peer review only

Exclusions		
**Multiple births**	**Duplicated pregnancies**	**Non-live births**
x	x	x
x	x	x
x	x	x
x	x	x
x	x	x
x	x	x
x	x	
x	x	
x	x	
x	x	
x	x	
x	x	x
x	x	x

For peer review only

x	x	x
x	x	x
x	x	x
x	x	x
x	x	x
x	x	x
x	x	x
x	x	x
x	x	x
x	x	x
x	x	x
x	x	x
x	x	x
x	x	x
x	x	x
x	x	x
x	x	x
x	x	x
x	x	x
x	x	x

For peer review only

**Other**
Pre-existing hypertension
Pre-existing diabetes
Pre-existing depression
Age menarche < 9 or > 17 y and some specific medical conditions

For peer review only

Emergency c-section, labour induction
Never breastfed, preterm birth

For peer review only

Type	Outcomes	Contributing studies[1]					
		A	B	M	U	F	G*
**Pregnancy**	Severity of nausea and vomiting	x		x			
**Delivery**	Birth weight	x	x	x			
					x		
	Gestational age at birth	x	x	x	x		x
**Postnatal**	Breastfeeding duration	x	x	x			

[1] Contributing studies: ALSPAC (A), BiB (B), MoBa (M), UK Biobank (U), FinnGen (F), mu

* Outcome definition in the publicly available GWAS might vary across studies contrib

For peer review only

Source	Unit/definition
Self-reported in questionnaires	0 = None; 1 = Nausea only; 2 = Nausea and vomiting; 3 = On medication
Abstracted from medical records	SD units (Z-score transformed separately in males and females)
Self-reported in questionnaires	
Abstracted from medical records	Weeks
Self-reported in questionnaires	0 = never breasfed or < 1 month; 1 = 1-3 months; 2 = 4-6 months; 3 > 6 months

ultiple publicly available GWAS (G)
uting to the GWAS metanalyses

For peer review only

Exclusions			
Multiple births	Duplicated pregnancies	Non-live births	Other
x	x	x	
x	x	x	Birth weight > 5 SD
x	x	x	
x	x	x	

For peer review only

Type	Outcomes	Overall		ALSPAC		B
		Cases	Controls	Cases	Controls	Cases
Pregnancy	Hypertensive disorders of pregnancy	15,485	278,472	2,148	10,692	764
	Gestational hypertension	11,134	278,971	1,865	10,865	563
	Pre-eclampsia	4,428	278,969	283	10,692	201
	Gestational diabetes	2,772	333,614	55	11,835	765
	Depression	12,669	122,588	772	9,923	972
	Anemia	4,354	102,045	1,001	7,013	1,850
	Pregnancy loss	104,412	230,957	3,155	9,696	185
	Miscarriage	73,462	230,957	3,045	9,696	44
	Sporadic miscarriage	61,900	209,622	2,069	9,696	
	Recurrent miscarriage	6,007	209,732	153	9,696	
	Stillbirth	7,340	230,957	170	9,696	142
	Hyperemesis	1,298	274,857	81	7,933	84
	Severity of nausea and vomiting		16,401		2,113	
	Nausea only	9,123		3,540		
	Nausea and vomiting	34,776		5,946		
	On medication	33,998		1,122		
Delivery	Induction of labor	18,098	100,985	1,502	6,359	1,961
	Premature rupture of membranes	15,011	85,761	2,143	5,630	
	Caesarean section	19,518	99,935	1,383	6,491	2,025
	Emergency C-section	11,749	101,182	890	6,491	974
	Elective C-section	7,497	101,226	493	6,491	644
	Very pre-term birth	1,672	106,882	196	11,769	127
	Pre-term birth	6,227	106,814	695	11,769	490
	Post-term birth	8,893	106,774	1,032	11,769	138
	Low birth weight	4,357	115,392	605	12,490	669
	High birth weight	4,494	115,392	232	12,490	92
	Small for gestational age	9,729	112,726	1,058	12,265	1,302
	Large for gestational age	13,102	109,353	1,466	11,857	703
	Low Apgar score at 1 minute	6,537	102,995	990	6,846	772
	Low Apgar score at 5 minutes	1,290	108,248	125	7,708	115
Postntal	NICU admission	9,170	90,404	753	11,035	
	Congenital anomalies	4,582	88,032			
	Congenital heart disease	797	91,817			
	Breastfeeding initiation	82,785	25,833	9,189	2,707	2,255
	Breastfeeding established	71,564	31,296	5,897	1,537	1,112
	Breastfeeding sustained	54,481	26,208	3,490	3,944	673
	Breastfeeding duration		29,423		1,079	
	1 to 3	8,417		2,077		98
	4 to 6	61,547		1,636		29
	>6	4,761		2,979		1,782

Note: These are the number of participants with phenotype data for the core studies only; not all cases and c

* In the MoBa study, information on breastfeeding duration beyond 6 months was not available.

iB	MoBa		UK Biobank	
Controls	Cases	Controls	Cases	Controls
6,831	11,669	80,774	904	180,175
6,831	8,129	80,774	577	180,501
6,831	3,540	80,774	404	180,672
8,233	803	91,356	1,149	222,190
6,033	4,649	80,190	6,276	26,442
6,169	1,503	88,863		
7,847	16,589	76,924	84,483	136,490
7,847	16,152	76,924	54,221	136,490
	15,046	76,610	44,785	123,316
	1,028	76,610	4,826	123,426
7,847	292	76,924	6,736	136,490
7,944	834	76,382	299	182,598
		14,288		
	5,583			
	28,830			
	32,876			
7,384	12,370	80,224	2,265	7,018
	12,868	80,131		
7,327	13,485	79,299	2,625	6,818
7,396	8,454	79,299	1,431	7,996
7,396	4,973	79,299	1,387	8,040
8,724	1,238	80,366	111	6,023
8,724	4,571	80,366	471	5,955
8,724	7,274	80,366	449	5,915
8,590	2,697	85,769	386	8,543
8,590	3,974	85,769	196	8,543
8,046	6,801	85,272	568	7,143
8,645	9,690	82,383	1,243	6,468
8,473	4,775	87,676		
9,127	1,050	91,413		
	8,417	79,369		
	4,582	88,032		
	797	91,817		
955	71,341	22,171		
802	64,555	28,957		
1,241	50,318	21,023		
955		27,389		
	6,242			
	59,882			
	NA*			

controls might have genetic data

For peer review only

Type	Outcomes	Overall		ALSPAC		B
		Cases	Controls	Cases	Controls	Cases
Pregnancy	Hypertensive disorders of pregnancy	32,549	509,219	1,187	5,851	683
	Gestational hypertension	20,777	507,155	1,029	5,851	499
	Pre-eclampsia	19,408	652,584			184
	Gestational diabetes	24,641	683,156			
	Depression	14,591	91,065			839
	Anemia	4,105	82,062	559	3,901	1,597
	Pregnancy loss	94,152	204,716	1,524	5,470	167
	Miscarriage	89,086	397,131	1,477	5,470	
	Sporadic miscarriage	55,888	185,271	1,156	5,470	
	Recurrent miscarriage	6,233	315,523	90	5,470	
	Stillbirth	6,331	201,339	70	5,470	87
	Hyperemesis	4,235	474,231			
	Severity of nausea and vomiting		14,469		1,177	
	Nausea only	7,166		1,972		
	Nausea and vomiting	30,133		3,312		
	On medication	31,210		625		
Delivery	Induction of labor	14,495	80,744	849	3,538	1,686
	Premature rupture of membranes	21,839	288,782	1,212	3,127	
	Caesarean section	33,909	200,060	757	3,636	1,700
	Emergency C-section	9,165	81,273	481	3,636	836
	Elective C-section	6,004	81,279	276	3,636	561
	Very pre-term birth	1,107	76,576	82	6,440	
	Pre-term birth	18,225	267,497			414
	Spontaneous pre-term birth	17,176	255,764			203
	Post-term birth	27,268	391,392			126
	Low birth weight	19,180	263,971	293	6,858	563
	High birth weight	6,679	260,156	123	6,858	64
	Small for gestational age	7,448	88,857	566	6,706	1,114
	Large for gestational age	10,468	85,837	811	6,461	626
	Low Apgar score at 1 minute	4,950	81,718	544	3,831	628
	Low Apgar score at 5 minutes	821	73,547			
Postnatal	NICU admission	6,996	70,289	408	6,327	
	Congenital anomalies	3,569	71,132			
	Congenital heart disease	612	74,089			
	Breastfeeding initiation	66,408	18,260	5,485	1,307	1,977
	Breastfeeding established	58,035	23,223	3,698	859	962
	Breastfeeding sustained	44,401	20,788	2,254	2,303	556
	Breastfeeding duration		23,515		660	
	1 to 3	6,372		1,270		88
	4 to 6	49,123		1,000		26
	>6	3,419		1,821		1,598

Note: These are the numbers of participants with both phenotype and maternal genetic data across all cor
variants, the reported number of participants corresponds to the median sample size and may therefore d
a given APPO were excluded if the number of cases was lower than 50 or if the study had already been inc

For peer review only

iB	MoBa		UK Biobank		FinnGen		GWAS	
Controls	Cases	Controls	Cases	Controls	Cases	Controls	Cases	Controls
5,965	9,472	64,878	802	170,866	20,405	261,659		
5,965	6,681	64,877	518	171,149	12,050	259,313		
5,965	2,628	60,335	354	171,311	9,023	259,313	7,219	155,660
	602	73,507			18,581	263,483	5,458	346,166
5,312	2,889	52,282					10,863	33,471
5,421	1,949	72,740						
6,865	13,451	61,563	79,010	130,818				
	13,116	61,564	51,326	130,818	23,167	199,279		
	12,227	61,336	42,505	118,465				
	832	61,336	4,500	118,557	811	130,160		
3,488	223	61,563	5,951	130,818				
	656	63,115	250	173,210	3,329	237,906		
	5,194	13,292						
	26,821							
	30,585							
6,333	9,915	64,563	2,045	6,310				
	10,219	64,469			10,408	221,186		
6,324	10,582	64,019	2,303	6,191	18,567	119,890		
6,382	6,605	64,019	1,243	7,236				
6,382	3,929	64,020	1,238	7,241				
	940	64,670	85	5,466				
7,484	2,892	48,159	406	5,403			14,513	206,451
4,820	2,206	40,359	254	4,134			14,513	206,451
7,484	4,543	48,160	424	5,360	7,049	221,186	15,126	109,202
7,374	2,038	69,079	16,286	180,660				
3,560	3,244	69,078	3,248	180,660				
6,907	5,303	68,765	465	6,479				
7,395	7,865	66,203	1,166	5,778				
7,302	3,778	70,585						
	821	73,547						
	6,588	63,962						
	3,569	71,132						
	612	74,089						
885	58,946	16,068						
725	53,375	21,639						
1,131	41,591	17,354						
856	5,014	21,999						
	48,097							

ntributing studies. For GWASes where there was variation in the sample size across genetic
liffer from numbers reported elsewhere (e.g. in publicly available GWAS). Study-specific results for
:luded by publicly available GWAS (see Supplementary Table 5).

For peer review only

Type	Outcomes	Overall		ALSPAC		B
		Offspring		Offspring		Offs
		Cases	Controls	Cases	Controls	Cases
**Pregnancy**	Hypertensive disorders of pregnancy	9,653	64,516	1,215	6,116	517
	Gestational hypertension	7,227	64,516	1,060	6,116	382
	Pre-eclampsia	6,893	433,018	155	5,852	127
	Gestational diabetes	990	66,772			510
	Depression	3,795	65,973	399	5,901	586
	Anemia	3,439	67,875	533	3,910	1,241
	Pregnancy loss	12,988	58,567	1,566	5,486	68
	Miscarriage	12,698	55,988	1,531	5,532	
	Sporadic miscarriage	11,871	55,989	1,432	5,532	
	Recurrent miscarriage	822	55,989	95	5,532	
	Stillbirth	389	71,167	61	6,992	53
	Hyperemesis	484	42,872			
	Severity of nausea and vomiting					
	Nausea only	6,019	11,505	1,987	1,186	
	Nausea and vomiting	24,158		3,337		
	On medication	24,373		630		
**Delivery**	Induction of labor	10,094	62,012	840	3,535	1,254
	Premature rupture of membranes	9,724	56,207	1,218	3,116	
	Caesarean section	9,592	62,574	740	3,640	1,447
	Emergency C-section	5,594	62,590	460	3,640	637
	Elective C-section	3,647	62,590	280	3,640	490
	Very pre-term birth	492	60,574	80	6,484	
	Pre-term birth	7,326	112,313			259
	Spontaneous pre-term birth	2,003	52,433			136
	Post-term birth	12,883	109,442			50
	Low birth weight	1,799	70,252	260	6,900	357
	High birth weight	2,919	67,058	130	6,900	53
	Small for gestational age	5,391	69,381	512	6,776	823
	Large for gestational age	7,988	66,784	852	6,436	495
	Low Apgar score at 1 minute	3,647	68,352	508	3,854	438
	Low Apgar score at 5 minutes	492	61,000			
**Postnatal**	NICU admission	4,993	60,080	382	6,502	
	Congenital anomalies	2,665	58,824			
	Congenital heart disease	439	61,050			
	Breastfeeding initiation	58,700	12,017	5,611	1,324	1,502
	Breastfeeding established	51,357	16,290	3,849	904	732
	Breastfeeding sustained	39,199	18,412	2,359	2,394	433
	Breastfeeding duration					
	1 to 3	5,508	15,908	1,323	687	66
	4 to 6	40,573		1,042		19
	>6	3,096		1,898		1,198

Note: These are the numbers of participants with both phenotype and fetal genetic data across contributi genetic variants, the reported number of participants corresponds to the median sample size and may the GWAS). Study-specific results for a given APPO were excluded if the number of cases was lower than 50 or Supplementary Table 5).

iB	MoBa				GWAS	
spring	Offspring		Fathers		Offspring	
Controls	Cases	Controls	Cases	Controls	Cases	Controls
4,766	7,921	53,634	6,476	42,973		
4,766	5,785	53,634	4,546	42,972		
4,529	1,981	49,292	1,930	42,876	4,630	373,345
5,655	480	61,117	391	49,127		
3,918	2,810	56,154	2,075	44,092		
4,033	1,665	59,932	1,198	48,370		
2,812	11,354	50,269	8,852	40,926		
	11,167	50,456	8,719	41,059		
	10,439	50,457	8,167	41,059		
	727	50,457	552	41,060		
2,827	275	61,348	193	49,585		
			484	42,872		
	4,032	10,319	3,167	8,106		
	20,821		16,355			
	23,743		18,651			
4,932	8,000	53,545	6,784	42,649		
	8,506	53,091	7,190	42,378		
4,742	7,405	54,192	7,174	42,344		
4,758	4,497	54,192	4,569	42,343		
4,758	2,877	54,192	2,579	42,344		
	412	54,090	691	42,819		
5,845	2,292	54,090	2,424	42,819	4,775	52,378
3,673	1,724	46,083	1,823	36,006	4,775	52,378
2,546	4,945	54,089	3,991	42,818	7,888	52,807
5,758	1,182	57,594	1,410	45,904		
2,564	2,736	57,594	2,046	45,904		
5,365	4,056	57,240	3,545	45,619		
5,693	6,641	54,655	5,071	44,093		
5,716	2,701	58,782	2,564	46,795		
	492	61,000	564	48,803		
	4,611	53,578	4,606	42,835		
	2,665	58,824	2,448	47,140		
	439	61,050	430	49,158		
657	51,587	10,036	39,683	10,095		
539	46,776	14,847	35,918	13,861		
838	36,407	15,180	30,075	9,607		
642	4,119	18,072	3,323	14,579		
	39,512		31,876			

ng studies. For GWASes where there was variation in the sample size across
erefore differ from numbers reported elsewhere (e.g. in publicly available
r if the study had already been included by publicly available GWAS (see

For peer review only

Type	Outcomes	Overall				
		N phenotype	N genotype	Mean	SD	N phenotype
Delivery	Birth weight (grams)	328,256	289,846	3376.7	601.9	13,354
	Gestational age (weeks)	126,151	189,471	39.5	1.9	13,496

Note: These are the numbers of participants with both phenotype and maternal genetic data across all contribu
in publicly available GWAS). Study-specific genotype results for a given APPO were excluded if the study had alr

For peer review only

ALSPAC			BiB				MoB	
N genotype	Mean	SD	N phenotype	N genotype	Mean	SD	N phenotype	N genotype
7,273	3408.9	555	12,346	8,023	3225.9	557	92,440	74,361
	39.4	1.9	12,346		39.5	1.8	92,211	55,595

uting studies. For GWASes where there was variation in the sample size across genetic variants, the reported number of pa
ready been included by publicly available GWAS (see Supplementary Table 5).

For peer review only

3a		UK Biobank				GWAS		
Mean	SD	N phenotype	N genotype	Mean	SD	N	Mean	SD
3596	558	210,116	200,189	3239.4	640			
39.5	1.9	8,098	7,303	39.5	1.7	126,573		

articipants corresponds to the median sample size and may therefore differ from numbers reported elsewhere (e.g.

For peer review only

Type	Outcomes	Overall	BiB	MoBa		GWAS
		N offspring	N offspring	N offspring	N fathers	N offspring
Delivery	Birth weight (grams)	345,022	5,930	59,221	46,978	279,871
	Gestational age (weeks)	152,204	6,189	61,326	49,233	84,689

Note: These are the numbers of participants with both phenotype and fetal genetic data across contributing studies. For GWASes where there was variation in the sample size across genetic variants, the reported number of participants corresponds to the median sample sample size and may therefore differ from numbers reported elsewhere (e.g. in publicly available GWAS).

For peer review only

Type	Outcomes	ALSPAC
Pregnancy	Gestational hypertension	-
	Pre-eclampsia	-
	Hypertensive disorders of pregnancy	-
	Gestational diabetes	-
	Hyperemesis	-
	Severity of nausea and vomiting	-
	Depression	-
	Anemia	-
	Pregnancy loss	-
	Miscarriage	-
	Sporadic miscarriage	-
	Recurrent miscarriage	-
	Stillbirth	-
	Induction of labor	-
	Premature rupture of membranes	-

For peer review only

Delivery	Caesarean section	-
	Emergency C-section	-
	Elective C-section	-
	Very pre-term birth	-
	Pre-term birth	-
	Spontaneous pre-term birth	-
	Post-term birth	-
	Low birth weight	-
	High birth weight	-
	Small for gestational age	-
	Large for gestational age	-
	Low Apgar score at 1 minute	-
	Low Apgar score at 5 minutes	-
	NICU admission	-

For peer review only

**Postnatal**	Congenital anomalies	NA
	Congenital heart disease	NA
	Breastfeeding initiation	-
	Breastfeeding established	-
	Breastfeeding sustained	-
	Breastfeeding duration	-

Note: - no deviations from MR-PREG definition; NA: not available

For peer review only

Deviations from the collaboration definition		
BiB	MoBa	UK Biobank
-	-	-
-	-	-
-	-	-
-	-	-
-	-	-
NA	-	NA
Only antenatal depression available, derived from GHQ; pre-existing depression not excluded	-	Depression possibly related to childbirth, based on self-report
-	-	NA
-	-	-
-	Self report cases defined as pregnancy loss before 23 weeks gestation (previous not index)	Self-reported cases might not be pregnancy loss at < 22 gestational weeks; no index pregnancy
NA	-	Self-reported cases might not be pregnancy loss at < 22 gestational weeks; no index pregnancy
NA	-	Self-reported cases might not be pregnancy loss at < 22 gestational weeks; no index pregnancy
-	Self report cases defined as pregnancy loss at ≥21 weeks gestation (previous not index)	Self-reported ever having a stillbirth (without specifying gestational weeks); no index pregnancy
-	-	No index pregnancy; random birth selected from available data
NA	-	NA

For peer review only

-	-	No index pregnancy; random birth selected from available data
-	-	No index pregnancy; random birth selected from available data
-	-	No index pregnancy; random birth selected from available data
-	-	No index pregnancy; random birth selected from available data
-	-	No index pregnancy; random birth selected from available data
-	-	No index pregnancy; random birth selected from available data
-	-	No index pregnancy; random birth selected from available data
-	-	Based on self-reported birth weight of first child
-	-	Based on self-reported birth weight of first child
-	-	No index pregnancy; random birth selected from available data
-	-	No index pregnancy; random birth selected from available data
-	-	NA
-	-	NA
NA	-	NA

For peer review only

NA	-	NA
NA	-	NA
-	-	NA
-	-	NA
-	-	NA
-	No last category of 6+ months	NA

For peer review only

FinnGen	GWAS
-	NA
-	-
Not possible to exclude pre-existing hypertension	NA
ICD-9 code also used, pre-existing diabetes included	-
ICD-10 O21, ICD-9 643, ICD-8 638	NA
NA	NA
NA	-
NA	NA
NA	NA
ICD-10 O03, ICD-9 634, ICD-8 643	NA
NA	NA
ICD-10 N96	NA
NA	NA
NA	NA
ICD-10 O42, ICD-9 6581A, ICD-8 63495	NA

For peer review only

ICD-10 O82	NA
NA	NA
NA	NA
NA	NA
NA	cases: <37 gestational weeks, controls: 39-42 gestational weeks, singleton live birth & a spontaneous onset of delivery without congenital malformations, healthy mothers
NA	cases: <37 gestational weeks, controls: 39-42 gestational weeks, singleton live birth & a spontaneous onset of delivery without congenital malformations, healthy mothers
ICD-10 O48, ICD-9 645	cases: >42 & <=44 gestational weeks, controls: 39-42 gestational weeks, singleton live birth & a spontaneous onset of delivery without congenital malformations, healthy mothers
NA	NA
NA	NA
NA	NA
NA	NA
NA	NA
NA	NA
NA	NA

For peer review only

NA	NA
NA	NA
NA	NA
NA	NA
NA	NA
NA	NA

For peer review only

Type	Outcomes	ALSPAC	BiB
Delivery	Birth weight	-	-
	Gestational age	-	-

Note: - no deviations from MR-PREG definition; NA: not available

For peer review only

Deviations from the collaboration definition		
**MoBa**	**UK Biobank**	**FinnGen**
-	Based on self-reported birth weight of first child; not possible to standardise by sex	NA
-	No index pregnancy; random birth selected from available data	NA

For peer review only

GWAS
NA
-

For peer review only

**Study name**	**Trait name**
**GenDiP**	Gestational diabetes
**EGG**	Gestational age
**EGG**	Pre-term birth
**EGG**	Post-term birth
**InterPregGen**	Pre-eclampsia
**PGC**	Post-natal depression

Note: numbers might not match with GWAS publica

For peer review only

Definition	Number of participants (N cases)
Cohort-specific definition, including data from health records, oral glucose tolerance tests and self-report	N= 351,624 (5,458 cases)
Days, estimated using either the last menstrual period date or ultrasound	N= 126,573
<259 days<br>ICD-10 O60	N= 220,964 (14,513 cases)
>294 days<br>ICD-10 O48	N= 124,328 (15,126 cases)
New-onset SBP ≥ 140 mmHg or DBP ≥ 90 mmHg on at least two occasion after 20th week gestation or ICD-10 O14 or O15	N= 162,879 (7,219 cases)
Cohort-specific definition of postpartum depression	N= 44,334 (10,863 cases)

ition due to further quality control taken in MR-PREG

For peer review only

Maternal/fetal genotype	Phenotype transformations
Maternal	Phenotype definition/covariates were not harmonized between GWAS due to differences in study design and availability of non-genetic information
Maternal/fetal	None specified
Maternal/fetal	None specified
Maternal/fetal	None specified
Maternal in all, maternal and fetal in 2 studies	None specified
Maternal	None specified

For peer review only

**Covariables**
PC adjustment in most studies, only additional covariates in 3 studies (age, age + BMI and age + genotyping array, respectively)
PCs or relationship matrix on autosomal chromosomes/chromosome X
PCs or relationship matrix on autosomal chromosomes/chromosome X
PCs or relationship matrix on autosomal chromosomes/chromosome X
Genotype counts from imputation, deCODE = county of origin, other cohorts = top five PCs as covariates, FINRISK = top twenty, Kazakhstan = top 10 PCs
PCs or relationship matrix on autosomal chromosomes/chromosome X

For peer review only

Cohorts needed to be excluded
ALSPAC, BIB, UK Biobank
ALSPAC, BiB, MOBA (~22k subsample)
ALSPAC, MOBA (~22k subsample), FinnGen (~104k subsample)
ALSPAC, MOBA (~22k subsample)
ALSPAC
ALSPAC, MOBA (~8k subsample), UKB

For peer review only
